# Assisted reproductive technologies (ARTs): Evaluation of evidence to support public policy development

**DOI:** 10.1186/1742-4755-11-76

**Published:** 2014-11-07

**Authors:** Alexa A Nardelli, Tania Stafinski, Tarek Motan, Kristin Klein, Devidas Menon

**Affiliations:** Health Technology and Policy Unit, School of Public Health, Department of Public Health Sciences, University of Alberta, Room 3021 Research Transition Facility, 8308 114 Street, Edmonton, Alberta T6G 2 V2 Canada; Division of Reproductive Endocrinology & Infertility, Department of Obstetrics & Gynaecology, Royal Alexandra Hospital, 10240 Kingsway Ave, Edmonton, AB T5H 3 V9 Canada; Faculty of Medicine and Dentistry, University of Alberta, Room 3021 Research Transition Facility, 8308 114 Street, Edmonton, Alberta T6G 2 V2 Canada

**Keywords:** IVF, ICSI, Assisted reproductive technology, Outcomes, Systematic review

## Abstract

Over the years, IVF/ICSI protocols have continued to evolve with efforts to improve outcomes. As a result, treatment success may be related to certain procedural factors, including number of embryos transferred and stage at which they are transferred. This review aims to assess the safety and effectiveness of IVF/ICSI in comparison to spontaneous conception and less invasive ARTs and the impact of procedure-related factors on the outcomes of IVF/ICSI in order to support the development of local clinical and policy guidance. Following Cochrane Collaboration guidelines and the PRISMA statement, a comprehensive systematic review of literature examining the impact of procedural characteristics on the safety or effectiveness of IVF/ICSI from 2007 to date was performed. 33 systematic reviews and 3 primary studies evaluating the impact of procedural differences, IVF/ICSI in comparison to less invasive ARTs, and ARTs in comparison to spontaneous conception were found. IVF was shown to offer significant benefits over no treatment and IUI in achieving pregnancy and live birth among couples with endometriosis or unexplained infertility. Frozen and blastocyst-stage embryo transfers were as effective as fresh and cleavage-stage embryo transfers, respectively. In comparison to single embryo transfer, double embryo transfer significantly increased pregnancy, live birth and multiple pregnancy/birth rates. IVF/ICSI was associated with more complications during pregnancy and delivery, and in infants compared to naturally conceived pregnancies, particularly when multiple embryo transfer was used. Frozen embryo transfer had fewer adverse events during pregnancy and delivery than fresh embryo transfer, and was at least as safe in terms of infant outcomes. The potential complications of IVF/ICSI may be minimized through procedural choices, but such choices often impact effectiveness. Thus, in developing clinical and policy guidance around IVF/ICSI, the risk-benefit trade-offs patients and providers are willing to accept must be carefully considered.

**Electronic supplementary material:**

The online version of this article (doi:10.1186/1742-4755-11-76) contains supplementary material, which is available to authorized users.

## Introduction

Infertility is a reproductive disorder defined clinically as the failure to achieve a clinical pregnancy following at least 12 months of unprotected sexual intercourse [1–3]. It can be related to female factors (35% to 40% of couples), male factors (20× to 40% of couples), both (20% to 30% of couples), or remain unexplained [4–6]. In women, it is commonly caused by ovulatory dysfunction, tubal obstructions, and/or endometriosis. In men, it is often a result of abnormalities in sperm production and function or sperm duct blockages.

Management includes fertility counselling, lifestyle modifications, medical/surgical treatment of underlying conditions, fertility medications, and assisted reproductive technologies (ARTs), such as intrauterine insemination (IUI) and in vitro fertilization (IVF)/intracytoplasmic sperm injection (ICSI). In recent years, efforts to optimize maternal and infant outcomes have focused on IVF/ICSI and specific procedure-related factors, such as the number of embryos transferred and whether sperm, eggs or embryos used should be fresh or frozen [7–10].

Parallel to these developments in technological innovation, governments in many countries have been facing demands to fund and/or regulate the provision of ARTs services, so as to reduce adverse events and complications and improve maternal and child outcomes. In particular, in the province of Alberta, the government has been considering options for the public funding of IVF. This has necessitated the examination of evidence relating to the safety and effectiveness of existing ARTs services (which are currently paid for out-of-pocket), and of the procedure-related factors relevant to these services, in order to inform the development of public policy options.

The objective of this review was to determine the current state of the science related to the safety and effectiveness of IVF/ICSI in comparison to natural conception and less invasive ARTs that are available in the province and the influence of procedure-related factors on the safety and effectiveness of IVF/ICSI.

## Methods

A systematic review of relevant published systematic reviews and primary studies (when systematic reviews were not available) addressing the safety and effectiveness of IVF/ICSI in comparison to natural/spontaneous conception (SC) and less invasive ARTs and the impact of key procedure-related factors on the safety and clinical effectiveness of IVF/ICSI) was performed following Cochrane Collaboration guidelines and the PRISMA statement [11, 12].

Currently, the ARTs services available in Alberta are: ovulation induction, IUI, and IVF/ICSI. Based on these, and a recent review of IVF funding policies around the world, 2 important comparisons of procedures were identified for review: 1) IVF/ICSI in comparison to SC, and 2) IVF/ICSI in comparison to less invasive ARTs (ovulation induction and IUI) [13]. In addition, 4 comparisons of different factors associated with IVF/ICSI were also identified: 1) single versus multiple embryo transfers in IVF/ICSI, 2) fresh versus frozen embryo transfers, 3) blastocyst versus cleavage stage embryo transfers, and 4) autologous versus donor embryo transfers. The current review focuses on the impact of each of these factors on the: 1) safety of IVF/ICSI, including pregnancy/delivery (obstetrical) complications (ectopic pregnancy, gestational diabetes, preeclampsia, placental complications, preterm birth, and caesarean delivery) and neonatal/infant complications (low birth weight, neonatal or perinatal mortality, neonatal intensive care unit admissions, birth defects and congenital malformations), and 2) the effectiveness of IVF/ICSI, including rates of cycle cancellation, pregnancy, miscarriage, live birth and multiple pregnancy/birth. At the first stage, a search for systematic reviews synthesizing primary studies was conducted. A review of additional primary studies was also planned as a second stage if no existing published review on the outcomes of interest for any comparison was found. Details of the identification and selection of information sources (including eligibility criteria) are provided below.

### Identification and selection of relevant papers

#### Stage 1: identification of systematic reviews

A literature search for systematic reviews describing the safety or effectiveness of IVF/ICSI published in English within the last 5 years was conducted. A structured search strategy, which combined relevant controlled vocabulary terms, such as Medical Subject Headings (e.g., Reproductive Techniques, Assisted; Fertilization in Vitro; Embryo Transfer) with additional non-index keywords (e.g., assisted reproduct*; IVF), was developed and then applied to the following bibliographic databases: PubMed (MEDLINE and non-MEDLINE sources), EMBASE, The Cochrane Library, the Centre for Reviews & Dissemination (DARE, NHS EED, and HTA databases), Web of Science, Scopus, CINAHL, and PsycINFO. The search was performed in January 2012 and updated monthly until March 2013. The selection criteria (including the PICO questions) for reviews found at this stage are provided in Table 1. Any systematic review that included patients 18 years or older with infertility undergoing IVF/ICSI or one of the comparator interventions listed in Table 1 were included. Through this process, no systematic reviews were found that assessed the impact of: 1) IVF/ICSI (versus SC) on multiple pregnancy/birth rates, 2) less invasive ARTs (versus IVF/ICSI) on neonatal/infant complications, 3) the state of embryos (fresh or frozen) on multiple pregnancy/birth rates, 4) the stage of embryos (blastocyst or cleavage) on pregnancy/delivery complications or neonatal/infant complications, and 5) the source of embryos (autologous or donor).

**Table 1:** **PICOS elements of the review protoco**

Parameter	Inclusion criteria	Exclusion criteria
***P*** *articipants*	• Couples 18 years of age and older with infertility	
***I*** *nterventions*	• IVF/ICSI	• GIFT/ZIFT
		• In vitro maturation
***C*** *omparators*	• Spontaneous/natural conception	Studies comparing different drugs or drug regimens used
	• Less invasive ARTs (ovulation induction, intrauterine insemination)	• Studies assessing pre-treatment characteristics, such as embryo and uterine preparation techniques or hysteroscopy, or treatment ‘add-ons’, such as preimplantation genetic screening (PGS) or assisted hatching (AH)
	• Procedural differences, including:	
	- the number of embryo’s transferred	
	- blastocyst vs. cleavage embryo transfers	
	- frozen vs. fresh embryo transfers	
	- autologous vs. donor embryo transfers	
***O*** *utcomes*	Safety:	• Studies without any defined clinical outcomes
	• Neonatal/infant complications (e.g., ectopic pregnancy, low birth weight, neonatal/perinatal mortality, birth defects, congenital malformations)	
	• Pregnancy and delivery complications (e.g., OHSS, ectopic pregnancy, preeclampsia, caesarean delivery, preterm birth)	
	Effectiveness:	
	• Indicators of cycle success (e.g., number of oocytes retrieved, cycle cancellation, implantation)	
	• Pregnancy, miscarriage, live birth	
	• Multiple pregnancy/multiple birth	
***S*** *tudy design*	• Systematic reviews	Primary studies*

*Primary studies will be included if evidence gaps are identified after review of systematic reviews.

To identify unpublished evidence, Google, grey literature databases, and web sites of guidelines, clinical trials, health technology assessment agencies, and key ARTs-related international and national organizations were searched. In addition to the electronic searches, the reference lists of relevant articles were scanned and clinical experts in Alberta were contacted. Search results were imported into Reference Manager and duplicate citations were removed. The details of the literature search are provided in Additional file 1: Table S1.

#### Stage 2: identification of primary studies

Based on the predefined comparisons and outcomes of interest described above, available evidence from the included systematic reviews was assessed to identify any evidence gaps. Since no systematic reviews for some comparisons (as described in the next section) were found, an additional search for primary studies was performed. A structured search strategy similar to that used to search for reviews as described above was applied to PubMed in January 2012 and updated monthly until March 2013 (see Additional file 1: Table S1).

The titles and abstracts of all search results were independently reviewed by two researchers using a standard checklist with predetermined study inclusion and exclusion criteria (see Table 1 for a list of inclusion/exclusion criteria). Disagreements were resolved through discussion and third party review. Concordance between researchers was assessed using the Kappa statistic [14].

### Data extraction and critical appraisal of included papers

Each researcher independently extracted information from selected reviews and primary studies using a standard, pre-tested data abstraction form and a set of decision rules. The form contained elements for assessing the purpose, methods, findings, and quality of each paper. The quality of each systematic review was determined using the Oxman and Guyatt index of scientific quality scoring system for systematic reviews [15, 16], a widely validated scale. For primary studies, each was critically appraised using the Oxford Levels of Evidence Scale [17]. The overall quality of evidence was assessed using the GRADE approach [18].

### Synthesis of results

Information collected from the systematic reviews and primary studies were summarized in tables to facilitate comparative analyses of any variations in methodological parameters. The findings were then used to examine heterogeneity across papers and determine whether meta-analyses were feasible. Where clinical heterogeneity precluded presentation of pooled estimates, the results from papers were summarized qualitatively. Given the heterogeneity between studies, a meta-analysis was not performed.

## Results

### Results of literature search

**Figure 1:**
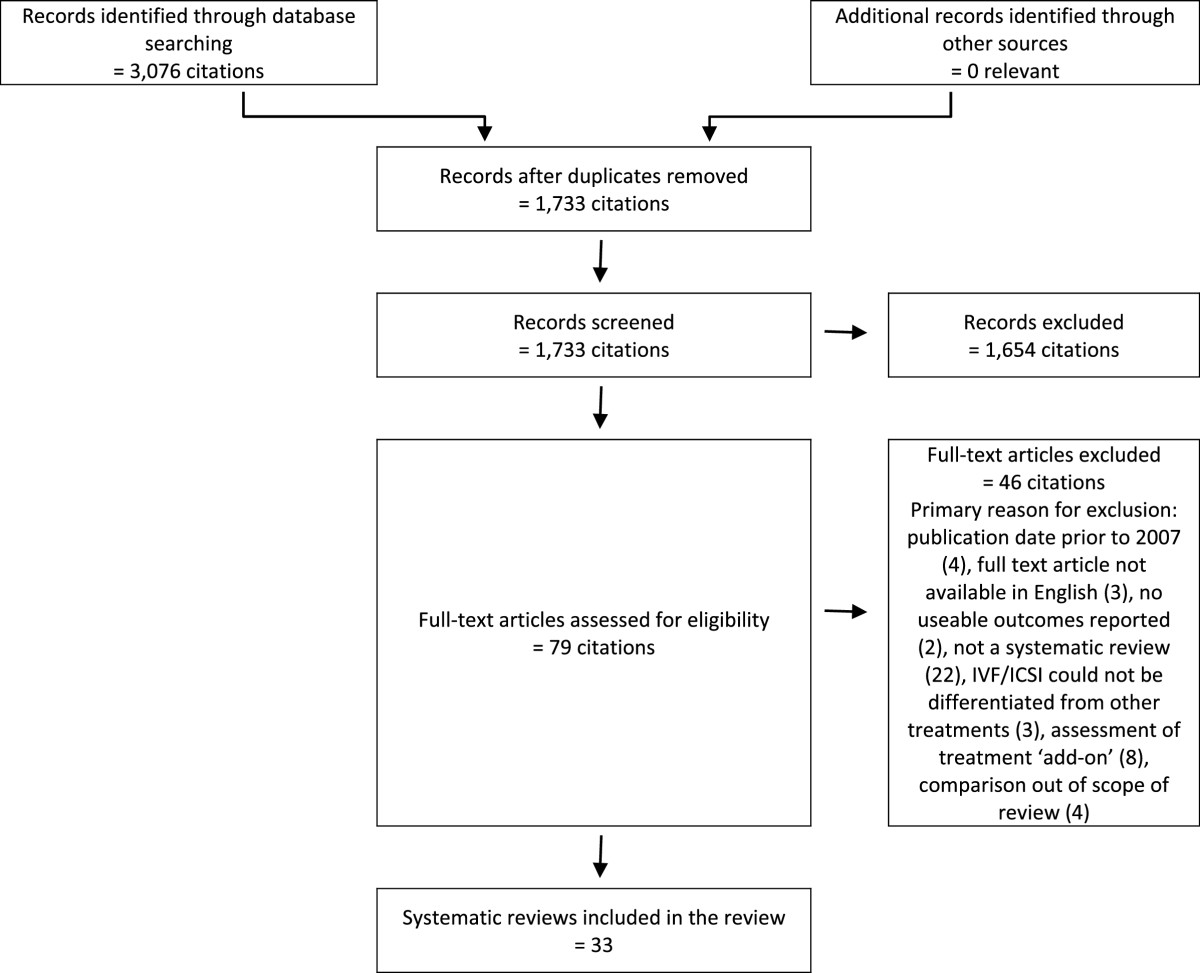
**PRISMA flowchart of literature search results and study selection for safety and clinical effectiveness review: systematic reviews.**

**Figure 2:**
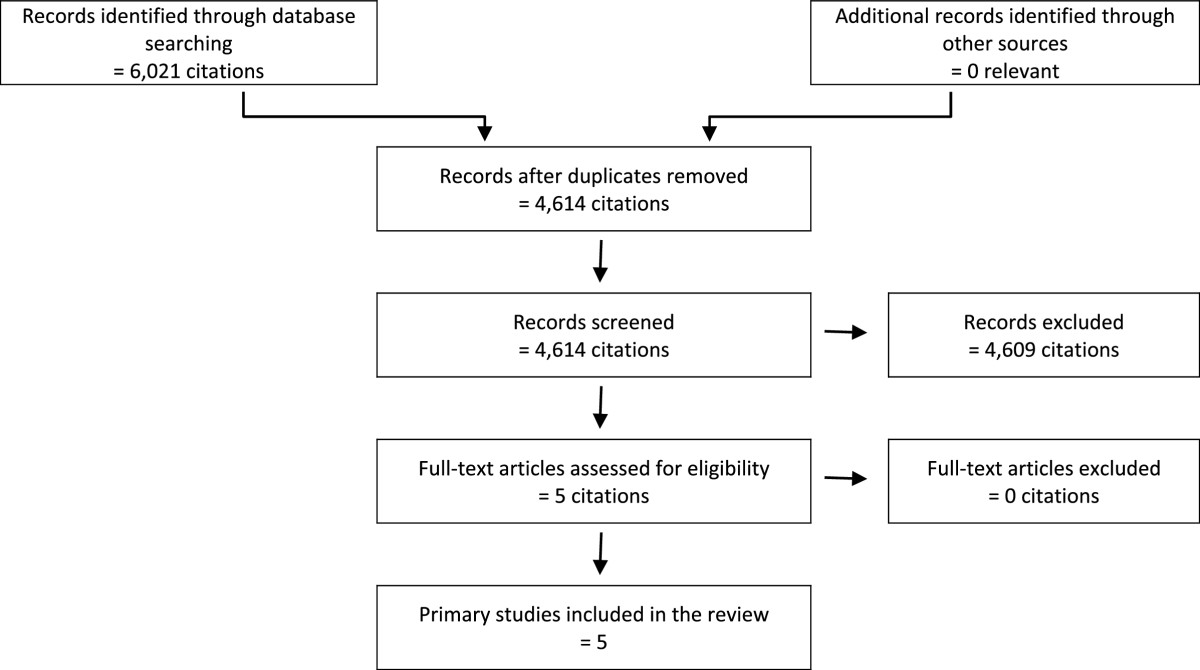
**PRISMA flowchart of literature search results and study selection for safety and clinical effectiveness review: additional primary studies.**

1,733 discrete citations were identified through the literature search for systematic reviews, of which 79 potentially relevant systematic reviews were selected for full review (Figure 1). Thirty-three met the inclusion criteria. The search for additional primary studies yielded 4,614 discrete citations (Figure 2). Three potentially relevant studies were selected for full review, all of which met the inclusion criteria. Excluded studies and their reasons for exclusion are presented in Additional file 2: Table S2. Methodological characteristics and outcomes of each included study are summarized in Additional file 3: Table S3, Additional file 4: Table S4, Additional file 5: Table S5; Additional file 6: Table S6, Additional file 7: Table S7, Additional file 8: Table S8, Additional file 9: Table S9, Additional file 10: Table S10, Additional file 11: Table S11, Additional file 12: Table S12, Additional file 13: Table S13, Additional file 14: Table S14, Additional file 15: Table S15 and Additional file 16: Table S16.

### Overall description of included studies

Three primary studies and 33 systematic reviews, of which, 24 included meta-analyses, were included. As described below, they evaluated the safety and effectiveness of IVF/ICSI in comparison to SC and less invasive ARTs, and the effect of the number, stage (blastocyst or cleavage), state (fresh or frozen) and source (autologous or donor) of embryos transferred on the safety and effectiveness of IVF/ICSI (see Table 2).

**Table 2:** **Assessment of available evidence: number of included systematic reviews and primary studies and GRADE rating of quality of evidence for predefined comparisons and outcomes of interest**

Outcome of interest	Spontaneous conception (17 reviews; no additional primary studies)	Less invasive ARTs – OI/IUI (2 reviews; no additional primary studies)	Number of embryos (6 reviews; no additional primary studies)	State of embryos – fresh/frozen (5 reviews; no additional primary studies)	Stage of embryos – blastocyst/cleavage (5 reviews; 2 additional primary studies)	Source of embryos – autologous/donor 1 review; 1 additional primary study)
Effectiveness:						
• Cycle success (cancellation, implantation)	*	*	2 reviews	*	2 reviews	*
			Low to Moderate		Moderate	
• Pregnancy, miscarriage, and live birth	1 review	1 review	6 reviews	2 reviews	2 reviews	1 primary study
	Moderate	Moderate	Low to Moderate	Low to Moderate	Moderate	Low
• Multiple pregnancy/birth	*	1 review	3 reviews	*	2 reviews	*
		Moderate	Low to Moderate		Moderate	
Safety:						
• Pregnancy and delivery complications	5 reviews	1 review	3 reviews	5 reviews	2 primary studies	1 review
	Very Low to Low	Very Low to Moderate	Low to Moderate	Very Low to Moderate	Low	Low to Very Low
• neonatal/infant complications	15 reviews	*	2 reviews	3 reviews	2 primary studies	1 review
	Very Low to Low		Low to Moderate	Low to Moderate	Low	Low to Very Low

*Blanks indicate no reviews or primary studies were found.

#### IVF/ICSI in comparison to SC

In 17 of the reviews, ARTs was compared to SC (i.e., conception not preceded by an ARTs intervention) [19–35]. Among them, 11 also presented pooled estimates from quantitative analyses of at least one of the outcomes. The majority of studies comprising the reviews were retrospective or prospective cohorts with the general population serving as the ‘control’ group, rather than infertile couples who had eventually achieved pregnancy without the use of assisted conception. Of the 17 reviews, 1 reported pregnancy and live birth rates, 6 reported pregnancy/delivery complications and 15 reported neonatal/infant complications, focusing on short- and long term infant outcomes, ranging from neonatal periods through infancy and childhood. Outcomes at adolescence and adulthood were examined in 6 reviews. Population and procedural characteristics were rarely reported. No reviews or additional primary studies reporting on the effect of IVF/ICSI on multiple pregnancy birth rates were found.

#### IVF/ICSI in comparison to less invasive ARTs treatment options

IVF/ICSI was compared to less invasive ARTs in 1 meta-analysis and 1 review [31, 36]. The meta-analysis comprised 6 RCTs (published 1993 to 2011) comparing OHSS, pregnancy, multiple pregnancy and live birth rates after IVF/ICSI versus IUI (4) or SC (2) [31]. Where RCTs reported treatment protocols, IVF/ICSI was preceded by a GnRHa protocol with hMG or FSH and involved the transfer of 1–4 blastocyst or cleavage stage embryos, whereas clomiphene citrate (CC) or gonadotropins (e.g., FSH) were used for controlled ovarian stimulation (COS) in IUI. In 3 of the 4 comparing IVF/ICSI to IUI, women who had not previously received ARTs underwent: 1) up to 2 cycles of IVF (1 fresh cycle and 1 frozen-thaw cycle) vs. up to 3 cycles of IUI, 2) up to 6 cycles of IVF vs. up to 6 cycles of IUI, or 3) 1 cycle of IVF vs. up to 3 cycles of IUI. In the fourth study, all couples received up to 3 cycles of IUI with CC as a first line of treatment. Women who failed to achieve an ongoing pregnancy after these 3 cycles underwent either: 1) up to 6 cycles of IVF, or 2) up to 3 cycles of IUI with FSH and, if no pregnancy is achieved with FSH-IUI, up to 6 cycles of IVF.

The review that did not contain a meta-analysis included 47 primary studies (mostly case–control and cohort studies) examining pregnancy complications after different types of ARTs. However, no further details of these studies were provided [36]. No reviews or additional primary studies reporting neonatal/infant complications after IVF/ICSI in comparison to less invasive ARTs were found.

#### Number of embryos transferred

Six meta-analyses spanning a total of 25 unique primary studies (14 RCTs, 1 quasi-RCT, and 10 cohort studies published between 1994 and 2010) evaluated the effect of the number of embryos transferred on the safety and effectiveness of IVF/ICSI [30, 37–41]. One review evaluated the association between fresh, autologous IVF/ICSI and several patient and procedural factors pre-identified as predictors of IVF/ICSI success, one of which was the number of embryos transferred [41]. One review evaluated the association between fresh, autologous IVF/ICSI and several patient and procedural factors pre-identified as predictors of IVF/ICSI success [41]. A total of 14 studies were included, mainly retrospective cohorts, published between 1997 and 2008. The number of embryos transferred was assessed in 7 studies and the quality of embryos transferred in 3 studies. In one meta-analysis, individual patient data were used [37]. Five meta-analyses compared double embryo transfer (DET) to elective single embryo transfer (eSET), and one also assessed higher order multiple embryo transfers and limited analyses to fresh embryos only. Three excluded blastocyst-stage embryos, and one excluded donor oocytes or embryos, although the use of donors was not explicitly stated in any of the pooled studies. Across these studies, similar COS protocols had been used (GnRHa + hMG or FSH), and most only included one cycle of IVF/ICSI per couple. Over half specified maternal upper age limits (ranging from 30 to 37 years) and 7 of them further limited inclusion to women deemed to have a “good prognosis” (i.e., younger women in their 1^st^-2^nd^ IVF/ICSI cycle with good embryo quality).

#### Fresh versus frozen embryo transfer (FET)

The safety and effectiveness of FETs compared to fresh embryo transfers was assessed in 5 reviews of 83 unique primary studies published between 1993 and 2011 [42–46]. Four also presented meta-analyses. However, one was based on a single study and another focused exclusively on the incidence of ectopic pregnancy [42]. The remaining two assessed the effect of FET on pregnancy and miscarriage (3 RCTs), and maternal and infant safety in singleton pregnancies (11 cohorts) [45, 46]. Where reported, included studies used similar COS protocols (GnRHa + hMG and/or FSH in the majority), and evaluated autologous cleavage- and blastocyst-stage embryo transfers in ‘unselected’ women (all women receiving fertility treatment in a certain area or clinic) or women expected to have a good prognosis. The fifth review, which did not perform a meta-analysis, assessed 67 studies, 25 comparing the transfer of frozen cleavage-stage embryos to fresh cleavage-stage embryos or SC (1 RCT, 12 retrospective cohorts, and 12 registry reports) [44]. The remaining 42 were non-comparative and evaluated the transfer of frozen blastocyst-stage embryos or the fertilization and transfer of frozen oocytes. In most, details of clinical protocols used were not reported. No reviews or additional primary studies reporting the effect of fresh versus frozen embryo transfer on the incidence of multiples were found.

#### Stage of embryo during transfer

Four meta-analyses focused on the effect of developmental stage of the embryo during transfer, comparing cleavage-stage embryo transfer, where embryos were transferred 2–3 days after fertilization, to blastocyst-stage embryo transfer, where embryos were transferred 5–6 days after fertilization [47–51]. Three limited inclusion to RCTs. The fourth incorporated any comparative study reporting on sex ratio and monozygotic twinning (MZT), regardless of its design. All 4 meta-analyses considered fresh cycles only, and in 2, autologous oocytes only. Collectively, they involved a total of 38 distinct primary studies (18 RCTs) published between 1987 and 2007. Across these studies, there was little difference in COS protocols reported (mainly GnRHa + hMG and/or FSH), and in most, the use of donor oocytes was not explicitly mentioned, nor was the number of embryos per cycle or the number of cycles per woman. Where reported, numbers varied across studies. In 9, only women deemed likely to succeed with blastocyst transfers were included, and in 2, only women with a poor prognosis were included. The rest of the studies involved ‘unselected’ couples where only maternal upper age limits ranging from 35 to 44 years had been applied. Safety data were limited. However, 2 primary studies (retrospective cohorts published in 2012 and 2013) which assessed the impact of embryo stage on obstetric and perinatal complications after IVF provided such information [52, 53].

#### Donor embryo transfer

One systematic review assessing the safety of IVF/ICSI using donor embryos was included [54]. Details of the 79 primary studies covered in the review were not provided. A review of the clinical effectiveness of donor IVF in comparison to autologous IVF was not found. Therefore, one recent primary study assessing the effectiveness of donor IVF/ICSI in 6 countries with national ART surveillance programs was included [55]. The study compared data from over 1 million autologous IVF cycles to over 100,000 donor cycles across Australia/New Zealand, Canada, Finland, the UK, and the US [55]. No reviews or additional primary studies assessing the impact of donor embryo transfer on cycle cancellation or multiple births were found.

### Overall quality of included studies

**Figure 3:**
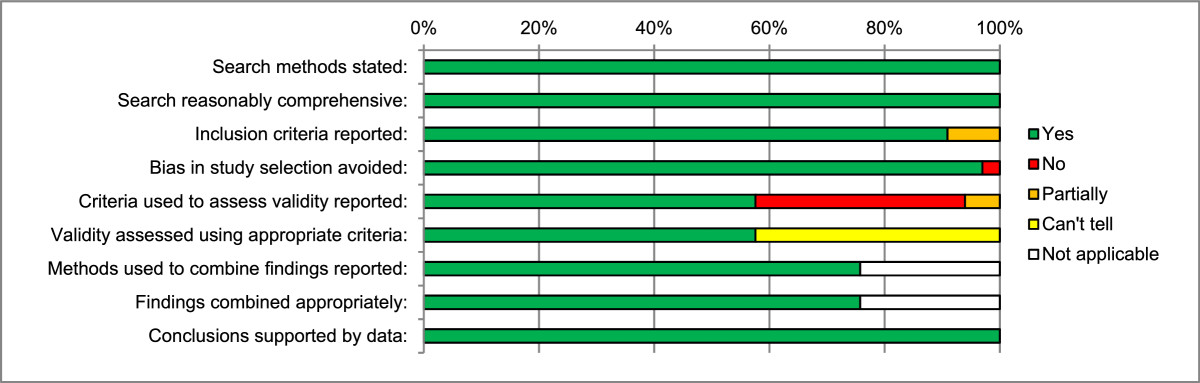
**Quality of systematic reviews: Oxman and Guyatt index of scientific quality for systematic reviews.**

Results of quality assessment for the systematic reviews are presented in Additional file 5: Table S5 and Figure 3. In general, most of the systematic reviews were of high quality, regardless of whether meta-analyses had been performed. All provided details of their search strategy, which was comprehensive, and nearly all clearly described their study inclusion criteria. Aside from one review that excluded studies with 0% incidence of a primary outcome, no clear bias in study selection was found. The most common weakness of the reviews was failure to perform or report a validity assessment of included studies (12/33). However, where validity was assessed, appropriate criteria were used. Of reviews with meta-analyses (24/33), all recorded methods used to combine outcomes data, and all were appropriate. Comparing studies that pooled data with those that did not, there was variation in the degree of clinical heterogeneity deemed too much to pool. Conclusions drawn in all reviews were consistent with the data they collected and reported.

The overall rating of the quality of evidence according to the GRADE scale for each comparison and outcome of interest is shown in Table 2. For most of the procedural comparisons addressed in the reviews and additional primary studies, data from experimental trials were available and supplemented with data from prospective and retrospective cohorts. Studies assessing the effectiveness of IVF/ICSI in comparison to spontaneous conception and less invasive ARTs were of ‘moderate’ quality. Similarly, the available evidence on the effectiveness of IVF/ICSI by the stage of embryo transferred was ‘moderate’, while both ‘low’ and ‘moderate’ quality studies comprised the body of evidence on the effectiveness of IVF/ICSI by the number and state (fresh or frozen) of embryos transferred. With respect to pregnancy and delivery complications, some ‘moderate’ studies were available. However, most of the evidence was ‘low’ quality. The poorest quality data were around infant outcomes after ARTs compared to SC (mostly ‘very low’ quality, some ‘low’ quality). They originated from small studies that typically compared ART-conceived infants to either the general population or ‘spontaneously’ conceived infants. Therefore, it was difficult to rule out the use of less invasive ARTs treatments in control groups, as well as the potential influence of infertility and other population differences.

Within each review, patient populations and treatments varied across included studies. Among reviews of the same comparisons, there was overlap in selected studies (multiple reports on the same studies or patients published by different investigators). The majority of reviews did not identify or discuss such overlap.

### Safety

#### IVF/ICSI in comparison to SC

##### Pregnancy/delivery complications

In two reviews, increased risks of ectopic pregnancy (EP) (10% vs. 2%), gestational diabetes (GD) (10% vs. 6%), pregnancy-induced hypertension (PIH) (6% vs. 3%), placenta praevia/placental abruption (4% vs. 1%), preterm premature rupture of membranes (proportions not reported), caesarean delivery (proportions not reported), and preterm delivery (PTD) (8% vs. 5%) were observed in women who underwent IVF/ICSI with SET or achieved a singleton pregnancy through IVF/ICSI compared to those who conceived ‘spontaneously’ [29, 30]. These risks remained even after studies with spontaneous pregnancies in infertile couples had been excluded through sensitivity analyses.

A significant increase in the risk of preterm birth (PTB) after IVF/ICSI in comparison to SC was further confirmed in 4 meta-analyses of studies controlling for at least maternal age, among other potential confounding factors [20, 22, 23, 32]. Across the studies that controlled for basic maternal characteristics (e.g., age, parity), women who conceived twins through ARTs were more likely to undergo a caesarean section delivery than women who conceived twins spontaneously (OR:1.7) [20]. Those adjusting for at least maternal age indicated that singletons born to infertile women without the use of assisted conception had a significantly higher risk of PTB and low birth weight (LBW) than singletons born to fertile women [32].

##### Infant/neonatal complications

Indirect comparisons in 1 review showed MZT rates following ARTs approached 2%, which was over double that found after SC (0.4%) [25]. This rate was further amplified in FETs (3%) and blastocyst embryo transfers (5%). Pooled data from reviews controlling for at least maternal age in singletons and twins separately revealed a significantly increased odds of being born LBW after IVF/ICSI in both groups, with a more distinct effect in singletons (OR:1.6-singletons/1.1-twins) [22, 23, 29]. Furthermore, IVF/ICSI singletons, but not twins, were small for their gestational age compared to SC singletons and twins (OR 1.5). In another analysis of twins only, no significant differences in LBW, neonatal intensive care unit (NICU) admission, or perinatal mortality (PNM) rates were seen between IVF/ICSI twins and SC twins, with the exception of a subgroup of twins of different sexes [20]. Conversely, singletons conceived through IVF/ICSI were at almost double the risk of PNM and 1.6 times the risk of NICU admission than singletons conceived spontaneously (p < 0.05) [29].

Across reviews, birth defect and congenital malformation rates in infants born after IVF/ICSI were significantly higher than those in SC infants (pooled OR:1.3-2.0), while no differences in imprinting disorders were noted [19, 20, 26, 28, 29, 33]. When ART singletons and multiples had been considered separately, congenital malformation rates were significantly higher in both groups (OR:singletons-1.7/multiples-6.7) compared to SC singletons and multiples, but birth defect rates were no different between ART multiples and SC multiples.

Three reviews reported that couples who conceived via IVF/ICSI were over twice as likely to have an infant with cerebral palsy, in comparison to couples who conceived naturally [24, 27, 35]. This difference held for singletons, while no significant differences between twins were observed. Evidence around the risk of autism spectrum disorders (ASD) in IVF/ICSI children was variable. In one review, 3 of 5 studies found no differences between IVF/ICSI conceived and SC infants, one reported a significant increase in the incidence of a broader group of psychiatric disorders (of which 1 disorder was ASD) in IVF/ICSI children, and one showed significantly reduced chances of having ASD following IVF, after adjusting for several factors [24].

Among studies included across 4 reviews that assessed developmental delay, most reported no significant differences in motor (15/17 studies), emotional/behavioral (14/19), cognitive (11/15), or mental (10/11) development between infants and children born from assisted conception compared to SC [24, 27, 34, 35]. Similar growth patterns between ART-conceived and spontaneously conceived children, adolescents, and adults were observed [21, 34]. Chronic disease profiles in children and adolescents were also similar, with the exception of a higher prevalence of attention-deficit/hyperactivity disorder, depression, binge drinking, and cancer after ARTs, which were reported in one study [35].

#### IVF/ICSI in comparison to less invasive ARTs treatment options

##### Pregnancy/delivery complications

One review compared the incidence of ovarian hyperstimulation syndrome (OHSS) in women undergoing IVF to that in women undergoing IUI with COS, and found no difference [31]. In a review of hypertensive complications, after adjusting for age and smoking status among other factors, women who conceived through IVF/ICSI/GIFT/ZIFT, but not ovulation induction or IUI, were significantly more likely to have preeclampsia than women who conceived spontaneously (OR:1.8-2.7) [36]. However, where analyses had controlled for maternal age, gestational age and parity, the odds of experiencing GD, PIH, and caesarean delivery were found to be significantly higher not only after IVF but also after IUI in comparison to SC [36].

##### Neonatal/infant complications

In a single review of rates of congenital malformations, no significant differences between women who conceived through ovulation induction with or without IUI and those who conceived spontaneously were found [28] However, IUI was not compared to IVF/ICSI.

#### Number of embryos transferred

##### Pregnancy/delivery complications

The results of one meta-analysis indicated that compared to SET, DET was associated with higher rates of preterm rupture of membranes (8.5%), placental abruption (2.2%), and preeclampsia (7.7%) (compared to 0.8%, 0%, 7.0%, respectively; statistical significance not reported) [30]. With respect to the incidence of GD, findings were inconsistent. No significant differences between SET and DET in ectopic pregnancy and placenta praevia were found [30, 38].

Findings from a meta-analysis of RCTs showed a significantly lower risk of PTD after SET in comparison to DET (OR:0.3-0.4) [30, 37]. However, those from a meta-analysis of cohort studies showed no significant difference [30, 37]. Further, in one cohort study included in this review, more women underwent caesarean section delivery after DET (24%) compared to SET (20%) (significance not reported).

##### Neonatal/infant complications

The results of two reviews indicated that infants born after IVF/ICSI with SET were 2–4 times less likely to be born LBW compared to infants born after DET (pooled OR:0.3-0.5) [30, 37]. In one of the reviews, a single RCT reported higher rates of neonatal mortality (NNM) (1.1% vs. 0%), PNM (1.0% vs. 0.7%), and congenital malformations (4.8% vs. 3.1%) after DET (significance not reported), while pooled data from cohorts demonstrated no significant difference in neonatal or PNM, or NICU admissions between SET and DET [30].

#### Fresh versus FET

##### Pregnancy/delivery complications

In two reviews, no significant differences in the number of ectopic pregnancies or women admitted to the hospital during their pregnancy after fresh versus FETs were observed [42, 43]. One review included a study in which slightly more women developed OHSS after fresh versus FET, but differences did not reach statistical significance. In meta-analyses limited to singleton pregnancies, a significantly lower incidence of antepartum hemorrhage and PTB after FET (9% and 3%, respectively) compared to fresh embryo transfer (11% and 5%, respectively) was found [45]. However, the incidence of caesarean delivery after frozen cycles was significantly higher (35% vs. 29%) [45]. Combined data from singletons demonstrated significantly lower PTB rates after FET in comparison to fresh embryo transfer (RR:0.9), but for twins, the results varied [32, 44].

##### Neonatal/infant complications

In singletons born after IVF/ICSI, FETs were consistently associated with a lower risk of LBW than fresh embryo transfers (pooled RR:0.69) [45]. For twins, three studies included in a review reported a reduced incidence of LBW after FETs (38-47%) compared to fresh transfers (50-55%), while two found no difference [44].

Two reviews indicated that the rates of stillbirths and NICU admissions after FET were lower and similar, respectively [44, 45]. Further, pooled data in one review showed no significant differences in congenital malformations between fresh and FET [45]. Long-term growth patterns were normal and similar between fresh IVF/ICSI infants and frozen IVF/ICSI infants, and between these 2 groups and a group of SC controls [44]. Conversely, early delays in growth were demonstrated in both fresh and frozen IVF/ICSI groups in comparison to SC multiples. However, these differences dissipated after 6 months of age.

#### Stage of embryo during transfer

##### Pregnancy/delivery complications

One of 9 comparative studies comprising a review showed significantly higher rates of MZT after blastocyst transfers compared to cleavage transfers [47]. Other than this finding, no review discussed differences in safety between blastocyst- and cleavage-stage embryo transfers. Two recent primary studies, one on over 12,000 singleton IVF births from a Canadian ART registry and one on over 4,000 singleton IVF births in Australia, performed analyses of obstetric and perinatal complications, adjusting for several maternal and procedural characteristics [52, 53]. No significant differences in preeclampsia, antepartum/postpartum hemorrhage, placenta previa, placental abruption, after blastocyst versus cleavage-stage transfers were seen. However, one found higher chances of PTB associated with blastocyst-stage transfers (17%) in comparison to cleavage-stage transfers (14%; OR:1.3; p < 0.001) [53].

##### Neonatal/infant complications

In the two primary studies reporting infant outcomes, no significant differences in LBW, size for gestational age, congenital anomalies, stillbirth, or neonatal death blastocyst- and cleavage-stage embryo transfers were found [52, 53].

#### Donor embryo transfer

##### Pregnancy/delivery complications

One systematic review demonstrated that, in comparison to autologous IVF/ICSI cycles, first trimester vaginal bleeding and hypertensive complications were significantly higher in pregnancies resulting from donor IVF/ICSI cycles (even when age and parity were taken into account), in 2 and 5 studies, respectively [54]. No significant differences between donor cycles and PTD were observed [54].

##### Neonatal/infant complications

The same review found no significant differences in LBW rates, proportion of infants small for their gestational age, or congenital malformation rates between infants conceived through donor IVF versus those conceived through autologous IVF [54].

### Efficacy/effectiveness

#### IVF/ICSI in comparison to SC

##### Pregnancy and live births

Based on a meta-analysis of 2 RCTs, the odds of achieving a clinical pregnancy after 1 cycle of IVF were over 3 times higher than those after 3–6 months of no treatment [31]. Further, very few couples (4%) with unexplained infertility had a live birth after 3–6 months of no treatment, whereas almost half of couples with unexplained infertility achieved a live birth after 1 cycle of IVF (OR:22.0).

#### IVF/ICSI in comparison to less invasive ARTs treatment options

##### Pregnancy and live births

Results from one meta-analysis indicated no significant differences in pregnancy or live birth rates after IVF and stimulated IUI (sIUI) in couples with unexplained infertility. However, to achieve the same pregnancy and live birth rates rates, up to 3 cycles of IUI were required, compared to 1 cycle of IVF [31]. In contrast, pregnancy and live birth rates were significantly different between IUI and IVF when IVF was used as a second line of treatment in couples who were unable to succeed with 3 cycles of IUI with CC (84% of women became pregnant within 6 cycles of IVF (19% miscarriages) in comparison to 30% of women within 3 cycles of IUI with FSH (14% miscarriages) (OR:12.8-pregnancy/2.7-live birth)).

##### Multiple pregnancies/births

The same meta-analysis demonstrated similar multiple pregnancy rates after sIUI and IVF. However, multiple pregnancies in the IVF group were found to occur only in couples who received more than 1 embryo [31]. Further, multiple pregnancy rates appeared to be lower after 1 cycle of elective SET IVF (14%) compared to 3 cycles of IUI (25%), but statistical significance was not reported.

#### Number of embryos transferred

##### Cycle success

Both reviews examining the relationship between number of embryos transferred and number successfully implanted found that success rates were similar, regardless of whether 1 or 2 had been transferred [38, 39].

##### Pregnancy miscarriages and live births

In contrast, clinical and ongoing pregnancy rates per couple after DET were shown to be almost 2 times greater than pregnancy rates after SET, even where only women under 36 years of age were considered (pooled RR:1.4-2.2, 1.9-2.1, respectively) [38–40]. In another review, significantly higher pregnancy rates were seen in women who received more than two embryos in comparison those who received one or two embryos [41]. Further, in a single small RCT included in one of the reviews which compared women undergoing one IVF/ICSI cycle with DET to those undergoing 2 SET cycles (one fresh SET cycle and one frozen SET cycle), one three embryo transfer (TET) cycle, or one four embryo transfer (QET) cycle, no differences in clinical pregnancy rates were observed [40]. In most of the reviews, the risk of miscarriage between DET and SET or eSET groups was shown to be similar [30, 37, 38, 40].

Based on the results of meta-analyses, while cumulative live birth rates per couple were found to be comparable between DET and SET, live birth rates per cycle after DET were significantly higher than those after SET, with odds ratios ranging from 1.6-2.1, after adjusting for the cause of infertility, treatment characteristics, and the quality of embryos transferred [37–40]. This effect did not change during subgroup analyses of individual patient data comparing women under 33 years of age to those 33 and older, couples with less than 3 years of infertility to those with 3 or more years of infertility, and top quality embryos to lesser quality embryos [37]. The probability of a live birth was also higher in TET (27%) and QET (54%) compared to DET (13% and 29%, respectively), but differences did not reach statistical significance.

##### Multiple pregnancies/births

In one meta-analysis of 8 RCTs, the odds of carrying multiples were found to be greater in women who achieved a clinical pregnancy through DET than in women who received SET/eSET (19%-30% vs. 1%-2%; OR:17–25; p < 0.05) [40]. In 1 of the RCTs included in this analysis, the incidence of multiples was also higher in TET compared to DET (RR:0.17 (0.01, 3.85)), QET compared to DET (RR:0.4 (0.1, 2.0)), and after 1 cycle of DET compared to 2 cycles of SET (RR:0.02-0.06) [37, 38, 40]. Findings were consistent regardless of maternal age (<33 or ≥33), duration of infertility (<3 or ≥3), or embryo grade (A or B).

#### Fresh versus FET

##### Pregnancy and miscarriages

In contrast to the results of an earlier review including only 1 RCT, the results of a recent pooled analysis of 3 RCTs found that clinical and ongoing pregnancy rates, but not miscarriage rates, were significantly higher after frozen compared to fresh IVF/ICSI cycles (RR:1.31, 1.32, respectively) [43, 46].

#### Stage of embryo during transfer

##### Cycle success

Two meta-analyses of 7 and 11 RCTs found that significantly fewer embryos were cryopreserved (41-53% vs. 63-71%; OR:0.3-0.5) and more women cancelled their cycle or failed to transfer any embryos (9% vs. 3-5%; OR:2.2-2.9) after blastocyst-stage embryo transfers than after cleavage-stage embryo transfers, even in studies where more cleavage-stage embryos were transferred [48, 50].

##### Pregnancy miscarriages and live births

In the same two meta-analyses, slightly higher clinical pregnancy rates were demonstrated after IVF/ICSI with blastocyst-stage embryos (39-40%) compared to cleavage-stage embryos (34-39%) [48, 50]. While these differences were significant in one (OR:1.3), the other only found significant differences in cumulative clinical pregnancy rates after all fresh and frozen IVF/ICSI cycles. Further, subgroup analyses revealed no differences when an equal number of cleavage and blastocyst stage embryos were transferred, or when more cleavage stage embryos than blastocyst stage embryos were transferred. No variation in the proportion of women experiencing miscarriages after blastocyst transfers versus cleavage transfers was found [50].

Further, these meta-analyses also demonstrated that IVF/ICSI with blastocyst-stage embryos was associated with a higher likelihood of a live birth than IVF/ICSI with cleavage-stage embryos (pooled OR: 1.4 (1.1, 1.8)) [48, 50]. When only women expected to have a good prognosis with blastocyst transfer were considered, differences were even greater, and, conversely, when unselected women or women with a poor prognosis were considered, no significant differences were shown.

##### Multiple pregnancies/births

No significant difference in the multiple pregnancy rates between blastocyst- and cleavage-stage embryo transfers were seen [48, 50].

#### Donor embryo transfer

##### Live births

No reviews discussed differences in effectiveness between donor and non-donor IVF. In a recent primary study of IVF/ICSI in 6 countries, in comparison to autologous IVF/ICSI (31%), live birth rates were slightly lower if donor embryos (28%) or frozen donor oocytes (27%) were used (RRs: 0.90 (0.87,0.93) and 0.90 (0.89,0.92), respectively), and significantly higher if fresh donor oocytes were used (47%; OR:1.50 (1.49,1.52)) [55].

##### Multiple pregnancies/births

In the same primary study, while no statistical comparisons were performed, multiple birth rates after fresh autologous IVF/ICSI were 17.2%, 30.3%, and 33.0% in Australia/New Zealand, Canada, and the US respectively, whereas multiple birth rates after IVF/ICSI with donor embryos were 16.1%, 24.9%, and 27.1% respectively [55].

## Discussion

This review summarizes the current state of the science around the safety and effectiveness of IVF/ICSI in comparison to spontaneous conception and less invasive ARTs and the impact of certain procedural factors on the safety and clinical effectiveness of IVF/ICSI for the treatment of infertility. This study was conducted to support policy development by the provincial government of Alberta. As public policy is usually context-dependent, the comparators chosen for this study were based on the ARTs interventions under consideration for funding in the province and the corresponding policy questions around the funding of these interventions. They did not include comparisons of the different drug regimens used or treatment ‘add-ons’, such as preimplantation genetic diagnosis or assisted hatching, as these considerations were not within the scope of the policy questions outlined by the provincial government.

In comparison to SC, IVF/ICSI appears to be associated with an increased risk of various complications occurring during pregnancy and delivery. It may also have adverse effects throughout infancy, childhood and adulthood. However, these trends could be due to increased surveillance in IVF/ICSI pregnancies, infertility itself, or maternal complications, and, the growth and development of the offspring still appears to follow normal patterns. Studies with longer follow-up are needed to confirm these findings.

Evidence compiled to date suggests that compared to SET, IVF/ICSI with DET is associated with more adverse events during pregnancy and delivery and safety issues in infants. However, embryo stage (blastocyst versus cleavage) does not appear to impact safety. Similarly, the health of infants born after donor cycles appears to be at least as good as that of infants born after autologous cycles. However, donor IVF is often associated with anovulation and advanced maternal and studies of donor cycles should take both of these factors into account. In comparison to fresh embryo transfer, the findings suggest that FET has fewer adverse events throughout pregnancy and delivery, and is least as safe as fresh embryo transfer in terms of infant outcomes.

Overall, IVF shows a clear benefit over no treatment and IUI for certain types of infertility with respect to pregnancy and live birth. Clinical pregnancy rates after IVF/ICSI do not appear to be influenced by the use of ICSI over IVF, although the differences in populations predicted to benefit from ICSI in comparison to IVF have not been thoroughly assessed. In fact, most of the studies comprising the review did not specifically address this point. The findings suggest that clinical pregnancy rates and live birth rates are similar or better after FET compared to fresh embryo transfer, and after blastocyst-stage embryo transfer compared to cleavage-stage embryo transfer, particularly in women considered to have a good prognosis. There appears to be little if any difference in the rate of multiple pregnancies between the two groups. DET cycles, rather than SET cycles, may greatly improve both pregnancy and live birth rates, but substantially increase the probability of a multiple pregnancy/birth. The same improvements in likelihood of pregnancy and live birth seen with DET and no increase in multiple birth rates appear to be achievable through 2 cycles of SET or 1 fresh SET cycle and 1 frozen SET cycle. Further, regardless of whether 1 or 2 embryos are implanted, IVF/ICSI with top quality embryos seems to yield better live birth rates than do less than top quality embryos.

## Conclusions

The safety of IVF/ICSI depends, in part on procedural choices made. However, these choices may reduce its effectiveness. Therefore, clinical and policy guidance need to ensure that the trade-offs involved are carefully considered by both patients and providers.

## Electronic supplementary material

Additional file 1: Table S1: Literature search. (DOC 302 KB)

Additional file 2: Table S2: Table of excluded papers. (DOC 52 KB)

Additional file 3: Table S3: Table of included reviews grouped by their primary comparison. (DOC 428 KB)

Additional file 4: Table S4: Table of included additional primary studies grouped by their primary comparison. (DOC 35 KB)

Additional file 5: Table S5: Quality of included reviews: Oxman & Guyatt index of scientific quality scoring system for systematic reviews. (DOC 69 KB)

Additional file 6: Table S6: Safety during pregnancy and delivery. (DOC 281 KB)

Additional file 7: Table S7: Neonatal/infant safety. (DOC 310 KB)

Additional file 8: Table S8: Effectiveness: number of oocytes retrieved. (DOC 29 KB)

Additional file 9: Table S9: Effectiveness: embryo cryopreservation rate. (DOC 43 KB)

Additional file 10: Table S10: Effectiveness: cycle cancellation rate. (DOC 50 KB)

Additional file 11: Table S11: Effectiveness: implantation rate. (DOC 36 KB)

Additional file 12: Table S12: Effectiveness: pregnancy rate. (DOC 100 KB)

Additional file 13: Table S13: Effectiveness: miscarriage rate. (DOC 52 KB)

Additional file 14: Table S14: Effectiveness: live birth rate. (DOC 120 KB)

Additional file 15: Table S15: Effectiveness: multiple pregnancy rate. (DOC 81 KB)

Additional file 16: Table S16: Effectiveness: multiple birth rate. (DOC 58 KB)

Below are the links to the authors’ original submitted files for images.

Authors’ original file for figure 1

Authors’ original file for figure 2

Authors’ original file for figure 3

## Abbreviations

ASD: Autism spectrum disorder
ART: Assisted reproductive technology
ARTs: Assisted reproductive technologies
BMI: Body mass index
CC: Clomiphene citrate
CI: Confidence interval
COS: Controlled ovarian stimulation
DET: Double embryo transfer
eSET: Elective single embryo transfer
FET: Frozen embryo transfer
FSH: Follicle-stimulating hormone
GnRH: Gonadotropin releasing hormone
GnRHa: Gonadotropin releasing hormone agonist
hMG: Human menopausal gonadotropin
ICSI: Intracytoplasmic sperm injection
IUI: Intrauterine insemination
IVF: In vitro fertilization
LBW: Low birth weight
MZT: Monozygotic twinning
NICU: Neonatal intensive care unit
OHSS: Ovarian hyperstimulation syndrome
OR: Odds ratio
PIH: Pregnancy induced hypertension
PRISMA: Preferred reporting items for systematic reviews and meta-analyses
PTB: Preterm birth
PTD: Preterm delivery
QET: Quadruple embryo transfer
RCT: Randomized controlled trial
RR: Risk ratio
SC: Spontaneous conception
SET: Single embryo transfer
sIUI: Stimulated intrauterine insemination
TET: Triple embryo transfer.
