# Supplementary material for: Assisted reproductive technologies (ARTs): Evaluation of evidence to support public policy development
Source: Reprod Health. 2014 Nov 7;11:76. doi: 10.1186/1742-4755-11-76 (PMC4233043; doi:10.1186/1742-4755-11-76)
Supplement: Supplementary file 1 — Additional file 1: Table S1: Literature search. (DOC 302 KB) [file 12978_2014_327_MOESM1_ESM.doc]

## Additional file 1: Table S1. Literature search.

| Part A – Search for systematic reviews | | |
| --- | --- | --- |
| **1. PubMed** ([www.pubmed.gov](http://www.pubmed.gov/)) | | |
| *Date of search*: Jan 17, 2012; updated monthly to April 06, 2013  *Limits/filters*: 2007 to date; English language; CADTH and MEDLINE systematic review filters (use of human & animal filters was attempted but these eliminated some potentially useful references) | | |
| [#38](http://www.ncbi.nlm.nih.gov/pubmed/advanced) | Search #33 OR #37 | [960](http://www.ncbi.nlm.nih.gov/pubmed/?cmd=HistorySearch&querykey=38) |
| [#37](http://www.ncbi.nlm.nih.gov/pubmed/advanced) | Search #34 OR #35 Limits: English, Publication Date from 2007 to 2012 | [524](http://www.ncbi.nlm.nih.gov/pubmed/?cmd=HistorySearch&querykey=37) |
| [#36](http://www.ncbi.nlm.nih.gov/pubmed/advanced) | Search #34 OR #35 | [548](http://www.ncbi.nlm.nih.gov/pubmed/?cmd=HistorySearch&querykey=36) |
| [#35](http://www.ncbi.nlm.nih.gov/pubmed/advanced) | Search #32 AND (systematic[sb] OR meta-analysis[pt] OR meta-analysis as topic[mh] OR meta analy*[tw] OR metanaly*[tw] OR metaanaly*[tw] OR met analy*[tw] OR integrative research[tiab] OR integrative review*[tiab] OR integrative overview*[tiab] OR research integration*[tiab] OR research overview*[tiab] OR collaborative review*[tiab] OR collaborative overview*[tiab] OR systematic review*[tiab] OR technology assessment*[tiab] OR "Technology Assessment, Biomedical"[mh] OR HTA[tiab] OR HTAs[tiab] OR "Cochrane Database Syst Rev"[Journal:__jrid21711] OR "health technology assessment winchester, england"[Journal] OR "Evid Rep Technol Assess (Full Rep)"[Journal] OR "Evid Rep Technol Assess (Summ)"[Journal] OR "Int J Technol Assess Health Care"[Journal] OR evidence based[tiab] OR best practice*[tiab] OR best evidence[tiab]) [CADTH systematic review filter] | [548](http://www.ncbi.nlm.nih.gov/pubmed/?cmd=HistorySearch&querykey=35) |
| [#34](http://www.ncbi.nlm.nih.gov/pubmed/advanced) | Search #32 AND systematic[sb] [MEDLINE systematic review filter] | [456](http://www.ncbi.nlm.nih.gov/pubmed/?cmd=HistorySearch&querykey=34) |
| [#33](http://www.ncbi.nlm.nih.gov/pubmed/advanced) | Search #32 AND (in process[sb] OR publisher[sb] OR pubmednotmedline[sb]) Limits: Publication Date from 2007 to 2012 | [459](http://www.ncbi.nlm.nih.gov/pubmed/?cmd=HistorySearch&querykey=33) |
| [#32](http://www.ncbi.nlm.nih.gov/pubmed/advanced) | Search #15 OR #16 OR #17 OR #18 OR #19 OR #20 OR #21 OR #22 OR #23 OR #25 OR #26 OR #27 OR #28 OR #29 OR #30 Limits: Publication Date from 2007 to 2012 | [11969](http://www.ncbi.nlm.nih.gov/pubmed/?cmd=HistorySearch&querykey=32) |
| [#31](http://www.ncbi.nlm.nih.gov/pubmed/advanced) | Search #15 OR #16 OR #17 OR #18 OR #19 OR #20 OR #21 OR #22 OR #23 OR #25 OR #26 OR #27 OR #28 OR #29 OR #30 | [58931](http://www.ncbi.nlm.nih.gov/pubmed/?cmd=HistorySearch&querykey=31) |
| [#30](http://www.ncbi.nlm.nih.gov/pubmed/advanced) | Search "embryo transfer*"[ti] | [2560](http://www.ncbi.nlm.nih.gov/pubmed/?cmd=HistorySearch&querykey=30) |
| [#29](http://www.ncbi.nlm.nih.gov/pubmed/advanced) | Search ICSI[ti] | [1091](http://www.ncbi.nlm.nih.gov/pubmed/?cmd=HistorySearch&querykey=29) |
| [#28](http://www.ncbi.nlm.nih.gov/pubmed/advanced) | Search intracytoplasmic[ti] | [2399](http://www.ncbi.nlm.nih.gov/pubmed/?cmd=HistorySearch&querykey=28) |
| [#27](http://www.ncbi.nlm.nih.gov/pubmed/advanced) | Search "assisted reproductive"[ti] OR "assisted reproduction"[ti] OR "assistive reproductive"[ti] OR "assistive reproduction"[ti] | [2570](http://www.ncbi.nlm.nih.gov/pubmed/?cmd=HistorySearch&querykey=27) |
| [#26](http://www.ncbi.nlm.nih.gov/pubmed/advanced) | Search "in vitro fertilisation"[ti] | [461](http://www.ncbi.nlm.nih.gov/pubmed/?cmd=HistorySearch&querykey=26) |
| [#25](http://www.ncbi.nlm.nih.gov/pubmed/advanced) | Search "in vitro fertilization"[ti] | [6304](http://www.ncbi.nlm.nih.gov/pubmed/?cmd=HistorySearch&querykey=25) |
| [#23](http://www.ncbi.nlm.nih.gov/pubmed/advanced) | Search IVF[ti] | [2816](http://www.ncbi.nlm.nih.gov/pubmed/?cmd=HistorySearch&querykey=23) |
| [#22](http://www.ncbi.nlm.nih.gov/pubmed/advanced) | Search infertility, male/th | [5165](http://www.ncbi.nlm.nih.gov/pubmed/?cmd=HistorySearch&querykey=22) |
| [#21](http://www.ncbi.nlm.nih.gov/pubmed/advanced) | Search infertility, female/th | [9639](http://www.ncbi.nlm.nih.gov/pubmed/?cmd=HistorySearch&querykey=21) |
| [#20](http://www.ncbi.nlm.nih.gov/pubmed/advanced) | Search infertility/th | [17344](http://www.ncbi.nlm.nih.gov/pubmed/?cmd=HistorySearch&querykey=20) |
| [#19](http://www.ncbi.nlm.nih.gov/pubmed/advanced) | Search sperm injections, intracytoplasmic[mh] | [3798](http://www.ncbi.nlm.nih.gov/pubmed/?cmd=HistorySearch&querykey=19) |
| [#18](http://www.ncbi.nlm.nih.gov/pubmed/advanced) | Search single embryo transfer[mh] | [73](http://www.ncbi.nlm.nih.gov/pubmed/?cmd=HistorySearch&querykey=18) |
| [#17](http://www.ncbi.nlm.nih.gov/pubmed/advanced) | Search embryo transfer[mh] | [11506](http://www.ncbi.nlm.nih.gov/pubmed/?cmd=HistorySearch&querykey=17) |
| [#16](http://www.ncbi.nlm.nih.gov/pubmed/advanced) | Search fertilization in vitro[mh] | [25039](http://www.ncbi.nlm.nih.gov/pubmed/?cmd=HistorySearch&querykey=16) |
| [#15](http://www.ncbi.nlm.nih.gov/pubmed/advanced) | Search reproductive techniques, assisted[mh] | [46922](http://www.ncbi.nlm.nih.gov/pubmed/?cmd=HistorySearch&querykey=15) |
| **2. The Cochrane Library** (<http://www.thecochranelibrary.com/>) | | |
| *Date of search*: Jan 17, 2012 (up to issue 12 of 12, 2011)  *Limits/filters*: 2007 to date; only Cochrane Database of Systematic Reviews, other reviews, technology assessments and economic evaluations used | | |
| #1 | [MeSH descriptor Reproductive Techniques, Assisted explode all trees](http://onlinelibrary.wiley.com/o/cochrane/searchHistory?mode=runquery&qnum=1) | 2484 |
| #2 | [MeSH descriptor Fertilization in Vitro explode all trees](http://onlinelibrary.wiley.com/o/cochrane/searchHistory?mode=runquery&qnum=2) | 1644 |
| #3 | [MeSH descriptor Embryo Transfer explode all trees](http://onlinelibrary.wiley.com/o/cochrane/searchHistory?mode=runquery&qnum=3) | 790 |
| #4 | [MeSH descriptor Single Embryo Transfer explode all trees](http://onlinelibrary.wiley.com/o/cochrane/searchHistory?mode=runquery&qnum=4) | 7 |
| #5 | [MeSH descriptor Sperm Injections, Intracytoplasmic explode all trees](http://onlinelibrary.wiley.com/o/cochrane/searchHistory?mode=runquery&qnum=5) | 387 |
| #6 | [MeSH descriptor Infertility explode all trees](http://onlinelibrary.wiley.com/o/cochrane/searchHistory?mode=runquery&qnum=6) | 1668 |
| #7 | [MeSH descriptor Infertility, Female explode all trees](http://onlinelibrary.wiley.com/o/cochrane/searchHistory?mode=runquery&qnum=7) | 905 |
| #8 | [MeSH descriptor Infertility, Male explode all trees](http://onlinelibrary.wiley.com/o/cochrane/searchHistory?mode=runquery&qnum=8) | 520 |
| #9 | [(IVF):ti or (in vitro fertilization):ti or (in vitro fertilisation):ti or (assisted reproductive):ti or (assisted reproduction):ti](http://onlinelibrary.wiley.com/o/cochrane/searchHistory?mode=runquery&qnum=9) | 2129 |
| #10 | [(assistive reproductive):ti or (assistive reproduction):ti or (intracytoplasmic):ti or (icsi):ti or (embryo transfer):ti](http://onlinelibrary.wiley.com/o/cochrane/searchHistory?mode=runquery&qnum=10) | 1116 |
| #11 | [(#1 OR #2 OR #3 OR #4 OR #5 OR #6 OR #7 OR #8 OR #9 OR #10), from 2007 to 2011](http://onlinelibrary.wiley.com/o/cochrane/searchHistory?mode=runquery&qnum=11) | 1072 |
|  | Cochrane Reviews [92] | [Other Reviews [97]](http://onlinelibrary.wiley.com/search-web/cochrane/mainSearch?mode=fromtab&ID=11&product=cldare&searchKey=b7729157-14e2-4423-9868-3e0b3ae4e316) [Technology Assessments [13]](http://onlinelibrary.wiley.com/search-web/cochrane/mainSearch?mode=fromtab&ID=11&product=clhta&searchKey=b7729157-14e2-4423-9868-3e0b3ae4e316) | [Economic Evaluations [35]](http://onlinelibrary.wiley.com/search-web/cochrane/mainSearch?mode=fromtab&ID=11&product=cleed&searchKey=b7729157-14e2-4423-9868-3e0b3ae4e316) | |
| **3. Centre for Reviews & Dissemination (DARE, HTA, NHS EED) databases** (<http://www.crd.york.ac.uk/crdweb/>) | | |
| *Date of search*: Jan 17, 2012  *Limits/filters*: 2007 to date | | |
| #1 | MeSH DESCRIPTOR Reproductive Techniques, Assisted EXPLODE ALL TREES | 258 |
| #2 | MeSH DESCRIPTOR Fertilization in Vitro EXPLODE ALL TREES | 173 |
| #3 | MeSH DESCRIPTOR Embryo Transfer EXPLODE ALL TREES | 55 |
| #4 | MeSH DESCRIPTOR undefined EXPLODE ALL TREES | 0 |
| #5 | MeSH DESCRIPTOR Sperm Injections, Intracytoplasmic EXPLODE ALL TREES | 48 |
| #6 | MeSH DESCRIPTOR Infertility EXPLODE ALL TREES | 156 |
| #7 | MeSH DESCRIPTOR Infertility, Female EXPLODE ALL TREES | 76 |
| #8 | MeSH DESCRIPTOR Infertility, Male EXPLODE ALL TREES | 40 |
| #9 | (IVF):TI OR (in vitro fertilization):TI OR (in vitro fertilisation):TI OR (assisted reproductive):TI OR (assisted reproductiion):TI | 165 |
| #10 | (assistive reproductive):TI OR (assistive reproduction):TI OR (intracytoplasmic):TI OR (icsi):TI OR (embryo transfer):TI | 72 |
| #11 | #1 OR #2 OR #3 OR #4 OR #5 OR #6 OR #7 OR #8 OR #9 OR #10 | 370 |
| #12 | * FROM 2007 TO 2012 | 22024 |
| #13 | #11 AND #12 | 168 |
| **4. EMBASE** (<http://www.ovid.com/webapp/wcs/stores/servlet/ProductDisplay?storeId=13051&catalogId=13151&langId=-1&partNumber=Prod-903>) | | |
| *Date of search*: Jan 17, 2012 (up to Ovid 2012 week 2)  *Limits/filters*: 2007 to date; English language; human; BMJ Clinical Evidence filter for systematic reviews | | |
| #1 | exp infertility therapy/ | 65184 |
| #2 | exp fertilization in vitro/ | 33251 |
| #3 | exp embryo transfer/ | 17046 |
| #4 | exp intracytoplasmic sperm injection/ | 9652 |
| #5 | exp infertility/th [Therapy] | 11167 |
| #6 | female infertility/th | 4663 |
| #7 | male infertility/th | 2509 |
| #8 | 1 or 2 or 3 or 4 or 5 or 6 or 7 | 67785 |
| #9 | exp review/ | 1750362 |
| #10 | (literature adj3 review$).ti,ab. | 166432 |
| #11 | exp meta analysis/ | 58505 |
| #12 | exp systematic review/ | 46604 |
| #13 | 9 or 10 or 11 or 12 | 1892520 |
| #14 | (medline or medlars or embase or pubmed or cinahl or amed or psychlit or psyclit or psychinfo or psycinfo or scisearch or cochrane).ti,ab. | 71610 |
| #15 | retracted article/ | 5390 |
| #16 | 14 or 15 | 76957 |
| #17 | 13 and 16 | 55480 |
| #18 | (systematic$ adj2 (review$ or overview)).ti,ab. | 43278 |
| #19 | (meta?anal$ or meta anal$ or meta-anal$ or metaanal$ or metaanal$).ti,ab. | 51153 |
| #20 | 17 or 18 or 19 | 112325 |
| #21 | 8 and 20 | 920 |
| #22 | limit 21 to (human and english language and yr="2007 -Current") | 452 |
| **5. MEDLINE** (Ovid MEDLINE In-Process & Other Non-Indexed Citations) | | |
| *Date of search*: Jan 17, 2012 (up to Ovid 2012 Week 02)  *Limits/filters*: 2007 to date; English language; human; BMJ Clinical Evidence filter for systematic reviews | | |
| #1 | exp Reproductive Techniques, Assisted/ | 47011 |
| #2 | exp Fertilization in Vitro/ | 25089 |
| #3 | exp Embryo Transfer/ | 11520 |
| #4 | exp Single Embryo Transfer/ | 74 |
| #5 | infertility/th | 2604 |
| #6 | infertility, female/th | 4567 |
| #7 | infertility, male/th | 2410 |
| #8 | exp Sperm Injections, Intracytoplasmic/ | 3826 |
| #9 | 1 or 2 or 3 or 4 or 5 or 6 or 7 or 8 | 50553 |
| #10 | (review or review, tutorial or review, academic).pt. | 1650735 |
| #11 | (medline or medlars or embase or pubmed or cochrane).tw,sh. | 60735 |
| #12 | (scisearch or psychinfo or psycinfo).tw,sh. | 6406 |
| #13 | (psychlit or psyclit).tw,sh. | 832 |
| #14 | cinahl.tw,sh. | 6204 |
| #15 | ((hand adj2 search$) or (manual$ adj2 search$)).tw,sh. | 5380 |
| #16 | (electronic database$ or bibliographic database$ or computer?ed database$ or online database$).tw,sh. | 7370 |
| #17 | (pooling or pooled or mantel haenszel).tw,sh. | 40680 |
| #18 | (peto or dersimonian or der simonian or fixed effect).tw,sh. | 2227 |
| #19 | (retraction of publication or retracted publication).pt. | 4112 |
| #20 | 11 or 12 or 13 or 14 or 15 or 16 or 17 or 18 or 19 | 108538 |
| #21 | 9 and 20 | 746 |
| #22 | meta-analysis.pt. | 31019 |
| #23 | meta-analysis.sh. | 31019 |
| #24 | (meta-analys$ or meta analys$ or metaanalys$).tw,sh. | 55704 |
| #25 | (systematic$ adj5 review$).tw,sh. | 36716 |
| #26 | (systematic$ adj5 overview$).tw,sh. | 647 |
| #27 | (quantitativ$ adj5 overview$).tw,sh. | 149 |
| #28 | (quantitativ$ adj5 review$).tw,sh. | 3454 |
| #29 | (quantitativ$ adj5 overview$).tw,sh. | 149 |
| #30 | (methodologic$ adj5 review$).tw,sh. | 2599 |
| #31 | (methodologic$ adj5 overview$).tw,sh. | 168 |
| #32 | (integrative research review$ or research integration).tw. | 77 |
| #33 | 22 or 23 or 24 or 25 or 26 or 27 or 28 or 29 or 30 or 31 or 32 | 85244 |
| #34 | 21 or 33 | 85704 |
| #35 | 9 and 34 | 991 |
| #36 | limit 35 to (english language and humans and yr="2007 -Current") | 372 |
| **6. CINAHL** (EBSCOhost) | | |
| *Date of search*: Jan 19, 2012  *Limits/filters*: 2007 to date; English language; CADTH systematic review filter | | |
| S4 | S1 and S2  Limiters - English Language; Published Date from: 20070101-20111231 | 118 |
| S3 | S1 and S2 | 153 |
| S2 | (MH meta analysis or MH systematic review or MH "Technology, Medical/EV" or PT systematic review or PT meta analysis or systematic* n3 review* or systematic* n3 overview* or methodologic* n3 review* or methodologic* n3 overview* or quantitative n3 review* or quantitative n3 overview* or quantitative n3 synthes* or research n3 integrati* or research n3 overview* or integrative n3 review* or integrative n3 overview* or collaborative n3 review* or collaborative n3 overview* or pool* n3 analy* or ti data synthes* or ab data synthes* or ti data extraction* or ab data extraction* or ti data abstraction* or ab data abstraction* or ti handsearch* or ab handsearch* or ti hand search* or ab hand search* or ti mantel haenszel or ab mantel haenszel or ti peto or ab peto or ti der simonian or ab der simonian or ti dersimonian or ab dersimonian or ti fixed effect* or ab fixed effect* or ti latin square* or ab latin square* or ti met analy* or ab met analy* or mw met analy* or ti metanaly* or ab metanaly* or metanaly* or ti health technology assessment* or ab health technology assessment* or ti hta or ab hta or ti htas or ab htas or ti meta regression* or ab meta regression* or ti metaregression* or ab metaregression* or ti mega regression* or ab mega regression* or mw systematic review* or mw biomedical technology assessment* or mw bio-medical technology assessment* or ti medline or ab medline or ti Cochrane or ab Cochrane or ti pubmed or ab pubmed or ti medlars or ab medlars or mw medline or mw Cochrane or mw pubmed or mw medlars or so Cochrane or so health technology assessment or so evidence report or cf y) | 56464 |
| S1 | MH reproduction techniques OR MH fertilization in vitro OR MH embryo transfer OR MH infertility/th | 4079 |
| **7. PsycINFO** (OvidSP) | | |
| *Date of search*: Jan 19, 2012 (up to 2012 week 2)  *Limits/filters*: 2007 to date; English language; human | | |
| #1 | exp Reproductive Technology/ | 712 |
| #2 | exp Infertility/ | 671 |
| #3 | 1 or 2 | 1213 |
| #4 | limit 3 to (human and english language and yr="2007 -Current") | 566 |
| #5 | exp "Literature Review"/ or review.mp. | 123414 |
| #6 | meta-analysis.mp. or exp Meta Analysis/ | 7137 |
| #7 | overview.mp. | 25531 |
| #8 | assessment.mp. or exp Measurement/ | 169792 |
| #9 | systematic.mp. | 22951 |
| #10 | exp Evaluation/ or evaluation.mp. | 85381 |
| #11 | 5 or 6 or 7 or 8 or 9 or 10 | 353575 |
| #12 | 4 and 11 | 125 |
| **8. Web of Science** (Thomson Reuters) | | |
| *Date of search*: Jan 20, 2012  *Limits/filters*: 2007 to date; English language | | |
| #4 | (HCI, CPCI-S, CPCI-SSH, BKCI-S, BKCI-SSH Timespan=2007-2012  Lemmatization=On | [97](http://apps.webofknowledge.com/summary.do?product=WOS&doc=1&qid=4&SID=2D8kHO2kiB196cDjHMh&search_mode=AdvancedSearch) |
| #3 | HCI, CPCI-S, CPCI-SSH, BKCI-S, BKCI-SSH Timespan=All Years  Lemmatization=On | [216](http://apps.webofknowledge.com/summary.do?product=WOS&doc=1&qid=3&SID=2D8kHO2kiB196cDjHMh&search_mode=CombineSearches) |
| #2 | Title=(review OR systematic) OR Title=("meta-analysis" OR "metaanalysis") OR Title=(assessment OR evaluation OR overview)  Databases=SCI-EXPANDED, SSCI, A&HCI, CPCI-S, CPCI-SSH, BKCI-S, BKCI-SSH Timespan=All Years  Lemmatization=On | [1,189,240](http://apps.webofknowledge.com/summary.do?product=WOS&doc=1&qid=2&SID=2D8kHO2kiB196cDjHMh&search_mode=GeneralSearch) |
| #1 | Title=("assisted reproductive" OR "assistive "reproductive" OR "assisted reproduction" OR "assistive reproduction") OR Title=(IVF OR "in vitro fertilization" OR "in vitro fertilisation") OR Title=("embryo transfer" OR intracytoplasmic" OR ICSI) OR Title=("infertility treatment*" OR "infertility therap*")  Databases=SCI-EXPANDED, SSCI, A&HCI, CPCI-S, CPCI-SSH, BKCI-S, BKCI-SSH Timespan=All Years  Lemmatization=On | [4,296](http://apps.webofknowledge.com/summary.do?product=WOS&doc=1&qid=1&SID=2D8kHO2kiB196cDjHMh&search_mode=GeneralSearch) |
| **9. Scopus** (SciVerse) | | |
| *Date of search*: Jan 20, 2012  *Limits/filters*: 2007 to date; English language | | |
|  | (TITLE("assisted reproduct*" OR ivf OR "in vitro fertilization" OR "in vitro fertilisation" OR intracytoplasmic OR icsi OR "infertility treatment*" OR "infertility therap*") AND TITLE(systematic OR review OR overview OR metaanalysis OR "meta-analysis" OR assessment OR evaluation)) AND PUBYEAR > 2006 | 277 |
| Part B – Search for primary studies | | |
| **1. PubMed** ([www.pubmed.gov](http://www.pubmed.gov/)) | | |
| *Date of search*: Mar 12, 2012; updated monthly to April 6, 2013  *Limits/filters*: 2008 to date; English language; human; study design | | |
| [#29](http://www.ncbi.nlm.nih.gov/pubmed) | Search #26 NOT (mice OR rats OR bovine OR buffalo OR beef OR cow* OR canine OR animal* OR pig OR pigs OR porcine OR camel OR cat OR cats) | [3785](http://www.ncbi.nlm.nih.gov/pubmed/?cmd=HistorySearch&querykey=29) |
| [#26](http://www.ncbi.nlm.nih.gov/pubmed) | Search #21 OR #24 | [4015](http://www.ncbi.nlm.nih.gov/pubmed/?cmd=HistorySearch&querykey=26) |
| [#24](http://www.ncbi.nlm.nih.gov/pubmed) | Search #22 NOT #3 Limits: Humans, English, Publication Date from 2008 to 2012 | [3521](http://www.ncbi.nlm.nih.gov/pubmed/?cmd=HistorySearch&querykey=24) |
| [#23](http://www.ncbi.nlm.nih.gov/pubmed) | Search #22 NOT #3 Limits: Publication Date from 2008 to 2012 | [5203](http://www.ncbi.nlm.nih.gov/pubmed/?cmd=HistorySearch&querykey=23) |
| [#22](http://www.ncbi.nlm.nih.gov/pubmed) | Search #20 AND (#1 OR #2) Limits: Publication Date from 2008 to 2012 | [5291](http://www.ncbi.nlm.nih.gov/pubmed/?cmd=HistorySearch&querykey=22) |
| [#21](http://www.ncbi.nlm.nih.gov/pubmed) | Search #20 AND (publisher[sb] OR in process[sb] OR pubmednotmedline[sb]) Limits: Publication Date from 2008 to 2012 | [494](http://www.ncbi.nlm.nih.gov/pubmed/?cmd=HistorySearch&querykey=21) |
| [#20](http://www.ncbi.nlm.nih.gov/pubmed) | Search #4 OR #5 OR #6 OR #7 OR #8 OR #9 OR #10 OR #11 OR #12 OR #13 OR #14 OR #15 OR #16 OR #17 OR #18 Limits: Publication Date from 2008 to 2012 | [10152](http://www.ncbi.nlm.nih.gov/pubmed/?cmd=HistorySearch&querykey=20) |
| [#19](http://www.ncbi.nlm.nih.gov/pubmed) | Search #4 OR #5 OR #6 OR #7 OR #8 OR #9 OR #10 OR #11 OR #12 OR #13 OR #14 OR #15 OR #16 OR #17 OR #18 | [59365](http://www.ncbi.nlm.nih.gov/pubmed/?cmd=HistorySearch&querykey=19) |
| [#18](http://www.ncbi.nlm.nih.gov/pubmed) | Search "embryo transfer*"[ti] | [2591](http://www.ncbi.nlm.nih.gov/pubmed/?cmd=HistorySearch&querykey=18) |
| [#17](http://www.ncbi.nlm.nih.gov/pubmed) | Search icsi[ti] | [1104](http://www.ncbi.nlm.nih.gov/pubmed/?cmd=HistorySearch&querykey=17) |
| [#16](http://www.ncbi.nlm.nih.gov/pubmed) | Search intracytoplasmic[ti] | [2417](http://www.ncbi.nlm.nih.gov/pubmed/?cmd=HistorySearch&querykey=16) |
| [#15](http://www.ncbi.nlm.nih.gov/pubmed) | Search "assisted reproductive"[ti] OR "assisted reproduction"[ti] OR "assistive reproductive"[ti] OR "assistive reproduction"[ti] | [2606](http://www.ncbi.nlm.nih.gov/pubmed/?cmd=HistorySearch&querykey=15) |
| [#14](http://www.ncbi.nlm.nih.gov/pubmed) | Search "in vitro fertilisation"[ti] | [462](http://www.ncbi.nlm.nih.gov/pubmed/?cmd=HistorySearch&querykey=14) |
| [#13](http://www.ncbi.nlm.nih.gov/pubmed) | Search "in vitro fertilization"[ti] | [6348](http://www.ncbi.nlm.nih.gov/pubmed/?cmd=HistorySearch&querykey=13) |
| [#12](http://www.ncbi.nlm.nih.gov/pubmed) | Search IVF[ti] | [2854](http://www.ncbi.nlm.nih.gov/pubmed/?cmd=HistorySearch&querykey=12) |
| [#11](http://www.ncbi.nlm.nih.gov/pubmed) | Search infertility, male/th | [5198](http://www.ncbi.nlm.nih.gov/pubmed/?cmd=HistorySearch&querykey=11) |
| [#10](http://www.ncbi.nlm.nih.gov/pubmed) | Search infertility,female/th | [9698](http://www.ncbi.nlm.nih.gov/pubmed/?cmd=HistorySearch&querykey=10) |
| [#9](http://www.ncbi.nlm.nih.gov/pubmed) | Search infertility/th | [17468](http://www.ncbi.nlm.nih.gov/pubmed/?cmd=HistorySearch&querykey=9) |
| [#8](http://www.ncbi.nlm.nih.gov/pubmed) | Search sperm injections, intracytoplasmic[mh] | [3848](http://www.ncbi.nlm.nih.gov/pubmed/?cmd=HistorySearch&querykey=8) |
| [#7](http://www.ncbi.nlm.nih.gov/pubmed) | Search single embryo transfer[mh] | [82](http://www.ncbi.nlm.nih.gov/pubmed/?cmd=HistorySearch&querykey=7) |
| [#6](http://www.ncbi.nlm.nih.gov/pubmed) | Search embryo transfer[mh] | [11566](http://www.ncbi.nlm.nih.gov/pubmed/?cmd=HistorySearch&querykey=6) |
| [#5](http://www.ncbi.nlm.nih.gov/pubmed) | Search fertilization in vitro[mh] | [25216](http://www.ncbi.nlm.nih.gov/pubmed/?cmd=HistorySearch&querykey=5) |
| [#4](http://www.ncbi.nlm.nih.gov/pubmed) | Search reproductive techniques, assisted[mh] | [47263](http://www.ncbi.nlm.nih.gov/pubmed/?cmd=HistorySearch&querykey=4) |
| [#3](http://www.ncbi.nlm.nih.gov/pubmed) | Search editorial[pt] OR comment[pt] OR letter[pt] OR newspaper article[pt] | [1172887](http://www.ncbi.nlm.nih.gov/pubmed/?cmd=HistorySearch&querykey=3) |
| [#2](http://www.ncbi.nlm.nih.gov/pubmed) | Search Controlled clinical trial[pt] OR controlled clinical trials as topic[mh] OR clinical trial[pt] OR clinical trials as topic[mh] OR "clinical trial"[tiab] OR evaluation studies[pt] OR evaluation studies as Topic[mh] OR control[tiab] OR controlled[tiab] OR volunteer[tiab] OR volunteers[tiab] OR open label*[tiab] OR nonrandom*[tiab] OR non random*[tiab] OR quasirandom*[tiab] OR Observational stud*[tiab] OR Cohort studies[Mesh] OR cohort[tiab] OR Longitudinal studies[Mesh] OR longitudinal[tiab] OR Prospective studies[Mesh] OR prospective[tiab] OR Follow-up studies[Mesh] OR follow up stud*[tiab] OR followup stud*[tiab] OR Retrospective studies[Mesh] OR retrospective[tiab] OR Population based stud*[tiab] OR Population based analy*[tiab] OR Population study[tiab] OR Population studies[tiab] OR descriptive stud*[tiab] OR Multidimensional stud*[tiab] OR "Comparative Study"[Publication Type] OR Comparative study[tiab] OR comparative studies[tiab] OR Case-control studies[Mesh] OR case control*[tiab] OR case series[tiab] OR case comparison*[tiab] OR Case history[tiab] OR Case histories[tiab] | [5163081](http://www.ncbi.nlm.nih.gov/pubmed/?cmd=HistorySearch&querykey=2) |
| [#1](http://www.ncbi.nlm.nih.gov/pubmed) | Search randomized controlled trial[pt] OR randomized controlled trials as topic[mh] OR random allocation [mh] OR double-blind method[mh] OR single-blind method[mh] OR random*[tw] OR "Placebos"[Mesh] OR placebo[tiab] OR ((singl*[tw] OR doubl*[tw] OR trebl*[tw] OR tripl*[tw]) AND (mask*[tw] OR blind*[tw] OR dumm*[tw])) | [834047](http://www.ncbi.nlm.nih.gov/pubmed/?cmd=HistorySearch&querykey=1) |
| Part C – Search for grey literature | | |
| Date of searches: Feb-Mar, 2012 | | |
| **1. ARTS-related association web sites** | | |
| Society of Obstetricians and Gynaecologists of Canada (SOGC) <http://www.sogc.org/index_e.asp> * scanned clinical practice guidelines, sections for reproductive endocrinology and infertility and SOGC policy and practice  Canadian Fertility and Andrology Society (CFAS) [www.cfas.ca/](http://www.cfas.ca/) *scanned Canadian ART Register annual reports, articles and reports sections | | |
| Cochrane Collaboration Menstrual Disorders & Subfertility Group [www.fmhs.auckland.ac.nz/som/obsgynae/research/cochrane/default.aspx](http://www.fmhs.auckland.ac.nz/som/obsgynae/research/cochrane/default.aspx) *scanned review topics list for female & male infertility | | |
| American Society for Reproductive Medicine (ASRM) [www.asrm.org/](http://www.asrm.org/) *scanned practice guidelines (series is called Committee Opinions – published in journal Fertility & Sterility) | | |
| Resolve: the National Infertility Association [www.resolve.org/](http://www.resolve.org/) *scanned sections of website | | |
| International Federation of Fertility Societies (IFFS) [www.iffs-reproduction.org/](http://www.iffs-reproduction.org/) *scanned sections of website | | |
| US Centers for Disease Control and Prevention (CDC) [www.cdc.gov/ART/index.htm](http://www.cdc.gov/ART/index.htm) *scanned sections of website | | |
| UK Human Fertilisation & Embryology Authority (HFEA) [www.hfea.gov.uk/](http://www.hfea.gov.uk/) *scanned website section on research and evidence | | |
| European Society for Human Reproduction (ESHRE) [www.eshre.eu/](http://www.eshre.eu/) *scanned guidelines and position papers sections of website (official journal is Human Reproduction) | | |
| Society for Assisted Reproductive Technology (SART) [www.sart.org/](http://www.sart.org/) *scanned website (official journal is Fertility & Sterility) | | |
| Assisted Human Reproduction Canada (AHRC) [www.ahrc-pac.gc.ca](http://www.ahrc-pac.gc.ca/) *scanned website, reports and publications | | |
| American Infertility Association (AFA) [www.ivf.net/ivf/the-american-infertility-association-o542.html](../../../../%5C%5Csphfs%5CHealth_Policy_and_Management%5CHPM%20Shared%20Folder%5CAssisted%20reproductive%20technologies%5CDraft%20report%5Cwww.ivf.net%5Civf%5Cthe-american-infertility-association-o542.html) *scanned website (mainly consumer information) | | |
| International Committee Monitoring Assisted Reproductive Technologies (ICMART) [www.icmartivf.org/](http://www.icmartivf.org/) *scanned publications (all from peer reviewed journals), reports, publications and presentations | | |
| Infertility Network [www.infertilitynetwork.org](http://www.infertilitynetwork.org/) *scanned website | | |
| **2. Guidelines** | | |
| National Guideline Clearinghouse [www.guidelines.gov](http://www.guidelines.gov/) *reproductive techniques, assisted or fertilization in vitro or embryo transfer or infertility or ivf or ICSI or intracytoplasmic = 83 references (1 relevant) | | |
| CMA Infobase [www.cma.ca/clinicalresources/practiceguidelines](http://www.cma.ca/clinicalresources/practiceguidelines) *icsi OR intracytoplasmic = 0 references; reproduction OR reproductive OR ivf OR infertility = 4 references (1 relevant, already identified) | | |
| Toward Optimum Practice (TOP guidelines) [www.topalbertadoctors.org/](http://www.topalbertadoctors.org/) *scanned guidelines under obstetrics & gynecology = 0 relevant references | | |
| Scottish Intercollegiate Guidelines Network (SIGN) [www.sign.ac.uk](http://www.sign.ac.uk/) *scanned guidelines under obstetrics & gynecology = 0 relevant references | | |
| Guidelines Advisory Committee (GAC) [www.gacguidelines.ca/index.cfm?pagepath=GAC_Endorsed_Guidelines&id=21080](http://www.gacguidelines.ca/index.cfm?pagepath=GAC_Endorsed_Guidelines&id=21080) *scanned section of website = 0 relevant references | | |
| New Zealand Guidelines Group [www.nzgg.org.nz/search?search=IVF](http://www.nzgg.org.nz/search?search=IVF) *infertility or IVF or assisted reproduction or ICSI or intracytoplasmic = 0 relevant references | | |
| BC Guidelines [www.bcguidelines.ca/alphabetical.html](http://www.bcguidelines.ca/alphabetical.html) *scanned alphabetic list of guidelines | | |
| Aetna Clinical Policy Bulletins [www.aetna.com/cpb](http://www.aetna.com/cpb) *infertility / IVF = 1 Clinical Policy Bulletin | | |
| **3. Clinical trials** | | |
| Clinical Trials.gov [www.clinicaltrials.gov](http://www.clinicaltrials.gov/) *reproductive techniques, assisted or fertilization in vitro or embryo transfer or infertility or ivf or ICSI or intracytoplasmic = 126 references | | |
| **4. HTA agency web sites** | | |
| *Canada*: | | |
| Canadian Agency for Drugs & Technologies in Health (CADTH) [www.cadth.ca](http://www.cadth.ca/) *"assisted OR assistive OR reproduction OR reproductive OR ARTS OR infertility OR fertility OR fertilization OR fertilisation OR ivf OR icsi OR intracytoplasmic" = 20 references (1 relevant, already identified) | | |
| Institut national d’excellence en santé et en services sociaux (INESSS) [www.inesss.qc.ca](http://www.inesss.qc.ca/) *scanned list of publications back to 2003 & projects in progress = 0 relevant references | | |
| Institute of Health Economics (IHE) [www.ihe.ca](http://www.ihe.ca/) *scanned publications back to 2007 = 1 relevant report (already identified) | | |
| McGill University Health Centre. Technology Assessment Unit [www.mcgill.ca/tau/](http://www.mcgill.ca/tau/) *scanned informal reports, work in progress and reports back to 2002 | | |
| Health Quality Ontario (HQO) (i.e., Ontario Medical Advisory Secretariat (MAS) / Ontario Health Technology Advisory Committee (OHTAC)) [www.hqontario.ca/en/mas/mas_ohtas_mn.html](http://www.hqontario.ca/en/mas/mas_ohtas_mn.html) *scanned reviews in progress, preliminary evidence reviews, OHTAC recommendations & evidence-based analyses = 1 relevant report & recommendation (already identified) | | |
| University of Calgary. Health Technology Assessment Unit <http://wcmprodlb.ucalgary.ca/cipph/HTAUnit> *scanned HTA projects list = 1 relevant report (already identified) | | |
| *UK*: | | |
| National Institute for Health and Clinical Excellence (NICE) [www.nice.org.uk](http://www.nice.org.uk/) *infertility = 59 reference (1 relevant, already identified) | | |
| *USA*: | | |
| Agency for Healthcare Research and Quality (AHRQ) [www.ahrq.gov](http://www.ahrq.gov/) *assisted reproductive = 30 references (1 relevant, already identified) | | |
| California Technology Assessment Forum (CTAF) [www.ctaf.org/](http://www.ctaf.org/) *scanned publications back to 2003 = 0 relevant references | | |
| **5. Other** | | |
| NHS Evidence [www.evidence.nhs.uk/nhs-evidence-content](http://www.evidence.nhs.uk/nhs-evidence-content) * "assisted reproductive" OR infertility OR IVF OR "in vitro fertili*" OR ICSI OR intracytoplasmic = 2,613 references (scanned first 100 hits only) | | |
| New York Academy of Medicine. Grey literature collection <http://greylit.org/> *(limited to 2007 – 2012) reproductive = 84 references; infertility = 1 reference; vitro = 0 references (0 relevant) | | |
| Open Grey: System for Information on Grey Literature in Europe [www.opengrey.eu/](http://www.opengrey.eu/) *assisted reproduct* = 16 references; infertility = 94 references (all older materials) | | |
| Google.ca [www.google.ca](http://www.google.ca/) ("assisted reproductive" OR IVF OR ICSI OR "infertility treatment*") AND (Alberta OR Canada OR guidelines OR standards) (scanned first 100 hits only) | | |
