# Supplementary material for: Assisted reproductive technologies (ARTs): Evaluation of evidence to support public policy development
Source: Reprod Health. 2014 Nov 7;11:76. doi: 10.1186/1742-4755-11-76 (PMC4233043; doi:10.1186/1742-4755-11-76)
Supplement: Supplementary file 2 — Additional file 2: Table S2: Table of excluded papers. (DOC 52 KB) [file 12978_2014_327_MOESM2_ESM.doc]

## Additional file 2: Table S2. Table of excluded papers.

| **Study** | **Main reason(s) for exclusion** |
| --- | --- |
| Allen et al. (2006). Pregnancy outcomes after assisted reproductive technology. | Publication date prior to 2007 |
| Bergh and Wennerholm (2012). Obstetric outcome and long-term follow up of children conceived through assisted reproduction. | Not a systematic review |
| Checa et al. (2009). IVF/ICSI with or without preimplantation genetic screening for aneuploidy in couples without genetic disorders: a systematic review and meta-analysis. | Assessment of treatment ‘add-on’ |
| Chuck and Yan (2009). Assistive reproductive technologies: a literature review and database analysis. | No useable outcomes (health technology assessment with no clinical review) |
| Clarke et al. (2010). Measuring outcomes in fertility trials: can we rely on clinical pregnancy rates? | IVF/ICSI could not be differentiated from other treatments |
| Das et al. (2009). Assisted hatching on assisted conception (IVF and ICSI). | Assessment of treatment ‘add-on’ |
| Derks et al. (2009). Techniques for preparation prior to embryo transfer. | Assessment of treatment ‘add-on’ |
| Dupont and Sifer (2012). A review of outcome data concerning children born following assisted reproductive technologies. | Not a systematic review |
| El-Toukhy et al. (2008). Outpatient hysteroscopy and subsequent IVF cycle outcome: a systematic review and meta-analysis. | Assessment of treatment ‘add-on’ |
| Elizur and Tulandi (2008). Drugs in infertility and fetal safety. | IVF/ICSI could not be differentiated from other treatments |
| Felix et al. (2009). International pooled analysis of cancer incidence in children after assisted reproductive technologies: interim report. | Not a systematic review |
| Hamoda et al. (2010). Outcome of fresh IVF/ICSI cycles in relation to the number of oocytes collected: A review of 4,701 treatment cycles. | Not a systematic review |
| Hansen et al. (2009). Twins born following assisted reproductive technology: perinatal outcome and admission to hospital. | Not a systematic review |
| Haute Autorite de Sante (HAS) (2006). Assessment of the indications and risks of ICSI (Intracytoplasmic sperm injection) to children born as a result of ICSI. | Publication date prior to 2007; full text article not available in English |
| Heijnen et al. (2008). Reduction of patient discomfort, risks and costs, but not pregnancies, by a mild strategy for in-vitro fertilisation. | Full text article not available in English |
| Hiller et al. (2010). Assisted reproductive technologies: a health technology assessment perspective. | Full text article not available in English |
| Hufnagel (2010). A literature review on the effect preimplantation genetic screening in combination with in vitro fertilization has on live birth rates in women over the age of 35 with a proposal for research to analyze the reasons behind the choice to elect for or aganist preimplantation genetic screening. | Assessment of treatment ‘add-on’ |
| Kyrou et al. (2009). How to improve the probability of pregnancy in poor responders undergoing in vitro fertilization: a systematic review and meta-analysis. | Comparison out of scope of review |
| Lee et al. (2007). Issues associated with assisted reproductive technologies (ARTS) and preimplantation genetic diagnosis (PGD): a rapid review conducted for the Alberta Health Technologies Decision Process. | Not a systematic review |
| Manipalviratn et al. (2009). Imprinting disorders and assisted reproductive technology. | Not a systematic review |
| Martins et al. (2011). Assisted hatching of human embryos: a systematic review and meta-analysis of randomized controlled trials. | Assessment of treatment ‘add-on’ |
| Medical Services Advisory Committee (MSAC) (2006). Surgical retrieval of sperm for intracytoplasmic sperm injection [part A] and Intracytoplasmic sperm injection using ejaculated sperm [part B]. | Publication date prior to 2007; comparison out of scope of review |
| Min et al. (2006). Guidelines for the number of embryos to transfer following in vitro fertilization. | Publication date prior to 2007; not a systematic review |
| Min et al. (2008). Guidelines for the number of embryos to transfer following in vitro fertilization. | Not a systematic review |
| Min et al. (2010). Elective single embryo transfer following in vitro fertilization. | Not a systematic review |
| Mukhopadhaya and Arulkumaran (2007). Reproductive outcomes after in-vitro fertilization. | Not a systematic review |
| Navarro et al. (2007). Assisted reproduction, multiple births and costs: an international comparison | Not a systematic review |
| Noble et al. (2010). Preimplantation genetic screening in advanced maternal age: a systematic review. | Assessment of treatment ‘add-on’ |
| Oudendijk (2012). The poor responder in IVF: is the prognosis always poor? A systematic review. | Comparison out of scope of review |
| The Practice Committee of the American Society for Reproductive Medicine and the Practice Committee of the Society for Assisted Reproductive Technology (2008). Ovarian tissue and oocyte cryopreservation. | Not a systematic review |
| The Practice Committee of the American Society for Reproductive Medicine (2008). Repetitive oocyte donation. | Not a systematic review |
| The Practice Committee of Society for Assisted Reproductive Technology and the Practice Committee of American Society for Reproductive Medicine. (2012). Elective single-embryo transfer. | Not a systematic review |
| The Practice Committee of the Society for Assisted Reproductive Technology and the Practice Committee of the American Society for Reproductive Medicine (2008). Preimplantation genetic testing: a Practice Committee opinion. | Not a systematic review |
| Rimm et al. (2011). A meta-analysis of the impact of IVF and ICSI on major malformations after adjusting for the effect of subfertility. | Not a systematic review |
| Saunders et al. (2011). Cerebral palsy and assisted conception. | Not a systematic review |
| The Scientific Advisory Committee of the Royal College of Obstetricians and Gynaecologists (2011). Multiple pregnancy following assisted reproduction. | Not a systematic review |
| Siristatidis et al. (2009). In vitro maturation in sub fertile women with polycystic ovarian syndrome undergoing assisted reproduction. | Assessment of treatment ‘add-on’ |
| Sunkara et al. (2010). The influence of delayed blastocyst formation on the outcome of frozen-thawed blastocyst transfer: a systematic review and meta-analysis. | Comparison out of scope of review |
| University of Calgary Centre for Health and Policy Studies (CHAPS) (2007). Assisted reproductive technologies. Draft final report. | No useable outcomes (health technology assessment – results of clinical review not reported in useable, systematic manner) |
| Van den Boogaard et al. (2012). The prognostic profile of subfertile couples and treatment outcome after expectant management, intrauterine insemination and in vitro fertilisation: a study protocol for the meta-analysis of individual patient data. | Not a systematic review (protocol only) |
| Van Peperstraten et al. (2010). Deciding how many embryos to transfer after in vitro fertilisation: development and pilot test of a decision aid. | Not a systematic review |
| Vulliemoz et al. (2012). In vitro fertilisation: perinatal risks and early childhood outcomes. | Not a systematic review |
| Wagenaar et al. (2008). An overview of studies on early development, cognition, and psychosocial well-being in children born after in vitro fertilization. | Not a systematic review |
| Williams et al. (2011). Systematic review and meta-analysis of cancer risk in children born after assisted reproduction. | Full text article not available in English |
| Woldringh et al. (2010). Karyotyping, congenital anomalies and follow-up of children after intracytoplasmic sperm injection with non-ejaculated sperm: a systematic review. | Comparison out of scope of review |
| Zreik et al. (2010). Fertility drugs and the risk of breast cancer: a meta-analysis and review. | IVF/ICSI could not be differentiated from other treatments |
