# Supplementary material for: Assisted reproductive technologies (ARTs): Evaluation of evidence to support public policy development
Source: Reprod Health. 2014 Nov 7;11:76. doi: 10.1186/1742-4755-11-76 (PMC4233043; doi:10.1186/1742-4755-11-76)
Supplement: Supplementary file 3 — Additional file 3: Table S3: Table of included reviews grouped by their primary comparison. (DOC 428 KB) [file 12978_2014_327_MOESM3_ESM.doc]

## Additional file 3: Table S3. Table of included reviews grouped by their primary comparison.

| **Review** | **Details of Review** | **Study Selection** | | **Characteristics of Included Studies** | | | **Outcomes** | |
| --- | --- | --- | --- | --- | --- | --- | --- | --- |
|  |  | **Inclusion criteria** | **Exclusion criteria** | **General** | **Population details** | **Procedure details** | **Pregnancy/delivery** | **Neonatal/infant** |
| **IVF/ICSI in comparison to non-invasive ART treatment options** | | | | | | | | |
| Thomopoulos et al. (2013) | *Country*: Greece  *Comparison*: impact of different ART modalities  *Date of literature search*: up to Dec 2010  *Meta-analysis*: no  *Sub-group or sensitivity analysis*: no | *Population(s)*: - |  | *Number of studies:* 47  • 1 RCT  • 33 case-control studies  • 4 prospective cohorts  • 9 retrospective cohorts  *Publication dates (range)*: 1992 - 2010  *Countries*: -  *Number of patients*: - | • *age*: -  • *BMI*: -  • *other*: - | • *ovarian stimulation protocol*: -  • n*umber of cycles*: -  • d*onor or autologous oocytes*: -  • f*rozen or fresh embryos*: -  • s*tage of embryo during transfer*: -  • *number of embryos transferred*: -  • q*uality of embryos transferred*: - | Primary:  *Effectiveness*: -  *Safety*:  • hypertensive complication rate  Secondary:  *Effectiveness*: -  *Safety*: - | Primary: -  Secondary: - |
|  |  | *Intervention(s)*:  • ART |  |  |  |  |  |  |
|  |  | *Comparator(s)*: - |  |  |  |  |  |  |
|  |  | *Outcome(s)*:  • hypertensive complications |  |  |  |  |  |  |
|  |  | *Design*: - | • reviews, case series, case reports, letters, editorials, commentaries, non-human studies |  |  |  |  |  |
|  |  | *Other*:  • all published studies providing evidence on the incidence of hypertensive complications in ART-initiated pregnancies | • non-English language |  |  |  |  |  |
| Pandian et al. (2011) | *Country*: Egypt and the UK  *Comparison*: IVF vs. expectant management, CC, and IUI ± ovarian stimulation  *Date of literature search*: up to Jul 2011  *Meta-analysis*: yes  *Sub-group or sensitivity analysis*: yes (treatment naïve vs. pretreated) | *Population(s)*: - |  | *Number of studies:* 6  • 6 RCTs  *Publication dates (range)*: 1993 - 2011  *Countries*: Canada (2), the Netherlands (3), the US (1)  *Number of patients*:  • IVF: 393  • expectant management: 41  • IUI alone: 54  • IUI + ovarian stimulation: 314 | • *age*: maternal upper age limit in 4 studies (range 38-40)  • *BMI*: -  • *other*: - | • *ovarian stimulation protocol*: varies slightly across studies reporting a protocol: GnRHa + hMG or FSH  • n*umber of cycles*: 1 cycle in 2 studies, up to 2 cycles in 1 study, up to 6 cycles in 2 studies (not specified in others)  • d*onor or autologous oocytes*: autologous oocytes only  • f*rozen or fresh embryos*: fresh cycles only in 2 studies, fresh and frozen cycles in 3 studies  • s*tage of embryo during transfer*: cleavage in 2 studies, cleavage or blastocyst in 2 studies (not specified in others)  • *number of embryos transferred*: 1-4  • q*uality of embryos transferred*: - | Primary:  *Effectiveness*:  • live birth rate  *Safety*: -  Secondary:  *Effectiveness*:  • pregnancy rate  • multiple pregnancy rate  *Safety*:  • OHSS rate | Primary: -  Secondary: - |
|  |  | *Intervention(s)*:  • IVF |  |  |  |  |  |  |
|  |  | *Comparator(s)*:  • expectant management  • CC  • IUI alone  • IUI with ovarian stimulation |  |  |  |  |  |  |
|  |  | *Outcome(s)*:  • live birth |  |  |  |  |  |  |
|  |  | *Design*:  • RCTs  • cross-over trials (if first phase could be extracted) |  |  |  |  |  |  |
|  |  | *Other*: - |  |  |  |  |  |  |
| **Number of embryos transferred** | | | | | | | | |
| Grady et al. (2012) | *Country*: Canada (Ontario)  *Comparison*: eSET vs. 1) DET or MET, or 2) spontaneous conception  *Date of literature search*: 1978/1980 - Mar 2011  *Meta-analysis*: yes  *Sub-group or sensitivity analysis*: no | *Population(s)*:  • single, twin, or multiple-order infants conceived by IVF/ICSI |  | *Number of studies:* 15  • 7* RCTs  • 1 quasi-RCT  • 4* prospective cohorts  • 4 retrospective cohorts  *1 study with 2 arms: 1 RCT and 1 prospective cohort  *Publication dates (range)*: 1999 - 2010  *Countries*: Australia (1), Belgium (4), Finland (3), France (1), the Netherlands (3), Saudi Arabia (1), Sweden (2)  *Number of patients*: 79,084  • eSET: 2,010  • DET: 2,175  • MET: 57  • SC: 74,842 | • *age*: maternal upper age limit in 7 RCTs (range 30-38) and 5 cohorts (range 36-39)  • *BMI*: lower and upper limit in 1 RCT (BMI 18-28)  • *other*: study population limited to women with a “good prognosis” (i.e., younger women in their 1^st^ or 2^nd^ IVF cycle with good embryo quality) in 5 RCTs | • *ovarian stimulation protocol*: varies slightly across RCTs: GnRHa + hMG or FSH in most, with 1 study using a GnRH antagonist in DET group; the same across all cohorts: GnRHa + FSH  • n*umber of cycles*: analysis included 1 cycle only  • d*onor or autologous oocytes*: autologous only in 5 RCTs (not specified in others)  • f*rozen or fresh embryos*: analysis included fresh cycles only  • s*tage of embryo during transfer*: cleavage in 4 RCTs and 5 cohorts, blastocyst in 1 cohort, cleavage or blastocyst in 1 RCT and 1 cohort (not specified in others)  • *number of embryos transferred*: 1 vs. 2 (primary comparison)  • q*uality of embryos transferred*: at least good quality in 6 RCTs and 1 cohort | Primary:  *Effectiveness*: -  *Safety*:  • preterm birth rate  Secondary:  *Effectiveness*:  • miscarriage rate  *Safety*:  • ectopic pregnancy rate  • preeclampsia rate  • placenta praevia rate  • placental abruption rate  • preterm prolonged rupture of membranes rate  • gestational diabetes rate  • early preterm birth rate  • caesarean section rate | Primary:  • low birth weight rate  • perinatal mortality rate  Secondary:  • very low birth weight rate  • mean birth weight  • mean gestational age at delivery  • proportion of infants small for gestational age  • proportion of infants with Apgar score <7  • NICU admission rate  • neonatal death rate  • congenital abnormalities |
|  |  | *Intervention(s)*:  • eSET |  |  |  |  |  |  |
|  |  | *Comparator(s)*:  • DET or MET  • spontaneous conception (SC) |  |  |  |  |  |  |
|  |  | *Outcome(s)*:  • perinatal mortality, preterm birth, birth weight |  |  |  |  |  |  |
|  |  | *Design*:  • randomized controlled trials  • cohort studies  • case-control studies | • editorials  • case reports  • letters |  |  |  |  |  |
|  |  | *Other*: | • duplicate publications  • studies with very small populations (N<10) |  |  |  |  |  |
| McLernon et al. (2010) | *Country*: Australia and several European (Belgium, Finland, the Netherlands, Sweden, the UK)  *Comparison*: eSET vs. DET  *Date of literature search*:  up to 2008  *Meta-analysis*: yes  (of individual patient data)  *Sub-group or sensitivity analysis*: yes  (by maternal age, embryo grade, and duration of infertility) | *Population(s)*:  • women undergoing autologous IVF/ICSI |  | *Number of studies:* 8  • all RCTs  *Publication dates (range)*: 1999 - 2008  *Countries*: Australia (1), Belgium (1), Finland (1), Netherlands (2), UK (1), multiple (2-Sweden, Denmark, Norway, the Netherlands)  *Number of patients*: 1,367  • eSET: 683  • DET: 684 | • *age*: maternal upper age limit in 6 studies (range 34-37); maternal age included in model*  • *BMI*: included in model*  • *other*: study population limited to women with a “good prognosis” (i.e., younger women in their 1^st^ or 2^nd^ IVF cycle with good embryo quality) in 6 studies; type, cause, and duration of infertility included in regression model*  *included as covariate in regression model to obtain adjusted OR (if found to be significant) | • *ovarian stimulation protocol*: varies slightly across studies reporting a protocol: GnRHa + hMG or FSH  • n*umber of cycles*: 1 cycle in 6 studies, 1-2 cycles in 2 studies; outcomes for more than 1 cycle analyzed separately  • d*onor or autologous oocytes*: autologous only in all studies  • f*rozen or fresh embryos*: analysis included fresh cycles only  • s*tage of embryo during transfer*: analysis included cleavage stage transfers only; day of transfer included in model*  • *number of embryos transferred*: 1 vs. 2 (primary comparison); number of embryos available for transfer included in model*  • q*uality of embryos transferred*: at least good quality in 7 studies  *included as covariate in regression model to obtain adjusted OR (if found to be significant) | Primary:  *Effectiveness*:  • live birth rate  • multiple birth rate  • cumulative live birth rate†  • cumulative multiple birth rate†  *Safety*: -  Secondary:  *Effectiveness*:  • miscarriage rate  • term singleton birth rate  *Safety*:  • preterm birth rate  • early preterm birth rate  • moderate preterm birth rate | Primary: -  Secondary: - |
|  |  | *Intervention(s)*:  • eSET with cleavage stage (day 2-3) embryos | • blastocyst (day 5) embryo transfer |  |  |  |  |  |
|  |  | *Comparator(s)*:  • DET with cleavage stage (day 2-3) embryos | • blastocyst (day 5) embryo transfer |  |  |  |  |  |
|  |  | *Outcome(s)*:  • live birth, multiple birth |  |  |  |  |  |  |
|  |  | *Design*:  • randomized controlled trials (including crossover trials)  • published and ongoing | • quasi-randomized controlled trials |  |  |  |  |  |
|  |  | *Other*:  • intervention differs only by number of embryos to be transferred (i.e., similar protocols for controlled ovarian stimulation, embryo culture, and embryo replacement) | • trials with other co-interventions |  |  |  |  |  |
| Gelbaya, Tsoumpou, and Nardo (2010) | *Country*: the UK  *Comparison*: eSET vs. DET  *Date of literature search*: Jan 1974 - Sep 2008  *Meta-analysis*: yes  *Sub-group or sensitivity analysis*: no | *Population(s)*:  • women undergoing IVF/ICSI |  | *Number of studies:* 6  • 5 RCTs  • 1 quasi-RCT  *Publication dates (range)*: 1999 - 2008  *Countries*: Belgium (1), Finland (1), the Netherlands (2), Saudi Arabia (1), multiple (1-Sweden, Denmark, Norway)  *Number of patients*: 1,354  • eSET: 678  • DET: 676 | • *age*: maternal upper age limit in 5 studies (range 30-36)  • *BMI*: -  • *other*: study population limited to women with a “good prognosis” (i.e., younger women in their 1^st^ or 2^nd^ IVF cycle with good embryo quality) in 4 studies | • *ovarian stimulation protocol*: varies slightly across studies: GnRHa + hMG or FSH  • n*umber of cycles*: analysis included 1 cycle only  • d*onor or autologous oocytes*: autologous only in 5 studies (not specified in others)  • f*rozen or fresh embryos*: analysis included fresh cycles only  • s*tage of embryo during transfer*: cleavage in 5 studies, majority cleavage in 1 study (98%)  • *number of embryos transferred*: 1 vs. 2 (primary comparison)  • q*uality of embryos transferred*: at least good quality in 5 studies | Primary:  *Effectiveness*:  • live birth rate  • multiple birth rate  *Safety*: -  Secondary:  *Effectiveness*:  • implantation rate  • pregnancy rate  • clinical pregnancy rate  • ongoing pregnancy rate  • miscarriage rate  *Safety*:  • ectopic pregnancy rate  • preterm delivery rate | Primary: -  Secondary: - |
|  |  | *Intervention(s)*:  • eSET | • blastocyst embryo transfer |  |  |  |  |  |
|  |  | *Comparator(s)*:  • DET | • blastocyst embryo transfer |  |  |  |  |  |
|  |  | *Outcome(s)*:  • live birth, multiple birth |  |  |  |  |  |  |
|  |  | *Design*:  • randomized controlled trials | • non-randomized studies |  |  |  |  |  |
|  |  | *Other*: - |  |  |  |  |  |  |
| Baruffi et al. (2009) | *Country*: Brazil  *Comparison*: SET vs. DET  *Date of literature search*: 1995 - 2008  *Meta-analysis*: yes  *Sub-group or sensitivity analysis*: yes (of studies that only included younger women) | *Population(s)*:  • women undergoing fresh IVF/ICSI |  | *Number of studies:* 7  • 6 RCTs  • 1 quasi-RCT  *Publication dates (range)*: 2001 - 2008  *Countries*: Belgium (1), Finland (1), the Netherlands (2), Saudi Arabia (1), the US (1), multiple (1-Sweden, Denmark, Norway)  *Number of patients*: 1,402  • SET: 701  • DET: 701 | • *age*: maternal upper age limit in 4 studies (range 30-35)  • *BMI*: -  • *other*: study population limited to women with a “good prognosis” (i.e., younger women in their 1^st^ or 2^nd^ IVF cycle with good embryo quality) in 5 studies | • *ovarian stimulation protocol*: varies slightly across studies: GnRHa + hMG and/or FSH  • n*umber of cycles*: 1 cycle in 5 studies, 1-2 cycles in 2 studies  • d*onor or autologous oocytes*: autologous only in 6 studies (not specified in others)  • f*rozen or fresh embryos*: analysis included fresh cycles only  • s*tage of embryo during transfer*: cleavage in 5 studies, majority cleavage in 1 study (98%), blastocyst in 1 study  • *number of embryos transferred*: 1 vs. 2 (primary comparison)  • q*uality of embryos transferred*: at least good quality in 5 studies | Primary:  *Effectiveness*:  • implantation rate  • ongoing pregnancy rate  • live birth rate  *Safety*: - | Primary: - |
|  |  | *Intervention(s)*:  • SET |  |  |  |  |  |  |
|  |  | *Comparator(s)*:  • DET |  |  |  |  |  |  |
|  |  | *Outcome(s)*:  • implantation, pregnancy, live birth |  |  |  |  |  |  |
|  |  | *Design*:  • randomized controlled trials  • published and ongoing |  |  |  |  |  |  |
|  |  | *Other*: - |  |  |  |  |  |  |
| Pandian et al. (2009) | *Country*: Egypt and the UK  *Comparison*: 1) DET vs. SET, TET, or QET, 2) TET vs. DET or QET  *Date of literature search*: 1970/1985 - 2008  *Meta-analysis*: yes  *Sub-group or sensitivity analysis*: no | *Population(s)*:  • subfertile women undergoing fresh, autologous or donor IVF/ICSI |  | *Number of studies:* 7  • all RCTs  *Publication dates (range)*: 1994 - 2006  *Countries*: Belgium (1), Finland (1), France (1), the Netherlands (3), multiple (1-Sweden, Denmark, Norway)  *Number of patients*: 1,374  • SET: 638  • DET: 686  • TET: 22  • QET: 28 | • *age*: maternal upper age limit in 4 studies (range 34-36); study population limited to older women (38-45) in 1 study  • *BMI*: -  • *other*: study population limited to women with a “good prognosis” (i.e., younger women in their 1^st^ or 2^nd^ IVF cycle with good embryo quality) in 4 studies | • *ovarian stimulation protocol*: varies slightly across studies: GnRHa + hMG and/or FSH  • n*umber of cycles*: 1 cycle in 5 studies, 1-2 cycles in 2 studies; outcomes for more than 1 cycle analyzed separately  • d*onor or autologous oocytes*: autologous only in 5 studies (not specified in others)  • f*rozen or fresh embryos*: analysis included fresh cycles only, except 1 study that reported outcomes after an additional frozen cycle ; outcomes for frozen cycle analyzed separately  • s*tage of embryo during transfer*: analysis included cleavage stage transfers only  • *number of embryos transferred*: 1, 2, 3, or 4 (primary comparison)  • q*uality of embryos transferred*: at least good quality in all studies | Primary:  *Effectiveness*:  • live birth rate  *Safety*: -  Secondary:  *Effectiveness*:  • clinical pregnancy rate  • multiple pregnancy/birth rate  • miscarriage rate  • cumulative live birth rate‡  *Safety*: - | Primary: -  Secondary: - |
|  |  | *Intervention(s)*:  • 1) DET  • 2) TET  • with cleavage stage embryos | • blastocyst embryo transfer |  |  |  |  |  |
|  |  | *Comparator(s)*:  • 1) SET, TET, or QET  • 2) DET or QET  • with cleavage stage embryos | • blastocyst embryo transfer |  |  |  |  |  |
|  |  | *Outcome(s)*:  • live birth |  |  |  |  |  |  |
|  |  | *Design*:  • randomized controlled trials (including crossover trials)  • published and ongoing |  |  |  |  |  |  |
|  |  | *Other*:  • standard procedures (i.e., standard protocols and materials for ovarian stimulation, ultrasound-guided oocyte retrieval, insemination, embryo culture, embryo replacement) |  |  |  |  |  |  |
| Van Loendersloot et al. (2010) | *Country*: the Netherlands  *Comparison*: association between factors pre-identified as potential predictors of success and pregnancy  *Date of literature search*: 1978 - Aug 2009  *Meta-analysis*: yes (some outcomes)  *Sub-group or sensitivity analysis*: no | *Population(s)*:  • subfertile women undergoing fresh, autologous IVF/ICSI | • studies including selected populations (subpopulations) only | *Number of studies:* 14  • 3 prospective cohorts  • 11 retrospective cohorts  *only 2 retrospective cohorts assessing the number of embryos transferred  *Publication dates (range)*: 1997 - 2008  *Countries*: Austria (1), Denmark (2), France (1), Hungary (1), the Netherlands (3), Sweden (1), the UK (3), the US (1), multiple (1; Australia and New Zealand)  *Number of patients*: 47,043 | • *age*: maternal upper age limit in 3 studies (38, 39, and 44); predictor in 13 studies  • *BMI*: predictor in 1 study  • *other*: indication for IVF is a predictor in 4 studies; type of subfertility is a predictor in 3 studies; duration of subfertility is a predictor in 3 studies; smoking is a predictor in 2 studies; basal FSH is a predictor in 7 studies | • *ovarian stimulation protocol*:  • n*umber of cycles*: 1 cycle in 7 studies; 1 or more cycles in 7 studies  • d*onor or autologous oocytes*: autologous only in all studies  • f*rozen or fresh embryos*: fresh cycles only in all studies  • s*tage of embryo during transfer*: cleavage embryos only in 5 studies, blastocyst embryos only in 1 study (not specified in others)  • *number of embryos transferred*: varies across studies (range 1-9); predictor in 2 studies (number of oocytes retrieved is a predictor in 7 studies)  • q*uality of embryos transferred*: predictor in 3 studies | Primary: -  *Effectiveness*:  • clinical pregnancy rate  • ongoing pregnancy rate  *Safety*: - | Primary: - |
|  |  | *Intervention(s)*:  • IVF/ICSI with ovarian stimulation (gonadotropins) protocol with down regulation |  |  |  |  |  |  |
|  |  | *Comparator(s)*:  • effect of pre-identified predictive maternal factors (maternal age, parity, basal FSH, duration of subfertility, indication for subfertility, number of oocytes retrieved, method of fertilization, number of embryos transferred, embryo quality) |  |  |  |  |  |  |
|  |  | *Outcome(s)*:  • pregnancy |  |  |  |  |  |  |
|  |  | *Design*: - |  |  |  |  |  |  |
|  |  | *Other*:  • - | • unconditional OR not reported and not enough data reported to calculate unconditional OR |  |  |  |  |  |
| **Fresh embryo transfer in comparison to frozen embryo transfer** | | | | | | | | |
| Roque et al. (2013) | *Country*: Spain and Brazil  *Comparison*: frozen ET vs. fresh ET  *Date of literature search*: up to Dec 2011  *Meta-analysis*: yes  *Sub-group or sensitivity analysis*: no | *Population(s)*:  • women undergoing IVF/ICSI |  | *Number of studies:* 3  • 3 RCTs  *Publication dates (range)*: 2010-2011  *Countries*: Iran (1), the US  *Number of patients*: 633  • FET: 317  • fresh ET: 316 | • *age*: 27-33 years  • *BMI*: -  • *other*: study population limited to high responders in 2 studies and normal responders in 1 study | • *ovarian stimulation protocol*: varies slightly across studies: GnRHa + FSH in 1, GnRH antagonist + FSH in 2  • n*umber of cycles*: 1 cycle per patient  • d*onor or autologous oocytes*: autologous oocytes only  • f*rozen or fresh embryos*: primary comparison  • s*tage of embryo during transfer*: almost all blastocyst in 2 studies, cleavage only in 1 study  • *number of embryos transferred*: mean of 2 in all studies  • q*uality of embryos transferred*: top quality in 1 study, best available in 2 studies | Primary:  *Effectiveness*:  • clinical pregnancy rate  • ongoing pregnancy rate  • miscarriage rate  *Safety*: -  Secondary:  *Effectiveness*: -  *Safety*: - | Primary: -  Secondary: - |
|  |  | *Intervention(s)*:  • frozen embryo transfer (FET) |  |  |  |  |  |  |
|  |  | *Comparator(s)*:  • fresh embryo transfer |  |  |  |  |  |  |
|  |  | *Outcome(s)*:  • pregnancy, miscarriage |  |  |  |  |  |  |
|  |  | *Design*:  • RCTs |  |  |  |  |  |  |
|  |  | *Other*: - |  |  |  |  |  |  |
| Maheshwari et al. (2012) | *Country*: the UK  *Comparison*: frozen ET vs. fresh ET  *Date of literature search*: 1984 - 2012  *Meta-analysis*: yes  *Sub-group or sensitivity analysis*: yes (matched vs. unmatched cohort) | *Population(s)*:  • singleton IVF/ICSI pregnancies |  | *Number of studies:* 11  • 4 matched cohorts  • 7 unmatched cohorts  *Publication dates (range)*: 1994 - 2011  *Countries*: -  *Number of patients*: - | • *age*: -  • *BMI*: -  • *other*: - | • *ovarian stimulation protocol*: -  • n*umber of cycles*: -  • d*onor or autologous oocytes*: -  • f*rozen or fresh embryos*: fresh vs. frozen (primary comparison)  • s*tage of embryo during transfer*: cleavage only in 3 studies, blastocyst only in 1 study, both in 3 studies (not specified in others)  • *number of embryos transferred*: -  • q*uality of embryos transferred*: not specified in most studies | *Effectiveness*: -  *Safety*:  • preterm birth rate  • early preterm birth rate  • caesarean section rate  • antepartum hemorrhage rate | • low birth weight rate  • very low birth weight rate  • proportion of infants small for gestational age  • congenital abnormalities  • perinatal mortality rate  • NICU admissions |
|  |  | *Intervention(s)*:  • frozen embryo transfer (FET) | • GIFT |  |  |  |  |  |
|  |  | *Comparator(s)*:  • fresh embryo transfer | • no comparator |  |  |  |  |  |
|  |  | *Outcome(s)*:  • obstetric or perinatal outcomes | • no obstetric or perinatal outcomes |  |  |  |  |  |
|  |  | *Design*:  • any observational study | • case reports, case series |  |  |  |  |  |
|  |  | *Other*:  • published | • not possible to differentiate outcomes for singletons and twins |  |  |  |  |  |
| Jee et al. (2009) | *Country*: South Korea  *Comparison*: frozen ET vs. fresh ET  *Date of literature search*: up to Feb 2008  *Meta-analysis*: yes  *Sub-group or sensitivity analysis*: yes (by stage of embryo transfer) | *Population(s)*:  • women undergoing autologous IVF |  | *Number of studies:* 7  • 1 single-arm trial  • 4 retrospective cohorts  • 1 case-control study  • 1 retrospective chart review  *Publication dates (range)*: 1994 - 2007  *Countries*: China (1), the US (6)  *Number of patients*: 13,059*  • FET: 2,125*  • fresh ET: 10,934*  *pregnancies | • *age*: no studies employed maternal age limits  • *BMI*: -  • *other*: unselected patients in all studies (i.e., all patients undergoing IVF within a certain time period at a particular clinic or in a particular region) | • *ovarian stimulation protocol*: varies slightly across studies reporting a protocol: GnRHa + hMG and/or FSH  • n*umber of cycles*: 1 or more per couple; actual number not specified in any study  • d*onor or autologous oocytes*: autologous only in all studies  • f*rozen or fresh embryos*: fresh vs. frozen (primary comparison)  • s*tage of embryo during transfer*: cleavage only in 3 studies, blastocyst only in 1 study, both in 3 studies  • *number of embryos transferred*: up to 4 in 2 studies, mean of 2 in 1 study (not specified in others)  • q*uality of embryos transferred*: varies widely across studies | Primary:  *Effectiveness*: -  *Safety*:  • ectopic pregnancy rate | Primary: - |
|  |  | *Intervention(s)*:  • frozen embryo transfer (FET) |  |  |  |  |  |  |
|  |  | *Comparator(s)*:  • fresh embryo transfer |  |  |  |  |  |  |
|  |  | *Outcome(s)*:  • ectopic pregnancy |  |  |  |  |  |  |
|  |  | *Design*: - | • registry reports |  |  |  |  |  |
|  |  | *Other*: *-* |  |  |  |  |  |  |
| Wennerholm et al. (2009)  • part I | *Country*: several European (Denmark, Finland, Norway, Sweden)  *Comparison*: frozen ET with cleavage stage embryos vs. 1) fresh ET, or 2) spontaneous conception  *Date of literature search*: 1984 - Sep 2008  *Meta-analysis*: no  *Sub-group or sensitivity analysis*: no | *Population(s)*:  • single or multiple-order infants conceived by IVF/ICSI | • single and multiple births not separated (except birth defect studies) | *Number of studies:* 25  • 1 RCT  • 12 retrospective cohorts  • 12 registry reports  *Publication dates (range)*: 1993 - 2008  *Countries*: Australia (2), Belgium (1), Denmark (1), India (1), Japan (1), Sweden (4), Turkey (1), the UK (3), the US (10), multiple European (1)  *Number of patients*: 170,874  • FET: 25,489  • fresh ET: 136,628  • SC: 8,757 | • *age*: -  • *BMI*: -  • *other*: - | • *ovarian stimulation protocol*: -  • n*umber of cycles*: -  • d*onor or autologous oocytes*: -  • f*rozen or fresh embryos*: fresh vs. frozen (primary comparison)  • s*tage of embryo during transfer*: -  • *number of embryos transferred*: -  • q*uality of embryos transferred*: - | *Effectiveness*: -  *Safety*:  • preterm birth rate | • low birth weight rate  • very low birth weight rate  • perinatal mortality rate  • birth defect rate  • chromosome aberration rate  • childhood morbidity  • growth pattern  • mental development |
|  |  | *Intervention(s)*:  • frozen embryo transfer (FET) with early cleavage stage embryos (cryopreservation by slow freezing or vitrification) | • donor and non-donor oocytes not separated |  |  |  |  |  |
|  |  | *Comparator(s)*:  • 1) fresh embryo transfer  • 2) spontaneous conception (SC)  • none for vitrification studies | • no control group (except vitrification) |  |  |  |  |  |
|  |  | *Outcome(s)*:  • neonatal/perinatal or child outcomes |  |  |  |  |  |  |
|  |  | *Design*:  • comparative | • non-comparative (except vitrification) |  |  |  |  |  |
|  |  | *Other*:  • English language |  |  |  |  |  |  |
| • part II | *Comparison*: frozen ET with blastocyst stage embryos or oocytes (no comparator) | *Population(s)*:  • single or multiple-order infants conceived by IVF/ICSI |  | *Number of studies:* blastocyst: 12  • 3 single-arm trials  • 1 retrospective analyses  • 8 case reports  oocyte: 30  • 1 non-randomized controlled trial  • 7 single-arm trials  • 4 retrospective analyses  • 2 case series  • 16 case reports  *Publication dates (range)*: blastocyst: 2000-2007  oocyte: 1999-2008  *Countries*:  blastocyst: Argentina (1), Japan (5), Korea (2), Spain (1), Sweden (1), the US (2)  oocyte: Argentina (2), Australia (2), Brazil (1), Canada (1), China (2), Germany (1), Hungary (1), Italy (10), Israel (1), Japan (3), Korea (1), Spain (1), Switzerland (1), Taiwan (2), the US (1)  *Number of patients*: - | • *age*: -  • *BMI*: -  • *other*: - | • *ovarian stimulation protocol*: -  • n*umber of cycles*: -  • d*onor or autologous oocytes*: -  • f*rozen or fresh embryos*: frozen cycles only  • s*tage of embryo during transfer*: -  • *number of embryos transferred*: -  • q*uality of embryos transferred*: - | *Effectiveness*: -  *Safety*:  • preterm delivery rate | • mean birth weight  • congenital malformations |
|  |  | *Intervention(s)*:  • frozen embryo transfer (FET) with blastocyst stage embryos or oocytes (cryopreservation by slow freezing or vitrification) | • in vitro maturation |  |  |  |  |  |
|  |  | *Comparator(s)*:  • none |  |  |  |  |  |  |
|  |  | *Outcome(s)*:  • neonatal/perinatal or child outcomes | • studies that do not report on the health of infants born |  |  |  |  |  |
|  |  | *Design*:  • any (including case reports) |  |  |  |  |  |  |
|  |  | *Other*:  • English language |  |  |  |  |  |  |
| D’Angelo and Amso (2007) | *Country*: the UK  *Comparison*: frozen ET vs. 1) fresh ET, or 2) intravenous albumin + fresh ET  *Date of literature search*: 1985 - May 2007  *Meta-analysis*: yes  *Sub-group or sensitivity analysis*: no | *Population(s)*:  • reproductive-age women undergoing IVF/ICSI with GnRHa pituitary down-regulation and superovulation | • women undergoing ovulation induction without | *Number of studies:* 2  • all RCTs  *Publication dates (range)*:  1) 1999  2) 1996  *Countries*:  1) Italy  2) the UK  *Number of patients*:  1) 125  • FET: 58  • fresh ET: 67  2) 26  • FET: 13  • IVA: 13 | • *age*: -  • *BMI*: 1) upper limit of 30  • *other*: - | • *ovarian stimulation protocol*: -  • n*umber of cycles*: 1 in both studies  • d*onor or autologous oocytes*: autologous in both studies  • f*rozen or fresh embryos*: 1) fresh vs. frozen; 2) IVA vs. frozen (primary comparison)  • s*tage of embryo during transfer*: 1) fresh ET: cleavage stage (FET: zygotes frozen immediately after fertilization); 2) IVA: blastocyst stage (FET: pronucleate stage embryos frozen)  • *number of embryos transferred*: 1) 3-4; 2) mean of 2  • q*uality of embryos transferred*: - | Primary:  *Effectiveness*:  • clinical pregnancy rate  *Safety*:  • incidence of moderate-severe OHSS  • incidence of nil-mild OHSS  Secondary:  *Effectiveness*:  • live birth rate  • mean number of oocytes retrieved  • mean number of oocytes fertilized  • mean number of embryo’s transferred  • mean resolution time (time to next menstrual period)  *Safety*:  • hospital admission rate | Primary: -  Secondary: - |
|  |  | *Intervention(s)*:  • frozen embryo transfer (FET) (cryopreservation of all embryos) |  |  |  |  |  |  |
|  |  | *Comparator(s)*:  • 1) fresh embryo transfer  • 2) intravenous albumin infusion (IVA) + fresh embryo transfer |  |  |  |  |  |  |
|  |  | *Outcome(s)*:  • OHSS, pregnancy |  |  |  |  |  |  |
|  |  | *Design*:  • randomized controlled trials | • crossover trials |  |  |  |  |  |
|  |  | *Other*: - |  |  |  |  |  |  |
| **Stage of embryo during transfer** | | | | | | | | |
| Chang et al. (2009) | *Country*: South Korea  *Comparison*: blastocyst stage ET vs. cleavage stage ET  *Date of literature search*: Jan 1995 - Nov 2007  *Meta-analysis*: yes  *Sub-group or sensitivity analysis*: yes (by publication year) | *Population(s)*:  • women undergoing and infants born after fresh, autologous IVF/ICSI |  | *Number of studies:*  sex ratio: 4*  • all retrospective cohorts  *an additional 4 unpublished studies included in a separate analysis (characteristics of these studies not provided)  MZT: 9  • 3 RCTs  • 1 prospective cohort  • 4 retrospective cohorts  • 1 case-control study  *Publication dates (range)*: 2001 - 2007  *Countries*:  sex ratio: Australia (1), the US (1)  MZT: Belgium (1), Brazil (1), Israel (2), the US (5)  *Number of patients*:  sex ratio: 2,487  • blastocyst: 1,102  • cleavage: 1,485  MZT: 40,917*  • blastocyst: 9,316  • cleavage: 31,601  *pregnancies | • *age*: maternal upper age limit in 5 studies (range 35-44; only 1 study at 44, the rest 35-37)  • *BMI*: -  • *other*: study population limited to couples with a “good prognosis” (i.e., those expected to do well with blastocyst culture) in 2 studies and to couples with a “poor prognosis” (i.e., those who had experienced multiple failures with conventional treatment or a poor response to ovulation induction) in 1 study; unselected patients in the other 10 studies | • *ovarian stimulation protocol*: varies slightly across studies reporting a protocol: GnRHa + hMG and/or FSH in most, with 1 study using a GnRH antagonist  • n*umber of cycles*: 1 or more per couple; actual number not specified in most studies  • d*onor or autologous oocytes*: analysis included autologous only  • f*rozen or fresh embryos*: analysis included fresh cycles only  • s*tage of embryo during transfer*: blastocyst vs. cleavage (primary comparison)  • *number of embryos transferred*: varies across studies (mean of 2-3 reported in 5 studies, max of 4 reported in 1 study)  • q*uality of embryos transferred*: varies widely across studies | *Effectiveness*: -  *Safety*: - | • sex ratio (number of male infants/number of female infants)  • monozygotic twinning rate |
|  |  | *Intervention(s)*:  • blastocyst embryo transfer |  |  |  |  |  |  |
|  |  | *Comparator(s)*:  • cleavage embryo transfer |  |  |  |  |  |  |
|  |  | *Outcome(s)*:  • sex ratio, monozygotic twinning (MZT) |  |  |  |  |  |  |
|  |  | *Design*:  • any comparative study |  |  |  |  |  |  |
|  |  | *Other*:  • published in peer-reviewed journal | • non-English language |  |  |  |  |  |
| Papanikolaou et al. (2008) | *Country*: Belgium  *Comparison*: blastocyst stage ET vs. cleavage stage ET  *Date of literature search*: 1966/1980 - Jul 2007  *Meta-analysis*: yes  *Sub-group or sensitivity analysis*: yes (including pseudo-randomized studies) | *Population(s)*:  • women undergoing IVF/ICSI |  | *Number of studies:* 8  • all RCTs  *Publication dates (range)*: 2000 - 2006  *Countries*: Belgium (5), Italy (1), Israel (1), Sweden (1)  *Number of patients*: 1,654  • blastocyst: 815  • cleavage: 839 | • *age*: maternal upper age limit in 6 studies (range 36-43; only 1 study at 43, the rest 36-41)  • *BMI*: upper limit of 30 in 1 study  • *other*: study population limited to couples with a “good prognosis” (i.e., those expected to do well with blastocyst culture) in 6 studies; unselected patients in the other 2 studies | • *ovarian stimulation protocol*: varies slightly across studies reporting a protocol: GnRHa + hMG and/or FSH in most, with 3 studies using a GnRH antagonist  • n*umber of cycles*: analysis included 1 cycle only  • d*onor or autologous oocytes*: autologous only in 1 study (not specified in others)  • f*rozen or fresh embryos*: analysis included fresh cycles only  • s*tage of embryo during transfer*: blastocyst vs. cleavage (primary comparison)  • *number of embryos transferred*: varies across studies (range 1-up to 3)  • q*uality of embryos transferred*: varies widely across studies | Primary:  *Effectiveness*:  • live birth rate  *Safety*: -  Secondary:  *Effectiveness*:  • clinical pregnancy rate  • multiple pregnancy rate  • cancellation rate  • cryopreservation rate  *Safety*: - | Primary: -  Secondary: - |
|  |  | *Intervention(s)*:  • blastocyst (day 5-6) embryo transfer |  |  |  |  |  |  |
|  |  | *Comparator(s)*:  • cleavage (day 2-3) embryo transfer |  |  |  |  |  |  |
|  |  | *Outcome(s)*: - |  |  |  |  |  |  |
|  |  | *Design*:  • randomized controlled trials (parallel only) | • pseudo-randomization methods |  |  |  |  |  |
|  |  | *Other*:  • equal number of embryos transferred between blastocyst and cleavage groups | • not published as full manuscript in peer-reviewed journal |  |  |  |  |  |
| Glujovsky et al. (2012) and Blake et al. (2007)§ | *Country*: Argenitina and New Zealand  *Comparison*: blastocyst stage ET vs. cleavage stage ET  *Date of literature search*: 1966/1980 - Feb 2012  *Meta-analysis*: yes  *Sub-group or sensitivity analysis*: yes (by distribution of embryos in blastocyst vs. cleavage groups, prognosis of study population, and time of randomization) | *Population(s)*:  • couples undergoing autologous or donor IVF/ICSI |  | *Number of studies:* 23  • all RCTs  *Publication dates (range)*: 1998 - 2011  *Countries*: Australia (1), Belgium (7), Brazil (1), Denmark (1), Egypt (1), France (1) Greece (1), Italy (2), Israel (3), Jordan (1), Sweden (1), the US (3)  *Number of patients*: 3,823*  *couples | • *age*: maternal upper age limit in 14 studies (range 35-43; only 1 study at 43, the rest 35-40)  • *BMI*: upper limit of 30 in 2 studies  • *other*: study population limited to couples with a “good prognosis” (i.e., those expected to do well with blastocyst culture) in 14 studies and to couples with a “poor prognosis” (i.e., those who had experienced multiple failures with conventional treatment or a poor response to ovulation induction) in 2 studies; unselected patients in the other 7 studies | • *ovarian stimulation protocol*: varies slightly across studies reporting a protocol: GnRHa + hMG and/or FSH in most, with 3 trials using a GnRH antagonist  • n*umber of cycles*: 1 or more per couple; actual number not specified in most  • d*onor or autologous oocytes*: autologous only in 2 studies (not specified in others)  • f*rozen or fresh embryos*: analysis included fresh cycles only  • s*tage of embryo during transfer*: blastocyst vs. cleavage (primary comparison)  • *number of embryos transferred*: varies across studies (range 1-up to 5)  • q*uality of embryos transferred*: varies widely across studies | Primary:  *Effectiveness*:  • live birth rate  *Safety*: -  Secondary:  *Effectiveness*:  • clinical pregnancy rate  • multiple pregnancy rate  • miscarriage rate  • cumulative pregnancy rate  • cryopreservation rate  • failure to transfer any embryos rate  *Safety*: - | Primary: -  Secondary: - |
|  |  | *Intervention(s)*:  • blastocyst (day 5-6) embryo transfer with single, sequential media culture | • in vitro maturation  • pre-implantation genetic diagnosis  • co-culture methods |  |  |  |  |  |
|  |  | *Comparator(s)*:  • cleavage (day 2-3) embryo transfer with single, sequential media culture |  |  |  |  |  |  |
|  |  | *Outcome(s)*:  • live birth, pregnancy, multiple pregnancy, embryo freezing, failure of embryo transfer |  |  |  |  |  |  |
|  |  | *Design*:  • randomized controlled trials | • quasi-randomized controlled trials |  |  |  |  |  |
|  |  | *Other*: - | • no data available from fresh cycle |  |  |  |  |  |
| Johnson et al. (2007)§ | *Country*: New Zealand  *Comparison*: blastocyst stage ET vs. cleavage stage ET  *Date of literature search*: 1966/1980 - May 2005  *Meta-analysis*: yes  *Sub-group or sensitivity analysis*: no | *Population(s)*:  • couples undergoing autologous or donor IVF/ICSI |  | *Number of studies:* 25  • 6 RCTs  • 19 non randomized controlled trials  *Publication dates (range)*: 2002 - 2004  *Countries*: RCTs: Australia (1), Belgium (2), Israel (1), Jordan (1), the US (1); details on other studies not reported  *Number of patients*: - | RCTs:  • *age*: maternal upper age limit in 5 studies (range 37-43; only 1 study at 43, the rest 37-39)  • *BMI*: -  • *other*: study population limited to couples with a “good prognosis” (i.e., those expected to do well with blastocyst culture) in 6 studies and to couples with a “poor prognosis” (i.e., those who had experienced multiple failures with conventional treatment or a poor response to ovulation induction) in 2 studies; unselected patients in the other studies | RCTs:  • *ovarian stimulation protocol*: varies slightly across studies reporting a protocol: GnRHa + hMG and/or FSH  • n*umber of cycles*: 1 or more per couple; actual number not specified in most  • d*onor or autologous oocytes*: autologous only in 1 study (not specified in others)  • f*rozen or fresh embryos*: analysis included fresh cycles only  • s*tage of embryo during transfer*: blastocyst vs. cleavage (primary comparison)  • *number of embryos transferred*: varies across studies (range 1-up to 4)  • q*uality of embryos transferred*: varies widely across studies | Primary:  *Effectiveness*: -  *Safety*: - | Primary:  • monozygotic twinning rate§ |
|  |  | *Intervention(s)*:  • blastocyst (day 5-6) embryo transfer |  |  |  |  |  |  |
|  |  | *Comparator(s)*:  • cleavage (day 2-3) embryo transfer |  |  |  |  |  |  |
|  |  | *Outcome(s)*:  • live birth, pregnancy, multiple pregnancy, miscarriage, embryo freezing, failure of embryo transfer, monozygotic twinning |  |  |  |  |  |  |
|  |  | *Design*:  • randomized controlled trials  • non-randomized studies for monozygotic twinning (if monozygotic twins considered separately from dizygotic twins) |  |  |  |  |  |  |
|  |  | *Other*: - |  |  |  |  |  |  |
| **Embryo donation** | | | | | | | | |
| Van der Hoorn et al. (2010) | *Country*: the Netherlands and  the US  *Comparison*: embryo donation (no comparator)  *Date of literature search*: up to Jan 2010  *Meta-analysis*: no  *Sub-group or sensitivity analysis*: no | *Population(s)*: - |  | *Number of studies:* 79  *Publication dates (range)*: -  *Countries*: -  *Number of patients*: - | • *age*: -  • *BMI*: -  • *other*: - | • *ovarian stimulation protocol*: -  • n*umber of cycles*: -  • d*onor or autologous oocytes*: donor  • f*rozen or fresh embryos*: -  • s*tage of embryo during transfer*: -  • *number of embryos transferred*: -  • q*uality of embryos transferred*: - | *Effectiveness*: -  *Safety*:  • maternal/pregnancy complication rate  • caesarean section rate  • pregnancy-induced hypertension rate  • first or second trimester bleeding rate  • placental pathology | • fetal/neonatal complication rate |
|  |  | *Intervention(s)*:  • embryo donation |  |  |  |  |  |  |
|  |  | *Comparator(s)*: - |  |  |  |  |  |  |
|  |  | *Outcome(s)*:  • pregnancy, pregnancy complications, placental pathology, immunologic aspects | • exclusive focus on ethics of egg donation |  |  |  |  |  |
|  |  | *Design*:  • original  • review | • case reports, letters |  |  |  |  |  |
|  |  | *Other*: - |  |  |  |  |  |  |
| **IVF/ICSI in comparison to spontaneous conception** | | | | | | | | |
| Hansen et al. (2013) | *Country*: Australia and the UK  *Comparison*: IVF/ICSI vs. spontaneous conception  *Date of literature search*: 1978 - Sep 2012  *Meta-analysis*: yes  *Sub-group or sensitivity analysis*: yes (sample size, setting, defect classification, quality, region, adjusted vs. non-adjusted, singles vs. multiples, major vs. minor defects, IVF vs. ICSI) | *Population(s)*:  • infants conceived spontaneously or by IVF/ICSI |  | *Number of studies:* 45  • all cohort studies  *Publication dates (range)*: 1995 – 2012  *Countries*: Australia (6), Austria (1), Belgium (3), Canada (3), Denmark (3), Egypt (1), Finland (3), France (1), Germany (2), Greece (1), Hungary (1), Ireland (1), Israel (2), Italy (1), Japan (2), Lebanon (2), the Netherlands (3), South Korea (1), Sweden (2), Taiwan (1), Turkey (1), the UK (1), the US (3)  *Number of patients*:  • IVF/ICSI: 92,671  • SC: 3,870,760 | • *age*: -  • *BMI*: -  • *other*: - | • *ovarian stimulation protocol*: -  • n*umber of cycles*: -  • d*onor or autologous oocytes*: -  • f*rozen or fresh embryos*: -  • s*tage of embryo during transfer*: -  • *number of embryos transferred*: -  • q*uality of embryos transferred*: - | Primary:  *Effectiveness*: -  *Safety*: - | Primary:  • birth defect risk |
|  |  | *Intervention(s)*:  • IVF/ICSI, GIFT, ZIFT | • OI or IUI or mixed exposure group |  |  |  |  |  |
|  |  | *Comparator(s)*:  • spontaneous conception (SC) | • studies comparing one ART technique to another |  |  |  |  |  |
|  |  | *Outcome(s)*:  • birth defects | • studies looking at a single type or group of birth defects |  |  |  |  |  |
|  |  | *Design*:  • - | • cross-sectional |  |  |  |  |  |
|  |  | *Other*:  • - | • non-English  • studies published as abstracts only  • overlapping data |  |  |  |  |  |
| Hart and Norman (2013) a and b | *Country*: Australia  *Comparison*:  *Date of literature search*: Jan 2000 - Apr 2012  *Meta-analysis*: no  *Sub-group or sensitivity analysis*: no | *Population(s)*:  • children conceived by ART | • studies limited to multiples | *Number of studies:* -  *Publication dates (range)*: 2000 - 2011  *Countries*: -  *Number of patients*: - | • *age*: -  • *BMI*: -  • *other*: - | • *ovarian stimulation protocol*: -  • n*umber of cycles*: -  • d*onor or autologous oocytes*: autologous only  • f*rozen or fresh embryos*: both fresh and frozen included  • s*tage of embryo during transfer*: -  • *number of embryos transferred*: -  • q*uality of embryos transferred*: - | Primary:  *Effectiveness*: -  *Safety*: - | Primary:  • physical health outcomes beyond first year of life (a)  • psychosocial health outcomes beyond first year of life (b) |
|  |  | *Intervention(s)*:  • ART (IVF/ICSI, GIFT, ZIFT, tubal embryo transfer) | • PGD/PGS, IVM, surrogacy, donor oocytes, donor sperm  • not possible to identify treatment |  |  |  |  |  |
|  |  | *Comparator(s)*:  • general population  • spontaneous conception (SC) | • no control group |  |  |  |  |  |
|  |  | *Outcome(s)*:  • physical health outcomes beyond first year of life (a)  • psychosocial health outcomes beyond first year of life (b) | • follow-up <12 months |  |  |  |  |  |
|  |  | *Design*:  • case series  • data linkage studies  • prospective studies |  |  |  |  |  |  |
|  |  | *Other*:  • English  • published in peer-reviewed journal | • non-English  • <70 children in either group |  |  |  |  |  |
| Pinborg et al. (2013) | *Country*: Denmark, Finland, Norway, and Sweden  *Comparison*: assess explanatory factors for adverse perinatal outcomes in ART singletons‡‡  *Date of literature search*: Jan 1982 - Apr 2012  *Meta-analysis*: yes  *Sub-group or sensitivity analysis*: yes | *Population(s)*:  • singletons conceived by ART |  | *Number of studies:* 65  • all cohort or registry studies  *Publication dates (range)*: 1993 - 2012  *Countries*: -  *Number of patients*: - | • *age*: -  • *BMI*: -  • *other*: - | • *ovarian stimulation protocol*: -  • n*umber of cycles*: -  • d*onor or autologous oocytes*: autologous oocytes only  • f*rozen or fresh embryos*: fresh cycles only  • s*tage of embryo during transfer*: -  • *number of embryos transferred*: -  • q*uality of embryos transferred*: - | Primary:  *Effectiveness*: -  *Safety*:  • preterm birth rate  Secondary:  *Effectiveness*: -  *Safety*:  • very preterm birth rate | Primary: -  Secondary:  • low birth weight rate  • very low birth weight rate  • proportion of infants small for gestational age  • perinatal mortality rate |
|  |  | *Intervention(s)*:  • ART (ovulation induction, IUI, IVF/ICSI, GIFT, ZIFT) | • donor oocyte cycles  • frozen oocyte cycles |  |  |  |  |  |
|  |  | *Comparator(s)*:  • | • no control group |  |  |  |  |  |
|  |  | *Outcome(s)*:  • preterm birth |  |  |  |  |  |  |
|  |  | *Design*:  • original studies |  |  |  |  |  |  |
|  |  | *Other*:  • English language | • <100 children  • studies published only as abstracts |  |  |  |  |  |
| Pandey et al. (2012) | *Country*: the UK  *Comparison*: IVF/ICSI vs. spontaneous conception  *Date of literature search*: 1978 - 2012  *Meta-analysis*: yes  *Sub-group or sensitivity analysis*: yes (matched vs. unmatched cohort) | *Population(s)*:  • singleton IVF/ICSI pregnancies |  | *Number of studies:* 30  • 20 matched cohorts  • 10 unmatched cohorts  *Publication dates (range)*: 1994 - 2011  *Countries*: -  *Number of patients*: - | • *age*: -  • *BMI*: -  • *other*: - | • *ovarian stimulation protocol*: -  • n*umber of cycles*: -  • d*onor or autologous oocytes*: -  • f*rozen or fresh embryos*: -  • s*tage of embryo during transfer*: -  • *number of embryos transferred*: -  • q*uality of embryos transferred*: - | *Effectiveness*: -  *Safety*:  • preterm birth rate  • early preterm birth rate  • caesarean section rate  • antepartum hemorrhage rate  • hypertensive complication rate  • PPROM  • gestational diabetes | • low birth weight rate  • very low birth weight rate  • proportion of infants small for gestational age  • congenital abnormalities  • perinatal mortality rate  • NICU admissions |
|  |  | *Intervention(s)*:  • IVF/ICSI | • GIFT |  |  |  |  |  |
|  |  | *Comparator(s)*:  • spontaneous conception (SC) | • no SC comparator  • 2 different aspects of IVF/ICSI compared |  |  |  |  |  |
|  |  | *Outcome(s)*:  • obstetric or perinatal outcomes | • no obstetric or perinatal outcomes |  |  |  |  |  |
|  |  | *Design*:  • any observational study | • case reports, case series |  |  |  |  |  |
|  |  | *Other*:  • published | • not possible to differentiate outcomes for singletons and twins |  |  |  |  |  |
| Wen et al. (2012) | *Country*: China  *Comparison*: 1) IVF/ICSI vs. spontaneous conception, or 2) IVF vs. ICSI  *Date of literature search*: up to Sep 2011  *Meta-analysis*: yes  *Sub-group or sensitivity analysis*: no | *Population(s)*:  • children conceived by IVF/ICSI |  | *Number of studies:* 56  *Publication dates (range)*: 1989 - 2011  *Countries*: -  *Number of patients*: - | • *age*: -  • *BMI*: -  • *other*: 36 studies controlled for potential confounding factors (e.g., maternal age, parity, sex, smoking, …) | • *ovarian stimulation protocol*: -  • n*umber of cycles*: -  • d*onor or autologous oocytes*: -  • f*rozen or fresh embryos*: -  • s*tage of embryo during transfer*: -  • *number of embryos transferred*: -  • q*uality of embryos transferred*: - | *Effectiveness*: -  *Safety*: - | • birth defects |
|  |  | *Intervention(s)*:  • IVF/ICSI |  |  |  |  |  |  |
|  |  | *Comparator(s)*:  • 1) spontaneous conception (SC)  • 2) ICSI |  |  |  |  |  |  |
|  |  | *Outcome(s)*:  • birth defects | • no control group  • inappropriate control group |  |  |  |  |  |
|  |  | *Design*:  • - | • case reports |  |  |  |  |  |
|  |  | *Other*:  • English language  • risk ratio and 95%CI directly stated or calculable from reported study data | • not published as full report |  |  |  |  |  |
| Rossi and D’Addario (2011) | *Country*: Italy  *Comparison*: ART vs. spontaneous conception  *Date of literature search*: Jan 2000 - Sep 2010  *Meta-analysis*: yes  *Sub-group or sensitivity analysis*: yes (unlike sex twins) | *Population(s)*:  • twins delivered at >21 gestational weeks or >500 grams conceived by ART | • singletons  • twins reduced to singleton (in utero multifetal reduction) | *Number of studies:* 13  • 2 prospective cohorts  • 11 retrospective cohorts  *Publication dates (range)*: 2000 - 2009  *Countries*: Australia (1), Austria (1), Belgium (2), Germany (1), Greece (1), Hungary (1), Israel (1), Lebanon (1), the Netherlands (1), Turkey (1), the United Arab Emirates (1), the US (1)  *Number of patients*: 23,031  • ART: 7,506  • SC: 15,525 | • *age*: maternal age controlled for in 5 studies  • *BMI*: -  • *other*: parity controlled for in 5 studies; 1 study did not report which variables were taken into account; unselected patients in all studies (i.e., all twin pregnancies/deliveries within a certain time period at a particular clinic or in a particular region) | • *ovarian stimulation protocol*: -  • n*umber of cycles*: -  • d*onor or autologous oocytes*: -  • f*rozen or fresh embryos*: -  • s*tage of embryo during transfer*: -  • *number of embryos transferred*: -  • q*uality of embryos transferred*: - | *Effectiveness*: -  *Safety*: -  • preterm birth rate  • moderate preterm birth rate  • caesarean section rate (vaginal delivery rate) | • normal birth weight rate  • low birth weight rate  • very low birth weight rate  • NICU admission rate  • perinatal death rate  • birth defect rate |
|  |  | *Intervention(s)*:  • ART¶ |  |  |  |  |  |  |
|  |  | *Comparator(s)*:  • spontaneous conception (SC) |  |  |  |  |  |  |
|  |  | *Outcome(s)*:  • neonatal outcomes  • reported per twins, proportional rates | • reported per pregnancy |  |  |  |  |  |
|  |  | *Design*:  • - | • letters, personal communications |  |  |  |  |  |
|  |  | *Other*:  • - | • non-English language |  |  |  |  |  |
| Wilson et al. (2011) | *Country*: Australia  *Comparison*: ART vs. spontaneous conception  *Date of literature search*: Jan 1998 - Oct 2010  *Meta-analysis*: no  *Sub-group or sensitivity analysis*: no | *Population(s)*:  • individuals of adolescent or older age (mean sample age of ≥12 years or mean follow-up period of ≥12 years) conceived by ART | • individuals of younger age or studies with a mean follow-up of <12yrs | *Number of studies:* 17*  • 14 retrospective cohorts  • 3 cross-sectional studies  *represent only 9 different populations – 7 publications on 1 Netherlands cohort, 3 publications on 1 UK cohort)  *Publication dates (range)*: 2001 - 2010  *Countries*: Belgium (2), Germany (1), Netherlands (7*), Sweden (1), the UK (3*), the US (2), multiple (1-several European)  *Number of patients*: - | • *age*: majority of children school-aged (range across studies: 0-24)  • *BMI*: -  • *other*: - | • *ovarian stimulation protocol*: -  • n*umber of cycles*: -  • d*onor or autologous oocytes*: inclusion of donor oocytes reported in 2 studies  • f*rozen or fresh embryos*: -  • s*tage of embryo during transfer*: -  • *number of embryos transferred*: -  • q*uality of embryos transferred*: - | *Effectiveness*: -  *Safety*: - | • growth and metabolism  • general physical health  • childhood cancer  • cognitive development  • psychological adjustment and socio-emotional functioning  • parent-adolescent relationship |
|  |  | *Intervention(s)*:  • ART (IVF, GIFT, ZIFT, tubal ET, gamete or embryo cryopreservation, oocyte or embryo donation, gestational surrogacy) | • artificial insemination  • ART exposure not made explicit |  |  |  |  |  |
|  |  | *Comparator(s)*: - |  |  |  |  |  |  |
|  |  | *Outcome(s)*:  • physical health and development, psychosocial health and adjustment |  |  |  |  |  |  |
|  |  | *Design*:  • - | • clinical case studies |  |  |  |  |  |
|  |  | *Other*:  • English language  • published in peer-reviewed journal |  |  |  |  |  |  |
| McDonald et al. (2010) | *Country*: Canada (Ontario)  *Comparison*: IVF/ICSI vs. spontaneous conception  *Date of literature search*: 1978/1980 - Jun 2008  *Meta-analysis*: yes  *Sub-group or sensitivity analysis*: yes (ICSI, frozen ET, and low quality studies) | *Population(s)*:  • twins conceived by IVF/ICSI | • multifetal or selective reduction | *Number of studies:* 12  • all retrospective cohorts  *Publication dates (range)*: 1992 - 2005  *Countries*: Australia (1), Belgium (1), Denmark (1), Finland (2), Germany (1), Greece (1), Hungary (1), Israel (1), Lebanon (1), Sweden (1), the UK (1)  *Number of patients*: 16,178  • : 4,385  • SC: 11,793 | • *age*: maternal age controlled for in all studies  • *BMI*: -  • *other*: unselected patients in most studies (i.e., all twin pregnancies/deliveries within a certain time period at a particular clinic or in a particular region) | • *ovarian stimulation protocol*: -  • n*umber of cycles*: -  • d*onor or autologous oocytes*: -  • f*rozen or fresh embryos*: fresh cycles only in 1 study; frozen cycles only in 1 study  • s*tage of embryo during transfer*: -  • *number of embryos transferred*: -  • q*uality of embryos transferred*: - | Primary:  *Effectiveness*: -  *Safety*:  • preterm birth rate  Secondary:  *Effectiveness*: -  *Safety*:  • moderate preterm birth rate  • mean duration of gestation | Primary:  • low birth weight rate  Secondary:  • mean birth weight  • very low birth weight rate  • extremely low birth weight rate  • intra uterine growth restriction |
|  |  | *Intervention(s)*:  • IVF/ICSI |  |  |  |  |  |  |
|  |  | *Comparator(s)*:  • spontaneous conception (SC) |  |  |  |  |  |  |
|  |  | *Outcome(s)*:  • preterm birth, low birth weight |  |  |  |  |  |  |
|  |  | *Design*:  • cohort studies  • case-control studies |  |  |  |  |  |  |
|  |  | *Other*:  • English language  • matching or adjustment for at least maternal age | • duplicate publications |  |  |  |  |  |
| McDonald et al. (2009) | *Country*: Canada (Ontario)  *Comparison*: IVF/ICSI vs. spontaneous conception  *Date of literature search*: 1978/1980 - Jun 2008  *Meta-analysis*: yes  *Sub-group or sensitivity analysis*: yes (ICSI, frozen ET, and low quality studies) | *Population(s)*:  • singletons conceived by IVF/ICSI | • multifetal or selective reduction | *Number of studies:* 17  • all retrospective cohorts  *Publication dates (range)*: 1990 - 2007  *Countries*: Belgium (2), Denmark (1), Finland (3), Hungary (1), Israel (2), the Netherlands (1), Sweden (2), the UK (1), the US (4)  *Number of patients*: 112,151  • : 31,032  • SC: 81,119 | • *age*: maternal age controlled for in all studies  • *BMI*: -  • *other*: unselected patients in most studies (i.e., all singleton pregnancies/deliveries within a certain time period at a particular clinic or in a particular region) | • *ovarian stimulation protocol*: -  • n*umber of cycles*: -  • d*onor or autologous oocytes*: -  • f*rozen or fresh embryos*: fresh cycles only in 2 studies; frozen cycles only in 1 study  • s*tage of embryo during transfer*: -  • *number of embryos transferred*: -  • q*uality of embryos transferred*: - | Primary:  *Effectiveness*: -  *Safety*:  • preterm birth rate  Secondary:  *Effectiveness*: -  *Safety*:  • moderate preterm birth rate  • mean duration of gestation | Primary:  • low birth weight rate  Secondary:  • mean birth weight  • very low birth weight rate  • extremely low birth weight rate  • intra uterine growth restriction |
|  |  | *Intervention(s)*:  • IVF/ICSI |  |  |  |  |  |  |
|  |  | *Comparator(s)*:  • spontaneous conception (SC) |  |  |  |  |  |  |
|  |  | *Outcome(s)*:  • preterm birth, low birth weight |  |  |  |  |  |  |
|  |  | *Design*:  • cohort studies  • case-control studies |  |  |  |  |  |  |
|  |  | *Other*:  • English language  • matching or adjustment for at least maternal age | • duplicate publications  • reviews, editorials, letters, case reports  • studies published only as abstracts |  |  |  |  |  |
| Hvidtjørn et al. (2009) | *Country*: Denmark, Norway, Sweden, the US  *Comparison*: ART vs. spontaneous conception  *Date of literature search*: Jan 1996 - Apr 2008  *Meta-analysis*: yes  *Sub-group or sensitivity analysis*: yes (singles vs. multiples) | *Population(s)*:  • children conceived by ART |  | *Number of studies:* 41*  • 3 prospective cohorts  • 36 retrospective cohorts  • 2 case-control studies  *11 on CP and/or ASD  *Publication dates (range)*: CP/ASD: 2002 - 2008  *Countries*:  CP/ASD: Croatia (1), Denmark (5), Finland (1), Israel (1), Sweden (3)  *Number of patients*: - | • *age*: -  • *BMI*: -  • *other*: unselected patients in most studies (i.e., all children conceived by an ART within a certain time period at a particular clinic or in a particular region) | • *ovarian stimulation protocol*: -  • n*umber of cycles*: -  • d*onor or autologous oocytes*: -  • f*rozen or fresh embryos*: -  • s*tage of embryo during transfer*: -  • *number of embryos transferred*: -  • q*uality of embryos transferred*: - | *Effectiveness*: -  *Safety*: - | • cerebral palsy rate  • autism spectrum disorder rate  • motor development  • behavioral development  • cognitive development  • mental development |
|  |  | *Intervention(s)*:  • ART (IVF/ICSI, intrauterine insemination (IUI), ovulation induction (OI)) |  |  |  |  |  |  |
|  |  | *Comparator(s)*:  • spontaneous conception (SC) |  |  |  |  |  |  |
|  |  | *Outcome(s)*:  • cerebral palsy (CP), autism spectrum disorder (ASD), developmental delay (DD) |  |  |  |  |  |  |
|  |  | *Design*: - |  |  |  |  |  |  |
|  |  | *Other*:  • original data  • follow-up period ≥1 year |  |  |  |  |  |  |
| Vitthala et al. (2009) | *Country*: the UK  *Comparison*: ART (no comparator)  *Date of literature search*: 1966/1974 - Jul 2007  *Meta-analysis*: yes  *Sub-group or sensitivity analysis*: yes (by diagnostic methods and type of ART) | *Population(s)*:  • women undergoing ART |  | *Number of studies:* 27  • 2 prospective cohorts  • 21 retrospective cohorts  • 4 cross-sectional studies (population surveys)  *Publication dates (range)*: 1993 - 2007  *Countries*: Belgium (1), Brazil (1), Denmark (1), Greece (1), Israel (4), Japan (1), the Netherlands (1), Norway (1), Poland (2), the UK (2), the US (12)  *Number of patients*: 87,932 | • *age*: -  • *BMI*: -  • *other*: - | • *ovarian stimulation protocol*: -  • n*umber of cycles*: -  • d*onor or autologous oocytes*: -  • f*rozen or fresh embryos*: frozen cycles only in 3 studies  • s*tage of embryo during transfer*: blastocyst embryos only in 9 studies  • *number of embryos transferred*: varies across studies (range 1->4)  • q*uality of embryos transferred*: - | *Effectiveness*: -  *Safety*: - | • monozygotic twinning rate |
|  |  | *Intervention(s)*:  • ART†† |  |  |  |  |  |  |
|  |  | *Comparator(s)*: - |  |  |  |  |  |  |
|  |  | *Outcome(s)*:  • monozygotic twinning (MZT) as a proportion of total number of pregnancies | • MZT not reported as a proportion of either total clinical pregnancies or live births  • MZT reported as a proportion of multiple pregnancies |  |  |  |  |  |
|  |  | *Design*: - |  |  |  |  |  |  |
|  |  | *Other*:  • - | • method of diagnosis or confirmation of zygosity after live birth not reported |  |  |  |  |  |
| Bertelsmann et al. (2008) | *Country*: Germany  *Comparison*: ICSI vs. 1) IVF, or 2) spontaneous conception  *Date of literature search*: up to May 2006  *Meta-analysis*: no  *Sub-group or sensitivity analysis*: no | *Population(s)*:  • children conceived by ICSI |  | *Number of studies:* 27*  malformations: 15  • 4 prospective cohorts  • 6 retrospective cohorts  • 2 cohorts with 1 prospective and 1 retrospective arm  • 3 registry reports  * plus 3 meta-analyses  imprinting defects: 12  • retrospective cohorts, case-control studies, case series and case reports  *Publication dates (range)*: malformations: 1998 – 2005, imprinting defects: 2000 - 2005  *Countries*: malformations: Australia (3), Belgium (2), Denmark (2), Germany (2), Japan (1), Spain (1), the US (2), multiple (2-several European and the US)  *Number of patients*: - | • *age*: -  • *BMI*: -  • *other*: unselected patients in most studies (i.e., all children conceived by an ART within a certain time period at a particular clinic or in a particular region) | • *ovarian stimulation protocol*: -  • n*umber of cycles*: -  • d*onor or autologous oocytes*: -  • f*rozen or fresh embryos*: -  • s*tage of embryo during transfer*: -  • *number of embryos transferred*: -  • q*uality of embryos transferred*: - | *Effectiveness*: -  *Safety*: - | • malformations  • imprinting disorders |
|  |  | *Intervention(s)*:  • ICSI |  |  |  |  |  |  |
|  |  | *Comparator(s)*:  • IVF  • spontaneous conception (SC) |  |  |  |  |  |  |
|  |  | *Outcome(s)*:  • malformations, imprinting defects |  |  |  |  |  |  |
|  |  | *Design*:  • cohort studies  • case-control studies |  |  |  |  |  |  |
|  |  | *Other*:  • odds ratio for major malformations directly stated or calculable from reported study data | • studies published before Jan 1998 |  |  |  |  |  |
| Middelburg et al. (2008) | *Country*: the Netherlands  *Comparison*: IVF/ICSI vs. spontaneous conception  *Date of literature search*: 1978/1989 - Dec 2007  *Meta-analysis*: no  *Sub-group or sensitivity analysis*: no | *Population(s)*:  • children conceived by autologous IVF/ICSI | • >10% of children born following OI only  • donor gametes  • adopted children  • surrogate mothers | *Number of studies:* 23  • 8 prospective cohorts  • 6 retrospective cohorts  • 9 registry reports  *Publication dates (range)*: 1989 - 2007  *Countries*: -  *Number of patients*: - | • *age*: maternal age controlled for in 7 registry reports; lower infant age limit (1.5-2) in 3 registry reports (range across studies: 0-20)  • *BMI*: -  • *other*: several other factors controlled for: -parity (5), plurality (7), smoking (3), socioeconomic status (3), duration of infertility (1) and infant-preterm birth (6), birth weight (3), birth defects (1) | • *ovarian stimulation protocol*: -  • n*umber of cycles*: -  • d*onor or autologous oocytes*: autologous only in all studies  • f*rozen or fresh embryos*: frozen included in 2 registry reports  • s*tage of embryo during transfer*: -  • *number of embryos transferred*: -  • q*uality of embryos transferred*: - | *Effectiveness*: -  *Safety*: - | • cerebral palsy  • epilepsy or convulsions  • mental retardation  • neuromotor development  • behavioral development  • cognitive development  • speech/language development |
|  |  | *Intervention(s)*:  • IVF/ICSI |  |  |  |  |  |  |
|  |  | *Comparator(s)*:  • spontaneous conception (SC) | • no SC control |  |  |  |  |  |
|  |  | *Outcome(s)*:  • neurodevelopmental outcome |  |  |  |  |  |  |
|  |  | *Design*: - |  |  |  |  |  |  |
|  |  | *Other*:  • good external validity | • sample size < 25  • follow-up not beyond neonatal period  • non-English language |  |  |  |  |  |
| Farhi and Fisch (2007) | *Country*: Israel  *Comparison*: ART vs. spontaneous conception, or ART (no comparator)  *Date of literature search*: 1985 - 2006  *Meta-analysis*: no  *Sub-group or sensitivity analysis*: no | *Population(s)*:  • children born to infertile population |  | *Number of studies:* -  *Publication dates (range)*: -  *Countries*: -  *Number of patients*: - | • *age*: -  • *BMI*: -  • *other*: - | • *ovarian stimulation protocol*: -  • n*umber of cycles*: -  • d*onor or autologous oocytes*: -  • f*rozen or fresh embryos*: -  • s*tage of embryo during transfer*: -  • *number of embryos transferred*: -  • q*uality of embryos transferred*: - | *Effectiveness*: -  *Safety*: - | • congenital malformations  • birth defects  • imprinting disorders |
|  |  | *Intervention(s)*:  • ART (ovarian stimulation, intrauterine insemination (IUI), artificial donor insemination, IVF/ICSI) |  |  |  |  |  |  |
|  |  | *Comparator(s)*:  • spontaneous conception (SC)  • none |  |  |  |  |  |  |
|  |  | *Outcome(s)*:  • major congenital malformations |  |  |  |  |  |  |
|  |  | *Design*: - |  |  |  |  |  |  |
|  |  | *Other*: - |  |  |  |  |  |  |
| List of abbreviations used in table (alphabetical order): ART = artificial reproductive technologies; BMI = body mass index; CC = clomiphene citrate; COS = controlled ovarian stimulation; DET = double embryo transfer; ET = embryo transfer; EM = expectant management; eSET = elective single embryo transfer; FET = frozen embryo transfer; FSH = follicle-stimulating hormone; GIFT = Gamete intrafallopian transfer; GnRHa = Gonadotropin-releasing hormone agonist; hMG = human menopausal gonadotropin; ICSI = intracytoplasmic sperm injection (ICSI); IVF = in vitro fertilization; IUI = intrauterine insemination; LH = luteinizing hormone; LHRHa = luteinizing hormone-releasing hormone agonist; MET = multiple embryo transfer; MZT = monozygotic twinning; NICU = neonatal intensive care unit; nIUI = natural cycle IUI; OHSS = ovarian hyperstimulation syndrome; OI = ovulation induction; OR = odds ratio; QET = four embryo transfer; RCT = randomized controlled trial; SC = spontaneous conception; SET = single embryo transfer; sIUI = stimulated IUI; TET = three embryo transfer; ZIFT = Zygote intrafallopian transfer  † Baseline rates calculated after initial embryo transfer; cumulative rates calculated after both fresh and frozen transfers from a single oocyte retrieval  ‡ Baseline rates calculated after a single treatment cycle; cumulative rates calculated after all cycles  § Both Blake et al. (2007) and Johnson, Blake, and Farquhar (2007) are updates of a systematic review published in 2005 with Blake et al. (2007) providing the most recent update; for Johnson, Blake, and Farquhar (2007), only additional data on monozygotic twinning, that was not provided in Blake et al. (2007), is reported  ¶ ART included IVF/ICSI (6 studies), controlled ovarian stimulation (COS) (1 study), COS and IVF (2 studies), GIFT and IVF (1 study), and was not specified in 3 studies; results not separated by treatment type  †† ART included IVF/ICSI (25 studies) and ovulation induction (OI) (2 studies); sub-group analysis by treatment type  ‡‡ Included comparisons of ART vs. SC (28 studies included in review; 16 in meta-analysis), SC in fertile women vs. subfertile women (7; 4), ART vanishing twin vs. singleton gestation (5; 0), IVF vs. ICSI (10; 5), fresh vs. frozen ET (10; 9), blastocyst vs. cleavage stage ET (4; 2), and SET vs. DET (4; 3) | | | | | | | | |
