# Supplementary material for: Assisted reproductive technologies (ARTs): Evaluation of evidence to support public policy development
Source: Reprod Health. 2014 Nov 7;11:76. doi: 10.1186/1742-4755-11-76 (PMC4233043; doi:10.1186/1742-4755-11-76)
Supplement: Supplementary file 4 — Additional file 4: Table S4: Table of included additional primary studies grouped by their primary comparison. (DOC 35 KB) [file 12978_2014_327_MOESM4_ESM.doc]

## Additional file: Table S4. Table of included additional primary studies grouped by their primary comparison.

| **Review** | **Details of Study** | **Patient Characteristics** | **Procedure details** | **Outcomes** | | **Quality** |
| --- | --- | --- | --- | --- | --- | --- |
|  |  |  |  | **Pregnancy/delivery** | **Neonatal/infant** |  |
| **Stage of embryo during transfer** | | | | | | |
| Fernando et al. (2012) | *Country*: Australia  *Comparison*: association between stage of embryo transfer and IVF safety  *Design*: retrospective cohort | *Number of patients*:  4,202 singleton deliveries  • cleavage: 2,486  • blastocyst: 1,716  • adjustments for maternal age, BMI, health insurance, smoking status, parity, gender, fresh versus frozen, IVF versus ICSI, number of embryos transferred, mode of delivery (vaginal or caesarean), and presence of vanishing twin | • *ovarian stimulation protocol*: ‘uncommon’ protocols excluded  • n*umber of cycles*: -  • d*onor or autologous oocytes*: autologous only  • f*rozen or fresh embryos*: fresh and frozen  • s*tage of embryo during transfer*: primary comparison  • *number of embryos transferred*: 1 or 2  • q*uality of embryos transferred*: - | *Effectiveness*: -  *Safety*:  • placenta previa  • placental abruption  • preeclampsia  • antepartum hemorrhage  • postpartum hemorrhage  • preterm birth  • very preterm birth | • low birth weight  • very low birth weight  • small for gestational age  • large for gestational age | *Oxford level of evidence*: 2b |
| Dar et al. (2013) | *Country*: Canada  *Comparison*: association between stage of embryo transfer and IVF safety  *Design*: retrospective cohort (registry) | *Number of patients*:  12,712 singleton births  • cleavage: 9,506  • blastocyst: 3,206  • adjustments for maternal age, year of treatment, parity, number of oocytes retrieved, IVF versus ICSI, number of embryos transferred and presence of vanishing twin | • *ovarian stimulation protocol*: -  • n*umber of cycles*: -  • d*onor or autologous oocytes*: -  • f*rozen or fresh embryos*: fresh only  • s*tage of embryo during transfer*: primary comparison  • *number of embryos transferred*: 1, 2, or ≥3  • q*uality of embryos transferred*: - | *Effectiveness*: -  *Safety*:  • preterm birth  • very preterm birth | • low birth weight  • very low birth weight  • congenital anomalies  • stillbirths  • neonatal deaths | *Oxford level of evidence*: 2b |
| **Embryo donation** | | | | | | |
| Keenan et al. (2012) | *Country*: Australia, Canada, Finland, New Zealand, the US, and the UK  *Comparison*: IVF with donor embryos compared to autologous fresh IVF, autologous frozen IVF and IVF with donor oocytes  *Design*: retrospective cohort | *Number of patients*:  • 7,042 donor ETs  • 876,883 autologous fresh ETs  • 268,986 autologous frozen ETs  • 81,050 ETs from fresh donor oocytes  • 35,967 ETs from frozen donor oocytes  • *age*: -  • *BMI*: -  • *other*: - | • *ovarian stimulation protocol*: -  • n*umber of cycles*: -  • d*onor or autologous oocytes*: primary comparison  • f*rozen or fresh embryos*: fresh and frozen  • s*tage of embryo during transfer*: -  • *number of embryos transferred*: -  • q*uality of embryos transferred*: - | *Effectiveness*:  • live births  *Safety*: - | *-* | *Oxford level of evidence*: 2b |
