# Supplementary material for: Assisted reproductive technologies (ARTs): Evaluation of evidence to support public policy development
Source: Reprod Health. 2014 Nov 7;11:76. doi: 10.1186/1742-4755-11-76 (PMC4233043; doi:10.1186/1742-4755-11-76)
Supplement: Supplementary file 5 — Additional file 5: Table S5: Quality of included reviews: Oxman & Guyatt index of scientific quality scoring system for systematic reviews. (DOC 69 KB) [file 12978_2014_327_MOESM5_ESM.doc]

## Additional file 5: Table S5. Quality of included reviews: Oxman & Guyatt index of scientific quality scoring system for systematic reviews.

| **Study** | **Search methods stated:** | **Search reasonably comprehensive:** | **Inclusion criteria reported:** | **Bias in study selection avoided:** | **Criteria used to assess validity reported:** | **Validity assessed using appropriate criteria:** | **Methods used to combine findings reported:** | **Findings combined appropriately:** | **Conclusions supported by data:** | **Scientific quality rating*:** |
| --- | --- | --- | --- | --- | --- | --- | --- | --- | --- | --- |
| **IVF/ICSI in comparison to non-invasive ART treatment options** | | | | | | | | | | |
| Thomopoulos et al. (2013) | Yes | Yes | Partially | Yes | No | Can’t tell | N/A | N/A | Yes | 3 |
| Pandian et al. (2011) | Yes | Yes | Yes | Yes | Yes | Yes | Yes | Yes | Yes | 7 |
| **Number of embryos transferred** | | | | | | | | | | |
| Grady et al. (2012) | Yes | Yes | Yes | Yes | Yes | Yes | Yes | Yes | Yes | 7 |
| McLernon et al. (2010) | Yes | Yes | Yes | Yes | Yes | Yes | Yes | Yes | Yes | 7 |
| Gelbaya et al. (2010) | Yes | Yes | Yes | Yes | Yes | Yes | Yes | Yes | Yes | 7 |
| Baruffi et al. (2009) | Yes | Yes | Yes | Yes | Partially | Can’t tell | Yes | Yes | Yes | 4 |
| Pandian et al. (2009) | Yes | Yes | Yes | Yes | Yes | Yes | Yes | Yes | Yes | 7 |
| Van Loendersloot et al. (2010) | Yes | Yes | Yes | Yes | Yes | Yes | Yes | Yes | Yes | 7 |
| **Fresh embryo transfer in comparison to frozen embryo transfer** | | | | | | | | | | |
| Roque et al. (2013) | Yes | Yes | Yes | Yes | Yes | Yes | Yes | Yes | Yes | 7 |
| Maheshwari et al. (2012) | Yes | Yes | Yes | Yes | Yes | Yes | Yes | Yes | Yes | 7 |
| Jee et al. (2009) | Yes | Yes | Yes | Yes | No | Can’t tell | Yes | Yes | Yes | 4 |
| Wennerholm et al. (2009) | Yes | Yes | Yes | Yes | No | Can’t tell | N/A | N/A | Yes | 4 |
| D’Angelo and Amso (2007) | Yes | Yes | Yes | Yes | Yes | Yes | Yes | Yes | Yes | 7 |
| **Stage of embryo during transfer** | | | | | | | | | | |
| Glujovsky et al. (2012) | Yes | Yes | Yes | Yes | Yes | Yes | Yes | Yes | Yes | 7 |
| Chang et al. (2009) | Yes | Yes | Yes | No | No | Can’t tell | Yes | Yes | Yes | 3 |
| Papanikolaou et al. (2008) | Yes | Yes | Yes | Yes | Partially | Yes | Yes | Yes | Yes | 6 |
| Johnson et al. (2007) | Yes | Yes | Yes | Yes | Yes | Yes | Yes | Yes | Yes | 7 |
| **Embryo donation** | | | | | | | | | | |
| Van der Hoorn et al. (2010) | Yes | Yes | Partially | Yes | No | Can’t tell | N/A | N/A | Yes | 3 |
| **IVF/ICSI in comparison to spontaneous conception** | | | | | | | | | | |
| Hansen et al. (2013) | Yes | Yes | Yes | Yes | Yes | Yes | Yes | Yes | Yes | 7 |
| Hart and Norman (2013) | Yes | Yes | Yes | Yes | No | Can’t tell | N/A | N/A | Yes | 4 |
| Hart and Norman (2013) | Yes | Yes | Yes | Yes | No | Can’t tell | N/A | N/A | Yes | 4 |
| Pinborg et al. (2013) | Yes | Yes | Yes | Yes | Yes | Yes | Yes | Yes | Yes | 7 |
| Pandey et al. (2012) | Yes | Yes | Yes | Yes | Yes | Yes | Yes | Yes | Yes | 7 |
| Wen et al. (2012) | Yes | Yes | Yes | Yes | No | Can’t tell | Yes | Yes | Yes | 4 |
| Rossi and D’Addario (2011) | Yes | Yes | Yes | Yes | No | Can’t tell | Yes | Yes | Yes | 4 |
| Wilson et al. (2011) | Yes | Yes | Yes | Yes | No | Can’t tell | N/A | N/A | Yes | 4 |
| McDonald et al. (2010) | Yes | Yes | Yes | Yes | Yes | Yes | Yes | Yes | Yes | 7 |
| McDonald et al. (2009) | Yes | Yes | Yes | Yes | Yes | Yes | Yes | Yes | Yes | 7 |
| Hvidtjørn et al. (2009) | Yes | Yes | Yes | Yes | Yes | Yes | Yes | Yes | Yes | 7 |
| Vitthala et al. (2009) | Yes | Yes | Yes | Yes | Yes | Can’t tell | Yes | Yes | Yes | 5 |
| Bertelsmann et al. (2008) | Yes | Yes | Yes | Yes | No | Can’t tell | N/A | N/A | Yes | 4 |
| Middelburg et al. (2008) | Yes | Yes | Yes | Yes | Yes | Yes | N/A | N/A | Yes | 7 |
| Farhi and Fisch (2007) | Yes | Yes | Yes | Yes | No | Can’t tell | N/A | N/A | Yes | 4 |
| *Scientific quality rating:  - >1 “can’t tell” = review likely to have minor flaws at best, difficult to rule out major flaws = score 4 or lower  - “no” on Qs 2, 4, 6, or 8 = review is likely to have major flaws = score of 3 or less depending on number and degree of flaws  - extensive flaws: score 1-3, minor flaws: score 4-7 (if major 4-5, if minimal 6-7) | | | | | | | | | | |
