# Supplementary material for: Assisted reproductive technologies (ARTs): Evaluation of evidence to support public policy development
Source: Reprod Health. 2014 Nov 7;11:76. doi: 10.1186/1742-4755-11-76 (PMC4233043; doi:10.1186/1742-4755-11-76)
Supplement: Supplementary file 6 — Additional file 6: Table S6: Safety during pregnancy and delivery. (DOC 281 KB) [file 12978_2014_327_MOESM6_ESM.doc]

## Additional file 6: Table S6. Safety during pregnancy and delivery.

| **Study** | **Study Groups** | **Event Rate n/N (%), Odds Ratio (95%CI)*, and p-value** | | | | | | | | | |
| --- | --- | --- | --- | --- | --- | --- | --- | --- | --- | --- | --- |
|  |  | OHSS | Ectopic pregnancy | Gestational diabetes | PIH or preeclampsia | Placenta praevia | Placental abruption | Preterm delivery/birth | Early preterm delivery/birth | Caesarean section delivery | Other |
| **IVF/ICSI in comparison to non-invasive ART treatment options** | | | | | | | | | | | |
| Pandian et al. (2011)  *Meta-analysis* | Number of studies | 1 |  |  |  |  |  |  |  |  |  |
|  | sIUI (≤6 cycles) (ref.) | 2/59 (3.4%) |  |  |  |  |  |  |  |  |  |
|  | IVF (≤6 cycles) | 3/59 (5.1%) |  |  |  |  |  |  |  |  |  |
|  | OR (95% CI)  p-value | 1.53 (0.25, 9.49)  0.65 |  |  |  |  |  |  |  |  |  |
| **Number of embryos transferred** | | | | | | | | | | | |
| Pinborg et al. (2013)  *Meta-analysis* | Number of studies |  |  |  |  |  |  | 3 |  |  |  |
|  | SET singletons |  |  |  |  |  |  | nr |  |  |  |
|  | DET singletons (ref.) |  |  |  |  |  |  | nr |  |  |  |
|  | OR (95% CI)  p-value |  |  |  |  |  |  | 0.83 (0.64, 1.06) nr |  |  |  |
| Grady et al. (2012)  *Meta-analysis* | Number of studies |  | 3 (RCTs) | 1 (RCT) | 1 (RCT) |  |  | 4 (RCTs) | 2 (RCTs) |  | 1 (RCT)† |
|  | eSET |  | 1/165 (0.6%) | 1/128 (0.8%) | 9/128 (7.0%) |  |  | 27/388 (7.0%) | 7/334 (2.1%) |  | 1/128 (0.8%) |
|  | DET (ref.) |  | 5/232 (2.2%) | 5/142 (3.5%) | 11/142 (7.7%) |  |  | 96/446 (21.5%) | 18/388 (4.6%) |  | 12/142 (8.5%) |
|  | RR (95% CI)  p-value |  | 0.42 (0.09,2.01)  nr | nr  nr | nr  nr |  |  | 0.37 (0.25,0.55)  <0.00001 | 0.53 (0.17,1.60)  nr |  | nr  nr |
|  | Number of studies |  | 3 (cohorts) | 1 (cohort) | 1 (cohort) | 1 (cohort) | 1 (cohort) | 3 (cohorts) |  | 1 (cohort) |  |
|  | eSET |  | 8/261 (3.1%) | 28/269 (10.4%) | 6/269 (2.2%) | 7/269 (2.6%) | 0/269 (0) | 46/351 (13.1%) |  | 11/56 (19.6%) |  |
|  | DET (ref.) |  | 15/534 (2.8%) | 17/230 (7.4%) | 8/230 (3.5%) | 6/230 (2.6%) | 5/230 (2.2%) | 44/325 (13.5%) |  | 11/45 (24.4%) |  |
|  | RR (95% CI)  p-value |  | 1.07 (0.45,2.51)  nr | 1.69 (1.19, 2.42)  nr | nr  nr | nr  nr | nr  nr | 0.99 (0.43,2.29)  0.03 |  | nr  nr |  |
| McLernon et al. (2010)‡  *Meta-analysis* | Number of studies |  |  |  |  |  |  | nr | nr |  | nr§ |
|  | eSET |  |  |  |  |  |  | 23/181 (12.7%) | 1/181 (0.6%) |  | 6/181 (3.3%) |
|  | DET (ref.) |  |  |  |  |  |  | 85/284 (29.9%) | 16/284 (5.6%) |  | 29/284 (10.2%) |
|  | OR (95% CI)  p-value |  |  |  |  |  |  | 0.33 (0.20,0.55)¶  nr | 0.08 (0.01,0.65)**  nr |  | 0.30 (0.12,0.75)††  Nr |
| Gelbaya et al. (2010)  *Meta-analysis* | Number of studies |  | 3 |  |  |  |  | 2 |  |  |  |
|  | eSET |  | 1/458 (0.2%) |  |  |  |  | 3/36 (8.3%) |  |  |  |
|  | DET (ref.) |  | 4/454 (0.9%) |  |  |  |  | 11/47 (23.4%) |  |  |  |
|  | RR (95% CI)  p-value |  | 0.39 (0.08,1.99)  0.26 |  |  |  |  | 0.36 (0.11,1.18)  0.09 |  |  |  |
| **Stage of embryo during transfer** | | | | | | | | | | | |
| Fernando et al. (2013)  *Primary Study* |  |  |  |  |  |  |  |  |  |  | ‡‡ |
|  | Blastocyst |  |  |  | 65/1,716 (3.8%) | 64/1,716 (3.7%) | 5/1,716 (0.3%) | 165/1,716 (9.6%) | 37/1,716 (2.2%) |  | 35/1,716 (2.0%) |
|  | Cleavage (ref.) |  |  |  | 74/2,486 (3.0%) | 72/2,486 (2.9%) | 4/2,486 (0.2%) | 228/2,486 (9.2%) | 58/2,486 (2.3%) |  | 67/2,486 (2.7%) |
|  | Adj. OR (95% CI)  p-value |  |  |  | 1.72 (0.93, 3.20) 0.02 | 1.65 (0.92, 2.98) 0.03 | 0.65 (0.11, 3.93) 0.53 | 0.93 (0.66, 1.30) 0.57 | 0.65 (0.34, 1.24) 0.08 |  | 0.75 (0.39, 1.44) 0.25 |
| Dar et al. (2013)  *Primary Study* |  |  |  |  |  |  |  |  |  |  |  |
|  | Blastocyst |  |  |  |  |  |  | 548/3,194 (17.2%) | 95/3,194 (3.0%) |  |  |
|  | Cleavage (ref.) |  |  |  |  |  |  | 1,335/9,442 (14.1%) | 251/9,442 (2.7%) |  |  |
|  | Adj. OR (95% CI)  p-value |  |  |  |  |  |  | 1.32 (1.17, 1.49) <0.001 | nr  0.34 |  |  |
| **Fresh embryo transfer in comparison to frozen embryo transfer** | | | | | | | | | | | |
| Pinborg et al. (2013)  *Meta-analysis* | Number of studies |  |  |  |  |  |  | 9 |  |  |  |
|  | Fresh ET singletons |  |  |  |  |  |  | nr |  |  |  |
|  | Frozen ET singletons (ref.) |  |  |  |  |  |  | nr |  |  |  |
|  | OR (95% CI)  p-value |  |  |  |  |  |  | 0.85 (0.76, 0.94) nr |  |  |  |
| Maheshwari et al. (2012)  *Meta-analysis* | Number of studies |  |  |  |  | 2 | 2 | 9 | 4 | 5 | 2‡‡ |
|  | Fresh ET (ref.) |  |  |  |  | nr | nr | 2905/27686 (10.5%) | nr | 4835/16740 (28.9%) | 358/7000 (5.1%) |
|  | Frozen ET |  |  |  |  | nr | nr | 941/10017 (9.4%) | nr | 1910/5435 (35.1%) | 124/3875 (3.2%) |
|  | RR (95% CI)  p-value |  |  |  |  | 0.71 (0.53-0.95)  nr | 0.44 (0.24, 0.83)  nr | 0.84 (0.78, 0.90)  <0.00001 | 0.73 (0.50, 1.08)  nr | 1.10 (1.05, 1.15)  <0.0001 | 0.67 (0.55, 0.81)  <0.0001 |
| Jee et al. (2009)  *Meta-analysis* | Number of studies |  | 7 |  |  |  |  |  |  |  |  |
|  | Fresh ET (ref.) |  | 162/10934 (1.5%) |  |  |  |  |  |  |  |  |
|  | Frozen ET |  | 49/2125 (2.3%) |  |  |  |  |  |  |  |  |
|  | OR (95% CI)  p-value |  | 1.66 (0.62,4.41)  0.31 |  |  |  |  |  |  |  |  |
| • SGA: studies with cleavage stage embryos only | Number of studies |  | 3 |  |  |  |  |  |  |  |  |
|  | Fresh ET (ref.) |  | 81/2813 (2.9%) |  |  |  |  |  |  |  |  |
|  | Frozen ET |  | 29/1244 (2.3%) |  |  |  |  |  |  |  |  |
|  | OR (95% CI)  p-value |  | 0.83 (0.20,3.49)  0.80 |  |  |  |  |  |  |  |  |
| • SGA: studies with cleavage and blastocyst embryos | Number of studies |  | 3 |  |  |  |  |  |  |  |  |
|  | Fresh ET (ref.) |  | 71/7557 (0.9%) |  |  |  |  |  |  |  |  |
|  | Frozen ET |  | 15/701 (2.1%) |  |  |  |  |  |  |  |  |
|  | OR (95% CI)  p-value |  | 3.47 (0.51,23.63)  0.20 |  |  |  |  |  |  |  |  |
| • SGA: studies with blastocyst stage embryos only | Number of studies |  | 1 |  |  |  |  |  |  |  |  |
|  | Fresh ET (ref.) |  | 10/564 (1.8%) |  |  |  |  |  |  |  |  |
|  | Frozen ET |  | 5/180 (2.8%) |  |  |  |  |  |  |  |  |
|  | OR (95% CI)  p-value |  | 1.58 (0.53,4.69)  0.41 |  |  |  |  |  |  |  |  |
| D’Angelo and Amso (2007)  *Meta-analysis* | Number of studies | 1 |  |  |  |  |  |  |  |  | 1§§ |
|  | Fresh ET (ref.) | 4/67 (6.0%) |  |  |  |  |  |  |  |  | 4/67 (6.0%) |
|  | Frozen ET | 0/58 (0) |  |  |  |  |  |  |  |  | 0/58 (0) |
|  | OR (95% CI)  p-value | 0.12 (0.01, 2.29)  0.16 |  |  |  |  |  |  |  |  | 0.12 (0.01, 2.29)  0.16 |
| **IVF/ICSI in comparison to spontaneous conception** | | | | | | | | | | | |
| Pinborg et al. (2013)  *Meta-analysis*  •SC in subfertile women | Number of studies |  |  |  |  |  |  | 2 |  |  |  |
|  | IVF/ICSI singletons |  |  |  |  |  |  | nr |  |  |  |
|  | SC singletons (ref.) |  |  |  |  |  |  | nr |  |  |  |
|  | OR (95% CI)  p-value |  |  |  |  |  |  | 1.35 (1.22, 1.50) nr |  |  |  |
| • IVF/ICSI sibling and SC sibling | Number of studies |  |  |  |  |  |  | 2 |  |  |  |
|  | IVF/ICSI singletons |  |  |  |  |  |  | nr |  |  |  |
|  | SC singletons (ref.) |  |  |  |  |  |  | nr |  |  |  |
|  | OR (95% CI)  p-value |  |  |  |  |  |  | 1.27 (1.08, 1.49)  nr |  |  |  |
| • ART (OI, IUI, IVF/ICSI) vs. SC in fertile women | Number of studies |  |  |  |  |  |  | 5 |  |  |  |
|  | ART singletons |  |  |  |  |  |  | nr |  |  |  |
|  | SC singletons (ref.) |  |  |  |  |  |  | nr |  |  |  |
|  | OR (95% CI)  p-value |  |  |  |  |  |  | 1.45 (1.21, 1.74)  nr |  |  |  |
| • IVF/ICSI frozen ET vs. SC in general population | Number of studies |  |  |  |  |  |  | 3 |  |  |  |
|  | IVF/ICSI singletons |  |  |  |  |  |  | nr |  |  |  |
|  | SC singletons (ref.) |  |  |  |  |  |  | nr |  |  |  |
|  | OR (95% CI)  p-value |  |  |  |  |  |  | 1.20 (0.98, 1.46)  nr |  |  |  |
| • IVF vs. ICSI | Number of studies |  |  |  |  |  |  | 5 |  |  |  |
|  | IVF singletons |  |  |  |  |  |  | nr |  |  |  |
|  | ICSI singletons (ref.) |  |  |  |  |  |  | nr |  |  |  |
|  | OR (95% CI)  p-value |  |  |  |  |  |  | 0.80 (0.69, 0.93)  Nr |  |  |  |
| Grady et al. (2012)  *Meta-analysis* | Number of studies |  | 1 | 1 | 1 | 1 | 1 | 2 | 2 |  |  |
|  | eSET |  | 27/269 (10.0%) | 28/269 (10.4%) | 6/269 (2.2%) | 7/269 (2.6%) | 0/269 (0) | 58/520 (11.2%) | 5/520 (1.0%) |  |  |
|  | SC (ref.) |  | 236/15037 (1.6%) | 924/15037 (6.1%) | 246/15037 (1.6%) | 65/15037 (0.4%) | 59/15037 (0.4%) | 4335/74572 (5.8%) | 576/74572 (0.8%) |  |  |
|  | RR (95% CI)  p-value |  | 6.40 (4.38,9.35)  nr | 1.69 (1.19,2.42)  nr | 1.36 (0.61,3.04)  nr | 6.02 (2.79,13.01)  nr | 0.47 (0.03,7.55)  nr | 2.13 (1.26,3.61)  0.005 | 1.31 (0.54,3.15)  nr |  |  |
| Pandey et al. (2012)  *Meta-analysis* | Number of studies |  |  | 6 | 15 | 13‡‡ |  | 22 | 11 | 17 | 7† |
|  | IVF/ICSI singletons |  |  | nr | 982/16923 (5.8%) | 879/20807 (4.2%) |  | 2204/27819 (7.9%) | nr | nr | Nr |
|  | SC singletons (ref.) |  |  | nr | 17305/589391 (2.9%) | 4913/608731 (0.8%) |  | 31260/614400 (5.1%) | nr | nr | nr |
|  | RR (95% CI)  p-value |  |  | 1.48 (1.33, 1.66)  nr | 1.49 (1.39, 1.59)  <0.00001 | 2.49 (2.30, 2.69)  <0.00001 |  | 1.54 (1.47, 1.62)  <0.00001 | 1.68 (1.48, 1.91)  nr | 1.56 (1.01, 1.60)  nr | 1.16 (1.07, 1.26)  nr |
| Rossi and D’Addario (2011)***  *Meta-analysis* | Number of studies |  |  |  |  |  |  | 5 (C) |  | 4 (C) | 3 (C)§ |
|  | ART |  |  |  |  |  |  | 2576/4664 (55.2%) |  | 371/610 (60.8%) | 351/4322 (8.1%) |
|  | SC (ref.) |  |  |  |  |  |  | 3095/5837 (53.0%) |  | 365/722 (50.5%) | 439/5495 (8.0%) |
|  | OR (95% CI)  p-value |  |  |  |  |  |  | 1.30 (1.03, 1.65)  0.03 |  | 1.70 (1.35, 2.14)  <0.0001 | 1.11 (0.78, 1.59)  0.56 |
|  | Number of studies |  |  |  |  |  |  | 6 (NC) |  | 6 (NC) | 4 (NC)§ |
|  | ART |  |  |  |  |  |  | 1789/2457 (70.2%) |  | 1186/1650 (71.8%) | 552/2356 (23.4%) |
|  | SC (ref.) |  |  |  |  |  |  | 5598/9244 (60.5%) |  | 2895/5838 (49.6%) | 1607/8747 (18.4%) |
|  | OR (95% CI)  p-value |  |  |  |  |  |  | 1.08 (0.76, 1.55)  0.66 |  | 2.34 (1.55, 3.54)  <0.0001 | 1.12 (0.80, 1.57)  0.52 |
| • SGA: unlike sex twins | Number of studies |  |  |  |  |  |  | 3 (NC) |  |  | 3 (NC)§ |
|  | ART |  |  |  |  |  |  | 1459/2490 (58.6%) |  |  | 268/2490 (10.8%) |
|  | SC (ref.) |  |  |  |  |  |  | 1717/3467 (49.5%) |  |  | 285/3467 (8.2%) |
|  | OR (95% CI)  p-value |  |  |  |  |  |  | 1.51 (1.25, 1.83)  <0.0001 |  |  | 1.37 (0.94, 2.00)  0.10 |
| McDonald et al. (2010)†††  *Meta-analysis* | Number of studies |  |  |  |  |  |  | 11 |  |  | 5§ |
|  | IVF/ICSI twins |  |  |  |  |  |  | nr |  |  | nr |
|  | SC twins (ref.) |  |  |  |  |  |  | nr |  |  | nr |
|  | OR (95% CI)  p-value |  |  |  |  |  |  | 1.23 (1.09, 1.41)  0.003 |  |  | 1.63 (1.17, 2.27)  nr |
| • SGA: IVF alone | Number of studies |  |  |  |  |  |  | 6 |  |  |  |
|  | IVF twins |  |  |  |  |  |  | nr |  |  |  |
|  | SC twins (ref.) |  |  |  |  |  |  | nr |  |  |  |
|  | OR (95% CI)  p-value |  |  |  |  |  |  | 2.17 (1.39, 3.38)  nr |  |  |  |
| • SGA: IVF+ICSI | Number of studies |  |  |  |  |  |  | 5 |  |  |  |
|  | ICSI twins |  |  |  |  |  |  | nr |  |  |  |
|  | SC twins (ref.) |  |  |  |  |  |  | nr |  |  |  |
|  | OR (95% CI)  p-value |  |  |  |  |  |  | 1.25 (0.93, 1.68)  nr |  |  |  |
| • SGA: fresh embryos only | Number of studies |  |  |  |  |  |  | 1 |  |  |  |
|  | IVF/ICSI twins |  |  |  |  |  |  | nr |  |  |  |
|  | SC twins (ref.) |  |  |  |  |  |  | nr |  |  |  |
|  | OR (95% CI)  p-value |  |  |  |  |  |  | 1.00 (0.58, 1.73)  nr |  |  |  |
| • SGA: frozen embryos only | Number of studies |  |  |  |  |  |  | 1 |  |  |  |
|  | IVF/ICSI twins |  |  |  |  |  |  | nr |  |  |  |
|  | SC twins (ref.) |  |  |  |  |  |  | nr |  |  |  |
|  | OR (95% CI)  p-value |  |  |  |  |  |  | 1.85 (0.81, 4.23)  nr |  |  |  |
| McDonald et al. (2009)†††  *Meta-analysis* | Number of studies |  |  |  |  |  |  | 15 |  |  | 5§ |
|  | IVF/ICSI singletons |  |  |  |  |  |  | nr |  |  | nr |
|  | SC singletons (ref.) |  |  |  |  |  |  | nr |  |  | nr |
|  | OR (95% CI)  p-value |  |  |  |  |  |  | 1.84 (1.54, 2.21)  <0.00001 |  |  | 2.27 (1.73, 2.97)  nr |
| • SGA: IVF alone | Number of studies |  |  |  |  |  |  | 8 |  |  |  |
|  | IVF singletons |  |  |  |  |  |  | nr |  |  |  |
|  | SC singletons (ref.) |  |  |  |  |  |  | nr |  |  |  |
|  | OR (95% CI)  p-value |  |  |  |  |  |  | 2.14 (1.58, 2.89)  nr |  |  |  |
| • SGA: IVF+ICSI | Number of studies |  |  |  |  |  |  | 7 |  |  |  |
|  | ICSI singletons |  |  |  |  |  |  | nr |  |  |  |
|  | SC singletons (ref.) |  |  |  |  |  |  | nr |  |  |  |
|  | OR (95% CI)  p-value |  |  |  |  |  |  | 1.87 (1.34, 2.62)  nr |  |  |  |
| • SGA: fresh embryos only | Number of studies |  |  |  |  |  |  | 2 |  |  |  |
|  | IVF/ICSI singletons |  |  |  |  |  |  | nr |  |  |  |
|  | SC singletons (ref.) |  |  |  |  |  |  | nr |  |  |  |
|  | OR (95% CI)  p-value |  |  |  |  |  |  | 2.23 (1.27, 3.93)  nr |  |  |  |
| • SGA: frozen embryos only | Number of studies |  |  |  |  |  |  | 1 |  |  |  |
|  | IVF/ICSI singletons |  |  |  |  |  |  | nr |  |  |  |
|  | SC singletons (ref.) |  |  |  |  |  |  | nr |  |  |  |
|  | OR (95% CI)  p-value |  |  |  |  |  |  | 1.00 (0.39, 2.59)  nr |  |  |  |
| * Risk ratio in Grady et al. (2012), Gelbaya et al. (2010), and Pandey et al. (2012)  ‡ Meta-analysis of individual patient data  § Moderate preterm birth rate  ¶ Adjusted OR; no significant covariates found  ** Adjusted for significant covariates (maternal BMI and duration of infertility)  †† Adjusted for significant covariates (maternal age)  ‡‡ Antepartum hemorrhage (placenta praevia and placental abruption)  §§ Hospital admission rate  *** “Controlled” studies (C) are those that controlled for basic maternal characteristics, such as age, and “non-controlled” (NC) studies are those that did not control for potential confounding factors  ††† Combined OR obtained by pooling adjusted ORs (studies matched or adjusted for at least age, and often other factors such as parity and smoking)  Definitions:  • Ectopic pregnancy: diagnosed in most studies by ultrasound visualization of gestational sac in fallopian tube; ectopic pregnancy rates reported per pregnancy in Grady et al. (2012) and Jee et al. (2009), and per woman/couple in Gelbaya et al. (2010)  • Gestational diabetes = high blood sugar diagnosed during pregnancy  • Pregnancy-induced hypertension (PIH) = high blood pressure during pregnancy  • Preeclampsia = development of high blood pressure and protein in the urine during the 2^nd^-3^rd^ trimesters of pregnancy  • Placenta praevia = implantation of the placenta over or near the cervix during the 2^nd^-3^rd^ trimesters of pregnancy  • Gestation: term birth/delivery = birth/delivery at >37 weeks; preterm birth/delivery (PTB or PTD) = birth or delivery at <37 weeks gestation; moderate preterm birth/delivery = birth or delivery at <34 weeks gestation (<33 in McDonald et al.), early or very preterm birth/delivery = birth or delivery at <32 weeks gestation; preterm birth rates reported per pregnancy in Grady et al. (2012), and per delivery or live birth in McLernon et al. (2010), Gelbaya et al. (2010), Rossi and D’Addario (2011), and McDonald et al. (2009; 2010) | | | | | | | | | | | |
