# Supplementary material for: Assisted reproductive technologies (ARTs): Evaluation of evidence to support public policy development
Source: Reprod Health. 2014 Nov 7;11:76. doi: 10.1186/1742-4755-11-76 (PMC4233043; doi:10.1186/1742-4755-11-76)
Supplement: Supplementary file 7 — Additional file 7: Table S7: Neonatal/infant safety. (DOC 310 KB) [file 12978_2014_327_MOESM7_ESM.doc]

## Additional file 7: Table S7. Neonatal/infant safety.

| **Review** | **Study Groups** | **Event Rate n/N (%), Odds Ratio (95%CI)*, and p-value** | | | | | | | | | |
| --- | --- | --- | --- | --- | --- | --- | --- | --- | --- | --- | --- |
|  |  | Monozygotic twinning | Low birth weight | Very low birth weight | Extremely low birth weight | Neonatal mortality | Perinatal mortality | NICU admission | Birth defects | Congenital malformations | Other |
| **Number of embryos transferred** | | | | | | | | | | | |
| Grady et al. (2012)  *Meta-analysis* | Number of studies |  | 3 (RCTs) | 1 (RCT) |  | 1 (RCT) | 2 (RCTs) |  |  | 1 (RCT) | 1 (RCT)† |
|  | eSET |  | 13/183 (7.1%) | 5/129 (3.9%) |  | 0/129 (0%) | 1/141 (0.7%) |  |  | 4/129 (3.1%) | 4/129 (3.1%) |
|  | DET (ref.) |  | 72/247 (29.1%) | 14/189 (7.4%) |  | 2/189 (1.1%) | 2/199 (1.0%) |  |  | 9/189 (4.8%) | 8/189 (4.2%) |
|  | RR (95% CI)  p-value |  | 0.25 (0.15, 0.45)  <0.00001 | nr  nr |  | nr  nr | 0.74 (0.09, 5.94)  nr |  |  | nr  nr | 0.73 (0.23, 2.38)  nr |
|  | Number of studies |  | 3 (cohorts) | 2 (cohorts) |  | 3 (cohorts) | 2 (cohorts) | 3 (cohorts) |  |  | 1 (cohort)‡ |
|  | eSET |  | 17/351 (4.8%) | 3/295 (1.0%) |  | 0/387 (0%) | 4/295 (1.4%) | 11/351 (3.1%) |  |  | 10/269 (3.7%) |
|  | DET (ref.) |  | 31/325 (9.5%) | 3/280 (1.1%) |  | 6/484 (1.2%) | 3/280 (1.1%) | 17/325 (5.2%) |  |  | 10/230 (4.3%) |
|  | RR (95% CI)  p-value |  | 0.51 (0.29, 0.91)  0.02 | 1.08 (0.23, 5.10)  nr |  | 0.23 (0.03, 2.00)  nr | 1.31 (0.28, 6.21)  nr | 0.59 (0.28, 1.24)  nr |  |  | 0.86 (0.36, 2.02)  nr |
| McLernon et al. (2010)§  *Meta-analysis* | Number of studies |  | nr |  |  |  |  |  |  |  |  |
|  | eSET |  | 14/181 (8%) |  |  |  |  |  |  |  |  |
|  | DET (ref.) |  | 69/284 (24%) |  |  |  |  |  |  |  |  |
|  | OR (95% CI)  p-value |  | 0.36 (0.15, 0.87)¶  nr |  |  |  |  |  |  |  |  |
| **Fresh embryo transfer in comparison to frozen embryo transfer** | | | | | | | | | | | |
| Maheshwari et al. (2012)  *Meta-analysis* | Number of studies |  | 8 | 4 |  |  | 6 | 4 |  | 3 | 2‡ |
|  | Fresh ET (ref.) |  | 2146/258000 (8.3%) | nr |  |  | 151/17424 (0.9%) | nr |  | nr | 115/3141 (3.7%) |
|  | Frozen ET |  | 494/8536 (5.8%) | nr |  |  | 42/5546 (0.8%) | nr |  | nr | 31/1933 (1.6%) |
|  | RR (95% CI)  p-value |  | 0.69 (0.62, 0.76)  <0.00001 | 0.72 (0.50, 1.04)  nr |  |  | 0.68 (0.48, 0.96)  0.03 | 1.00 (0.92, 1.08)  nr |  | 1.05 (0.81, 1.35)  nr | 0.45 (0.30, 0.66)  <0.0001 |
| **Stage of embryo during transfer** | | | | | | | | | | | |
| Chang et al. (2009)  *Meta-analysis* | Number of studies | 9 |  |  |  |  |  |  |  |  |  |
|  | Cleavage ET (ref.) | 130/31601 (0.4%) |  |  |  |  |  |  |  |  |  |
|  | Blastocyst ET | 153/9316 (1.6%) |  |  |  |  |  |  |  |  |  |
|  | OR (95% CI)  p-value | 3.04 (1.54, 6.01)  0.001 |  |  |  |  |  |  |  |  |  |
| • SGA: studies published before 2002 | Number of studies | 7 |  |  |  |  |  |  |  |  |  |
|  | Cleavage ET (ref.) | 118/30995 (0.4%) |  |  |  |  |  |  |  |  |  |
|  | Blastocyst ET | 144/8858 (0.6%) |  |  |  |  |  |  |  |  |  |
|  | OR (95% CI)  p-value | 4.05 (3.16, 5.18)  <0.00001 |  |  |  |  |  |  |  |  |  |
| • SGA: studies published after 2002 | Number of studies | 2 |  |  |  |  |  |  |  |  |  |
|  | Cleavage ET (ref.) | 12/606 (2.0%) |  |  |  |  |  |  |  |  |  |
|  | Blastocyst ET | 9/458 (2.0%) |  |  |  |  |  |  |  |  |  |
|  | OR (95% CI)  p-value | 1.00 (0.43, 2.32)  1.00 |  |  |  |  |  |  |  |  |  |
| Fernando et al. (2013)  *Primary Study* |  |  |  |  |  |  |  |  |  |  | ‡ |
|  | Blastocyst |  | 127/1,716 (7.4%) | 31/1,716 (1.8%) |  |  |  |  |  |  | 141/1,716 (8.2%) |
|  | Cleavage (ref.) |  | 181/2,486 (7.3%) | 47/2,486 (1.9%) |  |  |  |  |  |  | 214/2,486 (8.6%) |
|  | Adj. OR (95% CI)  p-value |  | 0.92 (0.62, 1.33) 0.52 | 0.73 (0.35, 1.52) 0.26 |  |  |  |  |  |  | 1.05 (0.73, 1.52) 0.71 |
| Dar et al. (2013)  *Primary Study* |  |  |  |  |  |  |  |  |  |  |  |
|  | Blastocyst |  | 274/2,985 (9.2%) | 50/2,985 (1.7%) |  | 13/3,206 (0.4%) |  |  |  | 78/3,206 (2.4%) | 33/3,206 (1.0%) |
|  | Cleavage (ref.) |  | 897/9,109 (9.8%) | 155/9,109 (1.7%) |  | 39/9,506 (0.4%) |  |  |  | 215/9,506 (2.3%) | 98/9,506 (1.0%) |
|  | Adj. OR (95% CI)  p-value |  | nr 0.28 | nr  0.92 |  | nr  0.97 |  |  |  | nr  0.58 | nr  0.99 |
| **IVF/ICSI in comparison to spontaneous conception** | | | | | | | | | | | |
| Hansen et al. (2013)  *Meta-analysis* | Number of studies |  |  |  |  |  |  |  | 45 |  |  |
|  | IVF/ICSI infants |  |  |  |  |  |  |  | Nr |  |  |
|  | SC infants (ref.) |  |  |  |  |  |  |  | Nr |  |  |
|  | OR (95% CI)  p-value |  |  |  |  |  |  |  | 1.32 (1.24, 1.42)  nr |  |  |
| • SGA: singletons | Number of studies |  |  |  |  |  |  |  | 23 |  |  |
|  | IVF/ICSI singletons |  |  |  |  |  |  |  | nr |  |  |
|  | SC singletons (ref.) |  |  |  |  |  |  |  | nr |  |  |
|  | OR (95% CI)  p-value |  |  |  |  |  |  |  | 1.36 (1.30, 1.43)  nr |  |  |
| • SGA: multiples | Number of studies |  |  |  |  |  |  |  | 27 |  |  |
|  | IVF/ICSI multiples |  |  |  |  |  |  |  | Nr |  |  |
|  | SC multiples (ref.) |  |  |  |  |  |  |  | Nr |  |  |
|  | OR (95% CI)  p-value |  |  |  |  |  |  |  | 1.11 (0.98, 1.26)  Nr |  |  |
| • SGA: twins, adjustment made for differences in zygosity | Number of studies |  |  |  |  |  |  |  | 12 |  |  |
|  | IVF/ICSI twins |  |  |  |  |  |  |  | Nr |  |  |
|  | SC twins (ref.) |  |  |  |  |  |  |  | Nr |  |  |
|  | OR (95% CI)  p-value |  |  |  |  |  |  |  | 1.26 (0.99, 1.60)  Nr |  |  |
| • SGA: major birth defects | Number of studies |  |  |  |  |  |  |  | 16 |  |  |
|  | IVF/ICSI infants |  |  |  |  |  |  |  | Nr |  |  |
|  | SC infants (ref.) |  |  |  |  |  |  |  | Nr |  |  |
|  | OR (95% CI)  p-value |  |  |  |  |  |  |  | 1.42 (1.29, 1.56)  Nr |  |  |
| • SGA: major birth defects - singletons | Number of studies |  |  |  |  |  |  |  | 10 |  |  |
|  | IVF/ICSI singletons |  |  |  |  |  |  |  | Nr |  |  |
|  | SC singletons (ref.) |  |  |  |  |  |  |  | Nr |  |  |
|  | OR (95% CI)  p-value |  |  |  |  |  |  |  | 1.41 (1.33, 1.50)  Nr |  |  |
| • SGA: major birth defects - multiples | Number of studies |  |  |  |  |  |  |  | 9 |  |  |
|  | IVF/ICSI multiples |  |  |  |  |  |  |  | Nr |  |  |
|  | SC multiples (ref.) |  |  |  |  |  |  |  | Nr |  |  |
|  | OR (95% CI)  p-value |  |  |  |  |  |  |  | 1.06 (0.83, 1.34)  Nr |  |  |
| Grady et al. (2012)  *Meta-analysis* | Number of studies |  | 2 | 2 |  | 1 |  | 1 |  |  | 1‡ |
|  | eSET infants |  | 27/520 (5.2%) | 5/520 (1.0%) |  | 0/269 (0%) |  | 8/269 (3.0%) |  |  | 10/269 (3.7%) |
|  | SC infants (ref.) |  | 3396/74572 (4.6%) | 552/74572 (0.7%) |  | 21/15037 (0.1%) |  | 227/15037 (1.5%) |  |  | 315/15037 (2.1%) |
|  | RR (95% CI)  p-value |  | 1.46 (0.91, 2.33)  0.11 | 1.50 (0.62, 3.62)  nr |  | 1.30 (0.08, 21.33)  nr |  | 1.97 (0.98, 3.95)  nr |  |  | 1.78 (0.96, 3.30)  Nr |
| Pandey et al. (2012)  *Meta-analysis* | Number of studies |  | 19 | 14 |  |  | 8 | 5 |  | 7 | 7‡ |
|  | IVF/ICSI singletons |  | nr | nr |  |  | 97/14054 (0.7%) | nr |  | 162/4382 (3.7%) | 487/13207 (3.7%) |
|  | SC singletons (ref.) |  | nr | nr |  |  | 1433/580514 (0.2%) | nr |  | 141/5324 (2.6%) | 14037/580810 (2.4%) |
|  | RR (95% CI)  p-value |  | 1.65 (1.56, 1.75)  nr | 1.93 (1.72, 2.17)  nr |  |  | 1.87 (1.48, 2.37)  <0.00001 | 1.58 (1.42, 1.77)  nr |  | 1.67 (1.33, 2.09)  nr | 1.39 (1.27, 1.52)  <0.00001 |
| Wen et al. (2012)**  *Meta-analysis* | Number of studies |  |  |  |  |  |  |  | 46 |  |  |
|  | IVF/ICSI infants |  |  |  |  |  |  |  | nr |  |  |
|  | SC infants (ref.) |  |  |  |  |  |  |  | nr |  |  |
|  | OR (95% CI)  p-value |  |  |  |  |  |  |  | 1.37 (1.26, 1.48)  0.000 |  |  |
| • SGA: IVF alone | Number of studies |  |  |  |  |  |  |  | 16 |  |  |
|  | IVF infants |  |  |  |  |  |  |  | nr |  |  |
|  | SC infants (ref.) |  |  |  |  |  |  |  | nr |  |  |
|  | OR (95% CI)  p-value |  |  |  |  |  |  |  | 1.30 (1.17, 1.46)  0.055 |  |  |
| • SGA: IVF+ICSI | Number of studies |  |  |  |  |  |  |  | 15 |  |  |
|  | ICSI infants |  |  |  |  |  |  |  | nr |  |  |
|  | SC infants (ref.) |  |  |  |  |  |  |  | nr |  |  |
|  | OR (95% CI)  p-value |  |  |  |  |  |  |  | 1.58 (1.27, 1.95)  0.001 |  |  |
| Rossi and D’Addario (2011)††  *Meta-analysis* | Number of studies |  | 3 (C) | 3 (C) |  |  | 6 (C) | 5 (C) | 4 (C) |  |  |
|  | ART twins |  | 2129/4366 (48.8%) | 397/4366 (9.1%) |  |  | 147/4820 (3.0%) | 3023/4628 (65.0%) | 53/1556 (3.4%) |  |  |
|  | SC twins (ref.) |  | 2631/5427 (48.5%) | 480/5427 (8.8%) |  |  | 148/5993 (2.5%) | 3829/5801 (66.0%) | 83/2729 (3.0%) |  |  |
|  | OR (95% CI)  p-value |  | 1.02 (0.94, 1.11)  0.58 | 1.05 (0.83, 1.33)  0.67 |  |  | 1.27 (1.00, 1.60)  0.05 | 0.99 (0.83, 1.18)  0.91 | 1.15 (0.80, 1.63)  0.45 |  |  |
|  | Number of studies |  | 4 (NC) | 4 (NC) |  |  | 6 (NC) | 5 (NC) | 5 (NC) |  |  |
|  | ART twins |  | 702/1459 (48.1%) | 173/1459 (11.8%) |  |  | 103/2379 (4.3%) | 220/950 (23.1%) | 137/2426 (5.6%) |  |  |
|  | SC twins (ref.) |  | 2219/5341 (41.5%) | 554/5341 (10.4%) |  |  | 347/9025 (3.8%) | 378/1741 (21.7%) | 307/9138 (3.3%) |  |  |
|  | OR (95% CI)  p-value |  | 1.10 (0.77, 1.59)  0.60 | 1.18 (0.72, 1.95)  0.51 |  |  | 1.12 (0.89, 1.41)  0.34 | 1.13 (0.68, 1.86)  0.64 | 1.86 (0.58, 5.95)  0.30 |  |  |
| • SGA: unlike sex twins | Number of studies |  | 3 (NC) | 3 (NC) |  |  | 3 (NC) |  | 2 (NC) |  |  |
|  | ART twins |  | 1237/2490 (49.7%) | 234/2490 (9.4%) |  |  | 90/2490 (3.6%) |  | 63/1170 (5.4%) |  |  |
|  | SC twins (ref.) |  | 1471/3467 (42.4%) | 256/3467 (7.4%) |  |  | 64/3467 (1.8%) |  | 84/2147 (4.0%) |  |  |
|  | OR (95% CI)  p-value |  | 1.35 (1.19, 2.97)  <0.0001 | 1.28 (0.87, 1.88)  0.21 |  |  | 1.95 (1.41, 2.71)  <0.0001 |  | 1.38 (0.99, 1.93)  0.06 |  |  |
| McDonald et al. (2010)‡‡  *Meta-analysis* | Number of studies |  | 10 | 7 | 2 |  |  |  |  |  | 6‡ |
|  | IVF/ICSI twins |  | nr | nr | nr |  |  |  |  |  | nr |
|  | SC twins (ref.) |  | nr | nr | nr |  |  |  |  |  | nr |
|  | OR (95% CI)  p-value |  | 1.14 (1.06, 1.22)  0.0002 | 1.28 (0.73, 2.24)  nr | 0.88 (0.04, 19.40)  nr |  |  |  |  |  | 1.06 (0.72, 1.55)  nr |
| • SGA: IVF alone | Number of studies |  | 6 |  |  |  |  |  |  |  |  |
|  | IVF twins |  | nr |  |  |  |  |  |  |  |  |
|  | SC twins (ref.) |  | nr |  |  |  |  |  |  |  |  |
|  | OR (95% CI)  p-value |  | 1.71 (1.34, 2.18)  nr |  |  |  |  |  |  |  |  |
| • SGA: IVF+ICSI | Number of studies |  | 4 |  |  |  |  |  |  |  |  |
|  | ICSI twins |  | nr |  |  |  |  |  |  |  |  |
|  | SC twins (ref.) |  | nr |  |  |  |  |  |  |  |  |
|  | OR (95% CI)  p-value |  | 1.08 (0.80, 1.46)  nr |  |  |  |  |  |  |  |  |
| • SGA: fresh embryos only | Number of studies |  | 1 |  |  |  |  |  |  |  |  |
|  | IVF/ICSI twins |  | nr |  |  |  |  |  |  |  |  |
|  | SC twins (ref.) |  | nr |  |  |  |  |  |  |  |  |
|  | OR (95% CI)  p-value |  | 1.00 (0.58, 1.73)  nr |  |  |  |  |  |  |  |  |
| • SGA: frozen embryos only | Number of studies |  | 1 |  |  |  |  |  |  |  |  |
|  | IVF/ICSI twins |  | nr |  |  |  |  |  |  |  |  |
|  | SC twins (ref.) |  | nr |  |  |  |  |  |  |  |  |
|  | OR (95% CI)  p-value |  | 1.49 (0.83, 2.68)  nr |  |  |  |  |  |  |  |  |
| McDonald et al. (2009)‡‡  *Meta-analysis* | Number of studies |  | 12 | 8 | 1 |  |  |  |  |  | 8‡ |
|  | IVF/ICSI singletons |  | nr | nr | nr |  |  |  |  |  | nr |
|  | SC singletons (ref.) |  | nr | nr | nr |  |  |  |  |  | nr |
|  | OR (95% CI)  p-value |  | 1.60 (1.29, 1.98)  <0.0001 | 2.65 (1.83, 3.84)  nr | 3.02 (0.12, 74.66)  nr |  |  |  |  |  | 1.45 (1.04, 2.00)  nr |
| • SGA: IVF alone | Number of studies |  | 6 |  |  |  |  |  |  |  |  |
|  | IVF singletons |  | nr |  |  |  |  |  |  |  |  |
|  | SC singletons (ref.) |  | nr |  |  |  |  |  |  |  |  |
|  | OR (95% CI)  p-value |  | 1.85 (1.40, 2.43)  nr |  |  |  |  |  |  |  |  |
| • SGA: IVF+ICSI | Number of studies |  | 6 |  |  |  |  |  |  |  |  |
|  | ICSI singletons |  | nr |  |  |  |  |  |  |  |  |
|  | SC singletons (ref.) |  | nr |  |  |  |  |  |  |  |  |
|  | OR (95% CI)  p-value |  | 1.71 (1.26, 2.32)  nr |  |  |  |  |  |  |  |  |
| • SGA: fresh embryos only | Number of studies |  | 1 |  |  |  |  |  |  |  |  |
|  | IVF/ICSI singletons |  | nr |  |  |  |  |  |  |  |  |
|  | SC singletons (ref.) |  | nr |  |  |  |  |  |  |  |  |
|  | OR (95% CI)  p-value |  | 1.93 (0.75, 4.97)  nr |  |  |  |  |  |  |  |  |
| • SGA: frozen embryos only | Number of studies |  | 1 |  |  |  |  |  |  |  |  |
|  | IVF/ICSI singletons |  | nr |  |  |  |  |  |  |  |  |
|  | SC singletons (ref.) |  | nr |  |  |  |  |  |  |  |  |
|  | OR (95% CI)  p-value |  | 1.35 (0.46, 3.99)  nr |  |  |  |  |  |  |  |  |
| Hvidtjørn et al. (2009)  *Meta-analysis* | Number of studies |  |  |  |  |  |  |  |  |  | 3§§ |
|  | IVF/ICSI children |  |  |  |  |  |  |  |  |  | 88/19462 (0.5%) |
|  | SC children (ref.) |  |  |  |  |  |  |  |  |  | 1063/432950 (0.2%) |
|  | OR (95% CI)  p-value |  |  |  |  |  |  |  |  |  | 2.18 (1.71, 2.77)  nr |
| • SGA: singletons | Number of studies |  |  |  |  |  |  |  |  |  | 3§§ |
|  | IVF/ICSI singletons |  |  |  |  |  |  |  |  |  | 41/12191 (0.3%) |
|  | SC singletons (ref.) |  |  |  |  |  |  |  |  |  | 1081/479615 (0.2%) |
|  | OR (95% CI)  p-value |  |  |  |  |  |  |  |  |  | 1.82 (1.31, 2.52)  nr |
| • SGA: twins | Number of studies |  |  |  |  |  |  |  |  |  | 3§§ |
|  | IVF/ICSI twins |  |  |  |  |  |  |  |  |  | 39/7069 (0.6%) |
|  | SC twins (ref.) |  |  |  |  |  |  |  |  |  | 72/14940 (0.5%) |
|  | OR (95% CI)  p-value |  |  |  |  |  |  |  |  |  | 1.00 (0.65, 1.52)  nr |
| Vitthala et al. (2009)  *Meta-analysis* | Number of studies | 27 |  |  |  |  |  |  |  |  |  |
|  | ART | 10783/2369682  1.7% (1.2, 2.2)*** |  |  |  |  |  |  |  |  |  |
| • SGA: IVF alone | Number of studies | 13 |  |  |  |  |  |  |  |  |  |
|  | IVF | 151/40682  0.89% (0.5, 1.3) |  |  |  |  |  |  |  |  |  |
| • SGA: IVF+ICSI | Number of studies | 6 |  |  |  |  |  |  |  |  |  |
|  | ICSI | 11/1220  1.2% (0.4, 2.2) |  |  |  |  |  |  |  |  |  |
| • SGA: frozen embryos only | Number of studies | 3 |  |  |  |  |  |  |  |  |  |
|  | IVF/ICSI | 4/163  3.0% (0.96, 6.12) |  |  |  |  |  |  |  |  |  |
| • SGA: blastocyst embryos only | Number of studies | 9 |  |  |  |  |  |  |  |  |  |
|  | IVF/ICSI | 173/9721  5.1% (2.9, 8.1) |  |  |  |  |  |  |  |  |  |
| • SGA: spontaneous conception | Number of studies | 2 |  |  |  |  |  |  |  |  |  |
|  | SC | nr  0.4% (0.4, 0.4) |  |  |  |  |  |  |  |  |  |
| * Risk ratio in Grady et al. (2012), Maheshwari et al. (2012), Gelbaya et al. (2010), and Pandey et al. (2012)  † Proportion of infants with an Apgar score <7 at 5 minutes  ‡ Proportion of infants small for gestational age or with intrauterine growth restriction  § Meta-analysis of individual patient data  ¶ Adjusted for significant covariates (gestational age)  ** Combined OR obtained by pooling adjusted ORs, when available, and crude ORs, where adjusted not available (33 studies matched or adjusted for factors such as maternal age, parity, and smoking)  †† “Controlled” studies (C) are those that controlled for basic maternal characteristics, such as age, and “non-controlled” (NC) studies are those that did not control for potential confounding factors  ‡‡ Combined OR obtained by pooling adjusted ORs (studies matched or adjusted for at least age, and often other factors such as parity and smoking)  §§ Proportion of infants with cerebral palsy  *** Weighted average (95% CI)  Definitions:  • Monozygotic twinning (MZT): diagnosis used by most studies = ultrasound visualization of >1 fetus with cardiac activity in the same gestational sac or a greater number of fetal hearts than the number of embryos transferred; a few studies used Weinberg’s method (rate estimated by the number of unlike sex twins at birth), post-natal placental histology, DNA analysis, or blood group analysis  • Birth weight: normal birth weight = birth weight >2,500 grams; low birth weight (LBW) = birth weight <2,500 grams; very low birth weight = birth weight <1,500 grams; extremely low birth weight = birth weight <1,000 grams; small for gestational age or intrauterine growth restriction (IUGR) = birth weight <10^th^ percentile for gestational age (except 2 studies in Maheshwari et al. (2012): 1-<22% expected mean birth weight according to reference population, and 1-<2 standard deviations of the mean for that gestation)  • Perinatal mortality defined by Rossi and D’Addario (2011) as stillbirth and early neonatal mortality (within 1 week of birth); not defined in Grady et al. (2012)  • Apgar score: method of assessing the health of newborn child immediately after birth (score of <3 = critically low, 4-6 = fairly low, 7-10 = generally normal) | | | | | | | | | | | |
