# Supplementary material for: Assisted reproductive technologies (ARTs): Evaluation of evidence to support public policy development
Source: Reprod Health. 2014 Nov 7;11:76. doi: 10.1186/1742-4755-11-76 (PMC4233043; doi:10.1186/1742-4755-11-76)
Supplement: Supplementary file 8 — Additional file 8: Table S8: Effectiveness: number of oocytes retrieved. (DOC 29 KB) [file 12978_2014_327_MOESM8_ESM.doc]

## Additional file 8: Table S8. Effectiveness: number of oocytes retrieved.

| **Review** | **Treatment Characteristics** | **Study Groups** | **Subgroups** | **Number of primary studies** | **Mean number of oocytes retrieved** | | | | **Heterogeneity** | |
| --- | --- | --- | --- | --- | --- | --- | --- | --- | --- | --- |
|  |  |  |  |  | **N** | **Mean ± SD** | **Mean difference**  **(95% CI)** | **P-value** | **I^2^ (%)** | **P-value** |
| **Fresh embryo transfer in comparison to frozen embryo transfer** | | | | | | | | | | |
| D’Angelo and Amso (2007)  *Meta-analysis* | • Autologous IVF/ICSI with cleavage stage (day 2-3) embryos  • 3-4 embryos per cycle  • 1 cycle per woman/couple | Fresh ET (ref.) |  | 1 | 67 | 19.80±4.30 | nr | nr | - | - |
|  |  | Frozen ET |  |  | 58 | 20.80±5.50 |  |  |  |  |
|  | | | | | | | | | | |
