# Supplementary material for: Assisted reproductive technologies (ARTs): Evaluation of evidence to support public policy development
Source: Reprod Health. 2014 Nov 7;11:76. doi: 10.1186/1742-4755-11-76 (PMC4233043; doi:10.1186/1742-4755-11-76)
Supplement: Supplementary file 9 — Additional file 9: Table S9: Effectiveness: embryo cryopreservation rate. (DOC 43 KB) [file 12978_2014_327_MOESM9_ESM.doc]

## Additional file 9: Table S9. Effectiveness: embryo cryopreservation rate.

| **Review** | **Treatment Characteristics** | **Study Groups** | **Subgroups** | **Number of primary studies** | **Embryo cryopreservation rate per woman or couple*** | | | | **Heterogeneity** | |
| --- | --- | --- | --- | --- | --- | --- | --- | --- | --- | --- |
|  |  |  |  |  | **n/N** | **%** | **Odds Ratio**  **(95% CI)** | **P-value** | **I^2^ (%)** | **P-value** |
| **Stage of embryo during transfer** | | | | | | | | | | |
| Glujovsky et al. (2012)  *Meta-analysis* | • Fresh, autologous or donor IVF/ICSI  • 1-5 embryos per cycle  • 1 or more cycles per woman/couple | Cleavage stage ET (ref.) |  | 11 | 571/910 | 62.7% | 0.35 (0.29, 0.43) | <0.00001 | 72% | 0.00011 |
|  |  | Blastocyst stage ET |  |  | 336/819 | 41.0% |  |  |  |  |
|  |  | Cleavage stage ET (ref.) | Studies with equal number of cleavage and blastocyst stage embryos transferred | 7 | 387/564 | 68.6% | 0.23 (0.11, 0.47) | 0.000068 | 78% | 0.00017 |
|  |  | Blastocyst stage ET |  |  | 271/554 | 48.9% |  |  |  |  |
|  |  | Cleavage stage ET (ref.) | Studies with more cleavage-stage embryos transferred than blastocyst stage | 4 | 184/346 | 53.2% | 0.25 (0.13, 0.48) | 0.000023 | 66% | 0.03 |
|  |  | Blastocyst stage ET |  |  | 65/265 | 24.5% |  |  |  |  |
|  |  | Cleavage stage ET (ref.) | Studies limited to patients with a good prognosis | 6 | 232/308 | 75.3% | 0.16 (0.08, 0.32) | <0.00001 | 55% | 0.05 |
|  |  | Blastocyst stage ET |  |  | 143/304 | 47.0% |  |  |  |  |
|  |  | Cleavage stage ET (ref.) | Studies with unselected patients | 4 | 260/440 | 59.1% | 0.38 (0.19, 0.76) | <0.00001 | 80% | 0.002 |
|  |  | Blastocyst stage ET |  |  | 177/434 | 40.8% |  |  |  |  |
| Papanikolaou et al. (2008)  *Meta-analysis* | • Fresh, autologous or donor IVF/ICSI  • 1-5 embryos per cycle  • 1 cycle per woman/couple | Cleavage stage ET (ref.) |  | 7 | 515/727 | 70.8% | 0.28 (0.14, 0.55) | 0.0002 | 84% | <0.00001 |
|  |  | Blastocyst stage ET |  |  | 371/703 | 52.8% |  |  |  |  |
| * Number of embryos cryopreserved (frozen) per woman/couple | | | | | | | | | | |
