# Supplementary material for: Assisted reproductive technologies (ARTs): Evaluation of evidence to support public policy development
Source: Reprod Health. 2014 Nov 7;11:76. doi: 10.1186/1742-4755-11-76 (PMC4233043; doi:10.1186/1742-4755-11-76)
Supplement: Supplementary file 10 — Additional file 10: Table S10: Effectiveness: cycle cancellation rate. (DOC 50 KB) [file 12978_2014_327_MOESM10_ESM.doc]

## Additional file 10: Table S10. Effectiveness: cycle cancellation rate.

| **Review** | **Treatment Characteristics** | **Study Groups** | **Subgroups** | **Number of primary studies** | **Cycle cancellation rate per woman or couple*** | | | | **Heterogeneity** | |
| --- | --- | --- | --- | --- | --- | --- | --- | --- | --- | --- |
|  |  |  |  |  | **n/N** | **%** | **Odds Ratio**  **(95% CI)** | **P-value** | **I^2^ (%)** | **P-value** |
| **Stage of embryo during transfer** | | | | | | | | | | |
| Glujovsky et al. (2012)  *Meta-analysis* | • Fresh, autologous or donor IVF/ICSI  • 1-5 embryos per cycle  • 1 or more cycles per woman/couple | Cleavage stage ET (ref.) |  | 16 | 42/1242 | 3.4% | 2.86 (1.96, 4.17) | <0.00001 | 20% | 0.26 |
|  |  | Blastocyst stage ET |  |  | 108/1217 | 8.9% |  |  |  |  |
|  |  | Cleavage stage ET (ref.) | Studies with equal number of cleavage and blastocyst stage embryos transferred | 7 | 25/670 | 3.7% | 2.70 (1.64, 4.35) | 0.00010 | 0 | 0.74 |
|  |  | Blastocyst stage ET |  |  | 58/651 | 8.9% |  |  |  |  |
|  |  | Cleavage stage ET (ref.) | Studies with SET in both groups | 1 | 8/176 | 4.5% | 1.41 (0.55, 3.57) | 0.47 | - | - |
|  |  | Blastocyst stage ET |  |  | 11/175 | 6.3% |  |  |  |  |
|  |  | Cleavage stage ET (ref.) | Studies with more cleavage-stage embryos transferred than blastocyst stage | 8 | 9/396 | 2.3% | 4.35 (2.17, 9.09) | 0.000038 | 36% | 0.15 |
|  |  | Blastocyst stage ET |  |  | 283/645 | 43.9% |  |  |  |  |
|  |  | Cleavage stage ET (ref.) | Studies limited to patients with a good prognosis | 9 | 16/665 | 2.4% | 1.49 (0.79, 2.86) | 0.22 | 0 | 0.47 |
|  |  | Blastocyst stage ET |  |  | 23/650 | 3.5% |  |  |  |  |
|  |  | Cleavage stage ET (ref.) | Studies limited to patients with a poor prognosis | 2 | 2/43 | 4.7% | 5.00 (0.93, 25.00) | 0.061 | 0 | 1.00 |
|  |  | Blastocyst stage ET |  |  | 6/34 | 17.6% |  |  |  |  |
|  |  | Cleavage stage ET (ref.) | Studies with unselected patients | 5 | 24/534 | 4.5% | 3.70 (2.33, 5.88) | <0.00001 | 2% | 0.40 |
|  |  | Blastocyst stage ET |  |  | 79/533 | 14.8% |  |  |  |  |
| Papanikolaou et al. (2008)  *Meta-analysis* | • Fresh, autologous or donor IVF/ICSI  • 1-5 embryos per cycle  • 1 cycle per woman/couple | Cleavage stage ET (ref.) |  | 8 | 39/838 | 4.7% | 2.21 (1.47, 3.32) | 0.0001 | 0 | 0.45 |
|  |  | Blastocyst stage ET |  |  | 77/816 | 9.4% |  |  |  |  |
|  |  | BMI ≥30 |  |  | nr | nr |  |  |  |  |
| * Number of cycles cancelled per woman/couple reaching oocyte retrieval in Papanikolaou et al. (2008); number of couples failing to transfer any embryos in Glujovsky et al. (2012) | | | | | | | | | | |
