# Supplementary material for: Assisted reproductive technologies (ARTs): Evaluation of evidence to support public policy development
Source: Reprod Health. 2014 Nov 7;11:76. doi: 10.1186/1742-4755-11-76 (PMC4233043; doi:10.1186/1742-4755-11-76)
Supplement: Supplementary file 11 — Additional file 11: Table S11: Effectiveness: implantation rate. (DOC 36 KB) [file 12978_2014_327_MOESM11_ESM.doc]

## Additional file 11: Table S11. Effectiveness: implantation rate.

| **Review** | **Treatment Characteristics** | **Study Groups** | **Subgroups** | **Number of primary studies** | **Implantation rate per number of embryos transferred*** | | | | **Heterogeneity** | |
| --- | --- | --- | --- | --- | --- | --- | --- | --- | --- | --- |
|  |  |  |  |  | **n/N** | **%** | **Odds Ratio**  **(95% CI)** | **P-value** | **I^2^ (%)** | **P-value** |
| **Number of embryos transferred** | | | | | | | | | | |
| Gelbaya et al. (2010)  *Meta-analysis* | • Fresh, autologous IVF/ICSI with cleavage stage (day 2-3) embryos  • 1 cycle per woman/couple | eSET (ref.) |  | 5 | 169/501 | 33.7% | 0.93 (0.79, 1.08)† | 0.31 | 0 | 0.88 |
|  |  | DET |  |  | 323/1036 | 31.2% |  |  |  |  |
| Baruffi et al. (2009)  *Meta-analysis* | • Fresh, autologous IVF/ICSI with cleavage (day 2-3) or blastocyst (day 5-6) stage embryos  • 1 cycle per woman/couple in most (2 studies with 1-2 cycles per woman) | SET (ref.) |  | 5 | 163/470 | 34.7% | 0.99 (0.78, 1.25) | 0.96 | 0 | 0.85 |
|  |  | DET |  |  | 338/980 | 34.5% |  |  |  |  |
|  |  | SET (ref.) | Studies with patients ≤35 years | 4 | 124/373 | 33.2% | 1.02 (0.78, 1.33) | 0.90 | nr (no het.) | Nr |
|  |  | DET |  |  | 267/790 | 33.8% |  |  |  |  |
| * Number of implantations per total number of embryos transferred; the number of implantations was counted as the number of gestational sacs with a fetal heartbeat as seen on an ultrasound in Gelbaya et al. (2010) and was not specified in Baruffi et al. (2009)  † Risk ratio | | | | | | | | | | |
