# Supplementary material for: Assisted reproductive technologies (ARTs): Evaluation of evidence to support public policy development
Source: Reprod Health. 2014 Nov 7;11:76. doi: 10.1186/1742-4755-11-76 (PMC4233043; doi:10.1186/1742-4755-11-76)
Supplement: Supplementary file 12 — Additional file 12: Table S12: Effectiveness: pregnancy rate. (DOC 100 KB) [file 12978_2014_327_MOESM12_ESM.doc]

## Additional file 12: Table S12. Effectiveness: pregnancy rate.

| **Review** | **Treatment Characteristics** | **Study Groups** | | **Subgroups** | | **Number of primary studies** | | **Pregnancy rate per woman or couple** | | | | | | | | **Heterogeneity** | | | |  |
| --- | --- | --- | --- | --- | --- | --- | --- | --- | --- | --- | --- | --- | --- | --- | --- | --- | --- | --- | --- | --- |
|  |  |  |  |  |  |  |  | **n/N** | | **%** | | **Odds Ratio**  **(95% CI)** | | **P-value** | | **I^2^ (%)** | | **P-value** | |  |
| **IVF/ICSI in comparison to other treatment options** | | | | | | | | | | | | | | | | | | | |  |
| Pandian et al. (2011)  *Meta-analysis* | • Fresh or frozen, autologous IVF with cleavage stage (day 2-3) or blastocyst (day 5-6) stage embryos  • 1-6 cycles per woman/couple | sIUI (≤3 cycles) (ref.) | | Clinical pregnancy  Treatment-naïve women | | 2 | | 26/116 | | 22.4% | | 1.10 (0.60, 2.03) | | 0.76 | | 0 | | 0.76 | |  |
|  |  | IVF (≤2 cycles) | |  |  |  |  | 28/116 | | 24.1% | |  |  |  |  |  |  |  |  |  |
|  |  | FSH-IUI (≤3 cycles) (ref.) | | Clinical pregnancy  Women who failed to achieve pregnancy with ≤3 cycles of CC-IUI | | 1 | | 50/169 | | 29.6% | | 12.78 (7.54, 21.65) | | <0.00001 | | - | | - | |  |
|  |  | IVF (≤6 cycles) | |  |  |  |  | 145/172 | | 84.3% | |  |  |  |  |  |  |  |  |  |
| **Number of embryos transferred** | | | | | | | | | | | | | | | | | | | |  |
| Gelbaya et al. (2010)  *Meta-analysis* | • Fresh, autologous IVF/ICSI with cleavage stage (day 2-3) embryos  • 1 cycle per woman/couple | eSET (ref.) | | Biochemical pregnancy* | | 3 | | 179/510 | | 35.1% | | 1.49 (7.30, 1.72)† | | <0.00001 | | nr | | nr | |  |
|  |  | DET | |  |  |  |  | 269/512 | | 52.5% | |  |  |  |  |  |  |  |  |  |
|  |  | eSET (ref.) | | Clinical pregnancy* | | 4 | | 71/194 | | 36.6% | | 1.35 (1.08, 1.69)† | | 0.010 | | nr | | nr | |  |
|  |  | DET | |  |  |  |  | 95/191 | | 49.7% | |  |  |  |  |  |  |  |  |  |
|  |  | eSET (ref.) | | Ongoing pregnancy* | | 2 | | 43/180 | | 23.9% | | 1.89 (1.39, 2.56)† | | <0.0001 | | nr | | nr | |  |
|  |  | DET | |  |  |  |  | 82/181 | | 45.3% | |  |  |  |  |  |  |  |  |  |
| Baruffi et al. (2009)  *Meta-analysis* | • Fresh, autologous IVF/ICSI with cleavage (day 2-3) or blastocyst (day 5-6) stage embryos  • 1 cycle per woman/couple in most (2 studies with 1-2 cycles per woman) | SET (ref.) | | Ongoing pregnancy | | 6 | | 187/661 | | 28.3% | | 2.06 (1.64, 2.60) | | <0.001 | | 0 | | 0.65 | |  |
|  |  | DET | |  |  |  |  | 294/660 | | 44.5% | |  |  |  |  |  |  |  |  |  |
|  |  | SET (ref.) | | Studies with patients ≤35 years | | 3 | | 131/450 | | 29.1% | | 1.91 (1.45, 2.53) | | <0.0001 | | nr (no het.) | | nr | |  |
|  |  | DET | |  |  |  |  | 201/452 | | 44.5% | |  |  |  |  |  |  |  |  |  |
| Pandian et al. (2009)  *Meta-analysis* | • Fresh, autologous or donor IVF/ICSI with cleavage stage (day 2-3) embryos  • 1-2 cycles per woman/couple | 1 SET (ref.) | | Clinical pregnancy | | 5 | | 198/630 | | 31.4% | | 2.21 (1.75, 2.79) | | <0.00001 | | 0 | | 0.80 | |  |
|  |  | 1 DET | |  |  |  |  | 313/627 | | 49.9% | |  |  |  |  |  |  |  |  |  |
|  |  | 1 DET | |  | | 1 | | 7/23 | | 30.4% | | 1.17 (0.32, 4.25) | | 0.82 | | - | | - | |  |
|  |  | 1 TET (ref.) | |  |  |  |  | 6/22 | | 27.3% | |  |  |  |  |  |  |  |  |  |
|  |  | 1 DET | |  | | 1 | | 15/28 | | 53.6% | | 0.75 (0.26, 2.16) | | 0.59 | | - | | - | |  |
|  |  | 1 QET (ref.) | |  |  |  |  | 17/28 | | 60.7% | |  |  |  |  |  |  |  |  |  |
|  |  | 1 DET (ref.) | |  | | 1 | | 25/53 | | 47.2% | | 1.40 (0.65, 3.00) | | 0.39 | | - | | - | |  |
|  |  | 2 x SET | |  |  |  |  | 30/54 | | 55.6% | |  |  |  |  |  |  |  |  |  |
|  |  | 1 DET (ref.) | |  | | 1 | | 172/323 | | 53.3% | | 0.78 (0.90, 1.07) | | 0.12 | | - | | - | |  |
|  |  | 1 fresh SET + 1 frozen SET | |  |  |  |  | 152/322 | | 47.2% | |  |  |  |  |  |  |  |  |  |
| **Fresh embryo transfer in comparison to frozen embryo transfer** | | | | | | | | | | | | | | | | | | | |  |
| Roque et al. (2013)  *Meta-analysis* | • IVF/ICSI with cleavage or blastocyst stage embryos  • 1 cycle per woman/couple | Fresh ET (ref.)  Frozen ET | | Clinical pregnancy | | 3 | | 121/316  159/317 | | 38.3%  50.2% | | 1.31 (1.10, 1.56)† | | 0.002 | | 0% | | 0.74 | |  |
|  |  | Fresh ET (ref.)  Frozen ET | | Ongoing pregnancy | | 3 | | 113/316  150/317 | | 35.8%  47.3% | | 1.32 (1.10, 1.59)† | | 0.003 | | 0% | | 0.60 | |  |
| D’Angelo and Amso (2007)  *Meta-analysis* | • Autologous IVF/ICSI with cleavage stage (day 2-3) embryos  • 3-4 embryos per cycle  • 1 cycle per woman/couple | Fresh ET (ref.) | | Clinical pregnancy | | 1 | | 31/67 | | 46.3% | | 1.08 (0.54, 2.19) | | 0.82 | | - | | - | |  |
|  |  | Frozen ET | |  |  |  |  | 28/58 | | 48.3% | |  |  |  |  |  |  |  |  |  |
| **Stage of embryo during transfer** | | | | | | | | | | | | | | | | | | | |  |
| Glujovsky et al. (2012)  *Meta-analysis* | • Fresh, autologous or donor IVF/ICSI  • 1-5 embryos per cycle  • 1 or more cycles per woman/couple | Cleavage stage ET (ref.) | | Clinical pregnancy | | 23 | | 652/1679 | | 38.8% | | 1.14 (0.99, 1.32) | | 0.075 | | 47% | | 0.01 | |  |
|  |  | Blastocyst stage ET | |  |  |  |  | 653/1652 | | 39.5% | |  |  |  |  |  |  |  |  |  |
|  |  | Cleavage stage ET (ref.) | | Studies with equal number of cleavage and blastocyst stage embryos transferred | | 11 | | 339/937 | | 36.2% | | 1.19 (0.99, 1.44) | | 0.069 | | 35% | | 0.12 | |  |
|  |  | Blastocyst stage ET | |  |  |  |  | 370/917 | | 40.3% | |  |  |  |  |  |  |  |  |  |
|  |  | Cleavage stage ET (ref.) | | Studies with SET in both groups | | 3 | | 76/240 | | 31.7% | | 1.24 (0.84, 1.82) | | 0.28 | | 70% | | 0.04 | |  |
|  |  | Blastocyst stage ET | |  |  |  |  | 85/238 | | 35.7% | |  |  |  |  |  |  |  |  |  |
|  |  | Cleavage stage ET (ref.) | | Studies with more cleavage-stage embryos transferred than blastocyst stage | | 12 | | 313/742 | | 42.2% | | 1.07 (0.86, 1.33) | | 0.54 | | 55% | | 0.01 | |  |
|  |  | Blastocyst stage ET | |  |  |  |  | 283/645 | | 43.9% | |  |  |  |  |  |  |  |  |  |
|  |  | Cleavage stage ET (ref.) | | Studies limited to patients with a good prognosis | | 14 | | 361/885 | | 40.8% | | 1.15 (0.83, 1.58) | | 0.41 | | 57% | | 0.004 | |  |
|  |  | Blastocyst stage ET | |  |  |  |  | 398/871 | | 45.7% | |  |  |  |  |  |  |  |  |  |
|  |  | Cleavage stage ET (ref.) | | Studies limited to patients with a poor prognosis | | 2 | | 5/43 | | 11.6% | | 2.59 (0.75, 8.92) | | 0.13 | | 0 | | 0.39 | |  |
|  |  | Blastocyst stage ET | |  |  |  |  | 9/34 | | 26.5% | |  |  |  |  |  |  |  |  |  |
|  |  | Cleavage stage ET (ref.) | | Studies with unselected patients | | 7 | | 286/751 | | 38.1% | | 1.01 (0.81, 1.25) | | 0.95 | | 0 | | 0.52 | |  |
|  |  | Blastocyst stage ET | |  |  |  |  | 246/657 | | 37.4% | |  |  |  |  |  |  |  |  |  |
|  |  | Cleavage stage ET | | Cumulative clinical pregnancies‡ | | 4 | | 144/257 | | 56.0% | | 1.58 (1.11, 2.25) | | 0.011 | | 46% | | 0.14 | |  |
|  |  | Blastocyst stage ET (ref.) | |  |  |  |  | 122/270 | | 45.2% | |  |  |  |  |  |  |  |  |  |
| Papanikolaou et al. (2008)  *Meta-analysis* | • Fresh, autologous or donor IVF/ICSI  • 1-5 embryos per cycle  • 1 cycle per woman/couple | Cleavage stage ET (ref.) | | Clinical pregnancy in RCTs | | 8 | | 284/846 | | 33.6% | | 1.27 (1.03, 1.55) | | 0.02 | | 27% | | 0.22 | |  |
|  |  | Blastocyst stage ET | |  |  |  |  | 323/826 | | 39.1% | |  |  |  |  |  |  |  |  |  |
|  |  | Cleavage stage ET | | RCTs and pseudo-randomized trial | | 9 | | 315/945 | | 33.3% | | 1.29 (1.07, 1.56) | | 0.008 | | 19% | | 0.28 | |  |
|  |  | Blastocyst stage ET | |  |  |  |  | 375/954 | | 39.3% | |  |  |  |  |  |  |  |  |  |
| **IVF/ICSI in comparison to spontaneous conception** | | | | | | | | | | | | | | | | | | | | |
| Pandian et al. (2011)  *Meta-analysis* | • Fresh, autologous IVF with cleavage stage (day 2-3) or blastocyst (day 5-6) stage embryos  • 1 cycle per woman/couple | | SC (3-6 months) (ref.) | | Clinical pregnancy | | 2 | | 5/41 | | 12.2% | | 3.24 (1.07, 9.80) | | 0.037 | | 80% | | 0.03 | |
|  |  |  | IVF | |  |  |  |  | 13/45 | | 28.9% | |  |  |  |  |  |  |  |  |
| * Biochemical pregnancy = positive urine or serum β-hCG levels 14 days after embryo transfer; clinical pregnancy = ultrasound visualization of gestational sac(s) with fetal pole and fetal heart movements/activity/beat; ongoing pregnancy = ultrasound visualization of gestational sac(s) with a visible fetal heart beat after 12 weeks gestation  † Risk ratio  ‡ Cumulative clinical pregnancy rate per woman from all fresh and frozen cycles | | | | | | | | | | | | | | | | | | | |  |
