# Supplementary material for: Assisted reproductive technologies (ARTs): Evaluation of evidence to support public policy development
Source: Reprod Health. 2014 Nov 7;11:76. doi: 10.1186/1742-4755-11-76 (PMC4233043; doi:10.1186/1742-4755-11-76)
Supplement: Supplementary file 13 — Additional file 13: Table S13: Effectiveness: miscarriage rate. (DOC 52 KB) [file 12978_2014_327_MOESM13_ESM.doc]

## Additional file 13: Table S13. Effectiveness: miscarriage rate

| **Review** | **Treatment Characteristics** | **Study Groups** | **Subgroups** | **Number of primary studies** | **Miscarriage rate*** | | | | **Heterogeneity** | |
| --- | --- | --- | --- | --- | --- | --- | --- | --- | --- | --- |
|  |  |  |  |  | **n/N** | **%** | **Odds Ratio**  **(95% CI)** | **P-value** | **I^2^ (%)** | **P-value** |
| **IVF in comparison to other treatment options** | | | | | | | | | | |
| Pandian et al. (2011)  *Meta-analysis* | • Fresh, autologous IVF with cleavage stage (day 2-3) or blastocyst (day 5-6) stage embryos  • 1-6 cycles per woman/couple | FSH-IUI (≤3 cycles) (ref.) | Clinical pregnancy  Women who failed to achieve pregnancy with ≤3 cycles of CC-IUI | 1 | 7/50 | 14.0% | nr | nr | - | - |
|  |  | IVF (≤6 cycles) |  |  | 27/145 | 18.6% |  |  |  |  |
| **Fresh vs. Frozen embryo transfer** | | | | | | | | | | |
| Roque et al. (2013)  *Meta-analysis* | IVF/ICSI with cleavage or blastocyst embryos | Fresh ET (ref.)  Frozen ET | Miscarriage rate | 3 | 18/316  15/317 | 5.7%  4.7% | 0.83 (0.43, 1.60)† | 0.57 | 0% | 1.00 |
| **Number of embryos transferred** | | | | | | | | | | |
| Grady et al. (2012)  *Meta-analysis* | • Fresh, autologous or donor IVF/ICSI with cleavage or blastocyst stage embryos  • 1 cycle per woman/couple | eSET (ref.) | RCTs | 4 | 44/216 | 20.4% | 0.74 (0.43, 1.27)‡ | nr (not sig.) | 38% | nr |
|  |  | DET |  |  | 46/305 | 15.1% |  |  |  |  |
|  |  | eSET (ref.) | Cohorts | 4 | 53/361 | 14.7% | 0.88 (0.58, 1.33)‡ | nr (not sig.) | 30% | nr |
|  |  | DET |  |  | 87/656 | 13.3% |  |  |  |  |
| McLernon et al. (2010)†  *Meta-analysis* | • Fresh, autologous IVF/ICSI with cleavage stage (day 2-3) embryos  • 1 cycle per woman/couple | eSET (ref.) |  | nr | 60/245 | 24.5% | 0.66 (0.44, 0.99) | 0.06 | nr (sig. het.) | nr |
|  |  | DET |  |  | 63/355 | 17.7% |  |  |  |  |
| Gelbaya et al. (2010)  *Meta-analysis* | • Fresh, autologous IVF/ICSI with cleavage stage (day 2-3) embryos  • 1 cycle per woman/couple | eSET (ref.) |  | 4 | 42/612 | 6.9% | 1.10 (0.74, 1.64)‡ | 0.65 | nr | nr |
|  |  | DET |  |  | 46/608 | 7.6% |  |  |  |  |
| Pandian et al. (2009)  *Meta-analysis* | • Fresh, autologous or donor IVF/ICSI with cleavage stage (day 2-3) embryos  • 1-2 cycles per woman/couple | 1 SET (ref.) |  | 3 | 37/550 | 6.7% | 1.18 (0.75, 1.86) | 0.48 | 61% | 0.08 |
|  |  | 1 DET |  |  | 43/547 | 7.9% |  |  |  |  |
|  |  | 1 DET (ref.) |  | 1 | 5/53 | 9.4% | 1.67 (0.51, 5.48) | 0.40 | - | - |
|  |  | 2 x SET |  |  | 8/54 | 14.8% |  |  |  |  |
| **Stage of embryo during transfer** | | | | | | | | | | |
| Glujovsky et al. (2012)  *Meta-analysis* | • Fresh, autologous or donor IVF/ICSI  • 1-5 embryos per cycle  • 1 or more cycles per woman/couple | Cleavage stage ET (ref.) |  | 14 | 86/1069 | 8.0% | 1.14 (0.84, 1.55) | 0.39 | 0 | 0.64 |
|  |  | Blastocyst stage ET |  |  | 97/1058 | 9.5% |  |  |  |  |
|  |  | Smokers |  |  | nr | nr |  |  |  |  |
| * Number of miscarriages per pregnancy in Grady et al. (2010), McLernon et al. (2010), Maheshwari et al. (2007), and Pandian et al. (2009), and per woman/couple in Gelbaya et al. (2010) and Glujovsky et al. (2012).  † Meta-analysis of individual patient data  ‡ Risk ratio | | | | | | | | | | |
