# Supplementary material for: Assisted reproductive technologies (ARTs): Evaluation of evidence to support public policy development
Source: Reprod Health. 2014 Nov 7;11:76. doi: 10.1186/1742-4755-11-76 (PMC4233043; doi:10.1186/1742-4755-11-76)
Supplement: Supplementary file 14 — Additional file 14: Table S14: Effectiveness: live birth rate. (DOC 120 KB) [file 12978_2014_327_MOESM14_ESM.doc]

## Additional file 14: Table S14. Effectiveness: live birth rate

| **Review** | **Treatment Characteristics** | **Study Groups** | | **Subgroups** | | **Number of primary studies** | | **Live birth rate per woman or couple** | | | | | | | | **Heterogeneity** | | | |
| --- | --- | --- | --- | --- | --- | --- | --- | --- | --- | --- | --- | --- | --- | --- | --- | --- | --- | --- | --- |
|  |  |  |  |  |  |  |  | **n/N** | | **%** | | **Odds Ratio**  **(95% CI)** | | **P-value** | | **I^2^ (%)** | | **P-value** | |
| **IVF in comparison to other treatment options** | | | | | | | | | | | | | | | | | | | |
| Pandian et al. (2011)  *Meta-analysis* | • Fresh or frozen, autologous IVF with cleavage stage (day 2-3) or blastocyst (day 5-6) stage embryos  • 1-6 cycles per woman/couple | sIUI (≤6 cycles) (ref.) | | Cumulative live birth rate*  Treatment-naïve women | | 2 | | 34/117 | | 29.1% | | 1.09 (0.74, 1.59) | | 0.66 | | 0 | | 0.99 | |
|  |  | IVF (≤6 cycles) | |  |  |  |  | 37/117 | | 31.6% | |  |  |  |  |  |  |  |  |
|  |  | FSH-IUI (≤3 cycles) (ref.) | | Cumulative live birth rate*  Women who failed to achieve pregnancy with ≤3 cycles of CC-IUI | | 1 | | 37/169 | | 21.9% | | 2.66 (1.94, 3.63) | | <0.00001 | | - | | - | |
|  |  | IVF (≤6 cycles) | |  |  |  |  | 100/172 | | 58.1% | |  |  |  |  |  |  |  |  |
| **Number of embryos transferred** | | | | | | | | | | | | | | | | | | | |
| McLernon et al. (2010)†  *Meta-analysis* | • Fresh, autologous IVF/ICSI with cleavage stage (day 2-3) embryos  • 1 cycle per woman/couple | eSET | |  | | 8 | | 181/683 | | 26.5% | | 0.50 (0.39, 0.63)‡ | | <0.001 | | 0 | | 0.77 | |
|  |  | DET (ref.) | |  |  |  |  | 285/683 | | 41.7% | |  |  |  |  |  |  |  |  |
|  |  | eSET | | Age <33 years | | 8 | | 131/461 | | 28.4% | | 0.48 (0.36, 0.63) | | nr | | - | | - | |
|  |  | DET (ref.) | |  |  |  |  | 205/454 | | 45.2% | |  |  |  |  |  |  |  |  |
|  |  | eSET | | Age ≥33 years | |  |  | 50/219 | | 22.8% | | 0.54 (0.36, 0.83) | | nr | | - | | - | |
|  |  | DET (ref.) | |  |  |  |  | 80/277 | | 28.9% | |  |  |  |  |  |  |  |  |
|  |  | eSET | | Grade A embryos | | 8 | | 164/571 | | 28.7% | | 0.53 (0.40, 0.67) | | nr | | - | | - | |
|  |  | DET (ref.) | |  |  |  |  | 259/597 | | 43.4% | |  |  |  |  |  |  |  |  |
|  |  | eSET | | Grade B embryos | |  |  | 10/79 | | 12.7% | | 0.29 (0.11, 0.71) | | nr | | - | | - | |
|  |  | DET (ref.) | |  |  |  |  | 17/51 | | 33.3% | |  |  |  |  |  |  |  |  |
|  |  | eSET | | Duration of infertility <3 years | | 8 | | 60/234 | | 25.6% | | 0.49 (0.33, 0.72) | | nr | | - | | - | |
|  |  | DET (ref.) | |  |  |  |  | 93/226 | | 41.2% | |  |  |  |  |  |  |  |  |
|  |  | eSET | | Duration of infertility ≥3 years | |  |  | 115/432 | | 26.6% | | 0.51 (0.38, 0.69) | | nr | | - | | - | |
|  |  | DET (ref.) | |  |  |  |  | 183/441 | | 41.5% | |  |  |  |  |  |  |  |  |
|  | • 1-2 cycles per woman/couple | eSET | | Cumulative live birth rate* | | 2 | | 132/350 | | 37.7% | | 0.85 (0.62, 1.15) | | nr | | nr | | nr | |
|  |  | DET (ref.) | |  |  |  |  | 149/353 | | 42.2% | |  |  |  |  |  |  |  |  |
| Gelbaya et al. (2010)  *Meta-analysis* | • Fresh, autologous IVF/ICSI with cleavage stage (day 2-3) embryos  • 1 cycle per woman/couple | eSET | |  | | 5 | | 171/652 | | 26.2% | | 0.62 (0.53, 0.72)‡ | | <0.00001 | | 37% | | 0.17 | |
|  |  | DET (ref.) | |  |  |  |  | 275/649 | | 42.4% | |  |  |  |  |  |  |  |  |
| Baruffi et al. (2009)  *Meta-analysis* | • Fresh, autologous IVF/ICSI with cleavage (day 2-3) or blastocyst (day 5-6) stage embryos  • 1 cycle per woman/couple in most (2 studies with 1-2 cycles per woman) | SET (ref.) | |  | | 5 | | 149/524 | | 28.4% | | 1.87 (1.44, 2.42) | | <0.001 | | 0 | | 0.40 | |
|  |  | DET | |  |  |  |  | 222/522 | | 42.5% | |  |  |  |  |  |  |  |  |
|  |  | SET (ref.) | | Studies with patients ≤35 years | | 3 | | 127/450 | | 28.2% | | 1.91 (1.45, 2.53) | | <0.0001 | | nr (no het.) | | nr | |
|  |  | DET | |  |  |  |  | 194/452 | | 42.9% | |  |  |  |  |  |  |  |  |
| Pandian et al. (2009)  *Meta-analysis* | • Fresh, autologous or donor IVF/ICSI with cleavage stage (day 2-3) embryos  • 1 cycle per woman/couple | SET (ref.) | |  | | 5 | | 166/630 | | 26.3% | | 2.10 (1.65, 2.66) | | <0.00001 | | 0 | | 0.51 | |
|  |  | DET | |  |  |  |  | 268/627 | | 42.7% | |  |  |  |  |  |  |  |  |
|  |  | DET | |  | | 1 | | 3/23 | | 13.0% | | 0.40 (0.9, 1.85) | | 0.24 | | - | | - | |
|  |  | TET (ref.) | |  |  |  |  | 6/22 | | 27.3% | |  |  |  |  |  |  |  |  |
|  |  | DET | |  | | 1 | | 8/28 | | 28.6% | | 0.35 (0.11, 1.05) | | 0.61 | | - | | - | |
|  |  | QET (ref.) | |  |  |  |  | 15/28 | | 53.6% | |  |  |  |  |  |  |  |  |
|  | • 1-3 cycles per woman/couple | 1 DET (ref.) | | Cumulative live birth rate* | | 1 | | 19/53 | | 35.8% | | 1.23 (0.56, 2.69) | | 0.60 | | - | | - | |
|  |  | 2 x SET | |  |  |  |  | 22/54 | | 40.7% | |  |  |  |  |  |  |  |  |
|  |  | 1 DET (ref.) | | Cumulative live birth rate* | | 1 | | 140/323 | | 43.3% | | 0.81 (0.59, 1.11) | | 0.28 | | - | | - | |
|  |  | 1 fresh SET + 1 frozen SET | |  |  |  |  | 123/322 | | 38.2% | |  |  |  |  |  |  |  |  |
|  |  | 2 x DET (ref.) | | Cumulative live birth rate* | | 1 | | 7/23 | | 30.4% | | 0.77 (0.22, 2.65) | | 0.67 | | - | | - | |
|  |  | 2 x TET | |  |  |  |  | 8/22 | | 36.4% | |  |  |  |  |  |  |  |  |
|  |  | 3 x DET (ref.) | | Cumulative live birth rate* | | 1 | | 9/23 | | 39.1% | | 0.77 (0.24, 2.52) | | 0.67 | | - | | - | |
|  |  | 3 x TET | |  |  |  |  | 10/22 | | 45.5% | |  |  |  |  |  |  |  |  |
| **Fresh embryo transfer in comparison to frozen embryo transfer** | | | | | | | | | | | | | | | | | | | |
| D’Angelo and Amso (2007)  *Meta-analysis* | • Autologous IVF/ICSI with cleavage stage (day 2-3) embryos  • 3-4 embryos per cycle  • 1 cycle per woman/couple | Fresh ET (ref.) | |  | | 1 | | 25/67 | | 37.3% | | 1.03 (0.50, 2.12) | | 0.94 | | - | | - | |
|  |  | Frozen ET | |  |  |  |  | 22/58 | | 37.9% | |  |  |  |  |  |  |  |  |
| **Stage of embryo during transfer** | | | | | | | | | | | | | | | | | | | |
| Glujovsky et al. (2012)  *Meta-analysis* | • Fresh, autologous or donor IVF/ICSI  • 1-5 embryos per cycle  • 1 or more cycles per woman/couple | Cleavage stage ET (ref.) | |  | | 12 | | 237/759 | | 31.2% | | 1.40 (1.13, 1.74) | | 0.0021 | | 40% | | 0.07 | |
|  |  | Blastocyst stage ET | |  |  |  |  | 292/751 | | 38.9% | |  |  |  |  |  |  |  |  |
|  |  | Cleavage stage ET (ref.) | | Studies with equal number of cleavage and blastocyst stage embryos transferred | | 6 | | 164/515 | | 31.8% | | 1.35 (1.04, 1.75) | | 0.023 | | 41% | | 0.13 | |
|  |  | Blastocyst stage ET | |  |  |  |  | 197/512 | | 38.5% | |  |  |  |  |  |  |  |  |
|  |  | Cleavage stage ET (ref.) | | Studies with SET in both groups | | 2 | | 59/228 | | 25.9% | | 1.46 (0.98, 2.19) | | 0.064 | | 29% | | 0.24 | |
|  |  | Blastocyst stage ET | |  |  |  |  | 78/230 | | 33.9% | |  |  |  |  |  |  |  |  |
|  |  | Cleavage stage ET (ref.) | | Studies with more cleavage-stage embryos transferred than blastocyst stage | | 6 | | 73/244 | | 29.9% | | 1.52 (1.03, 2.23) | | 0.033 | | 48% | | 0.09 | |
|  |  | Blastocyst stage ET | |  |  |  |  | 95/239 | | 39.7% | |  |  |  |  |  |  |  |  |
|  |  | Cleavage stage ET (ref.) | | Studies limited to patients with a good prognosis | | 8 | | 175/561 | | 31.2% | | 1.43 (0.99, 2.07) | | 0.058 | | 49% | | 0.06 | |
|  |  | Blastocyst stage ET | |  |  |  |  | 229/565 | | 40.5% | |  |  |  |  |  |  |  |  |
|  |  | Cleavage stage ET (ref.) | | Studies limited to patients with a poor prognosis | | 2 | | 4/43 | | 9.3% | | 1.99 (0.49, 8.04) | | 0.33 | | 0 | | 0.48 | |
|  |  | Blastocyst stage ET | |  |  |  |  | 6/34 | | 17.6% | |  |  |  |  |  |  |  |  |
|  |  | Cleavage stage ET (ref.) | | Studies with unselected patients | | 2 | | 58/155 | | 37.4% | | 1.05 (0.56, 1.97) | | 0.88 | | 43% | | 0.19 | |
|  |  | Blastocyst stage ET | |  |  |  |  | 57/152 | | 37.5% | |  |  |  |  |  |  |  |  |
| Papanikolaou et al. (2008)  *Meta-analysis* | • Fresh, autologous or donor IVF/ICSI  • 1-5 embryos per cycle  • 1 cycle per woman/couple | Cleavage stage ET (ref.) | |  | | 6 | | 192/688 | | 27.9% | | 1.39 (1.10, 1.76) | | 0.005 | | 17% | | 0.30 | |
|  |  | Blastocyst stage ET | |  |  |  |  | 233/665 | | 35.0% | |  |  |  |  |  |  |  |  |
| **Embryo donation** | | | | | | | | | | | | | | | | | | | |
| Keenan et al. (2012)  *Primary Study* |  | Fresh autologous embryos (ref.) | |  | | 1 | | 272754/ 876883 | | 31.1% | |  | |  | |  | |  | |
|  |  | Donor embryos | |  |  |  |  | 1972/7042 | | 28.0% | | 0.90 (0.87, 0.93) | | nr | | n/a | | n/a | |
|  |  | Frozen autologous embryos | |  |  |  |  | 57698/ 268986 | | 21.5% | | 0.69 (0.68, 0.70) | | nr | | n/a | | n/a | |
|  |  | Fresh donor oocytes | |  |  |  |  | 37937/ 81050 | | 46.8% | | 1.50 (1.49, 1.52) | | nr | | n/a | | n/a | |
|  |  | Frozen donor oocytes | |  |  |  |  | 9543/  35967 | | 26.5% | | 0.90 (0.89, 0.92) | | nr | | n/a | | n/a | |
| **IVF/ICSI in comparison to spontaneous conception** | | | | | | | | | | | | | | | | | | | |
| Pandian et al. (2011)  *Meta-analysis* | • Fresh, autologous IVF with cleavage stage (day 2-3) or blastocyst (day 5-6) stage embryos  • 1 cycle per woman/couple | | IVF (ref.) | |  | | 1 | | 1/27 | | 3.7% | | 22.00 (2.56, 189.37) | | 0.0049 | | - | | - |
|  |  |  | SC (3-6 months) | |  |  |  |  | 11/24 | | 45.8% | |  |  |  |  |  |  |  |
| * Cumulative rates include all cycles (transfers) per woman resulting from a single oocyte retrieval; in McLernon et al. (2010), 1 additional frozen SET cycle after a single fresh SET cycle was compared to a single DET cycle in both studies; in Pandian et al. (2009), 1 additional frozen SET cycle after a single fresh SET cycle was compared to a single DET in 1 study, 2 fresh SET cycles were compared to a single DET cycle in 1 study, and 2-3 fresh DET cycles were compared to 2-3 fresh TET cycles in 1 study  † Meta-analysis of individual patient data  ‡ Adjusted for significant covariates (grade of the embryos transferred, type of Treatment Characteristics, and the primary cause of infertility)  § Live birth rate per cycle  ** Risk ratio | | | | | | | | | | | | | | | | | | | |
