# Supplementary material for: Assisted reproductive technologies (ARTs): Evaluation of evidence to support public policy development
Source: Reprod Health. 2014 Nov 7;11:76. doi: 10.1186/1742-4755-11-76 (PMC4233043; doi:10.1186/1742-4755-11-76)
Supplement: Supplementary file 15 — Additional file 15: Table S15: Effectiveness: multiple pregnancy rate. (DOC 81 KB) [file 12978_2014_327_MOESM15_ESM.doc]

## Additional file 15: Table S15. Effectiveness: multiple pregnancy rate

| **Review** | **Treatment Characteristics** | **Study Groups** | **Subgroups** | **Number of primary studies** | **Multiple pregnancy rate*** | | | | **Heterogeneity** | |
| --- | --- | --- | --- | --- | --- | --- | --- | --- | --- | --- |
|  |  |  |  |  | **n/N** | **%** | **Odds Ratio**  **(95% CI)** | **P-value** | **I^2^ (%)** | **P-value** |
| **IVF in comparison to other treatment options** | | | | | | | | | | |
| Pandian et al. (2011)  *Meta-analysis* | • Fresh or frozen, autologous IVF with cleavage stage (day 2-3) or blastocyst (day 5-6) stage embryos  • 1-6 cycles per woman/couple | sIUI (≤6 cycles) (ref.) | Clinical pregnancy  Treatment-naïve women | 3 | 23/175 | 13.1% | 0.64 (0.31, 1.29) | 0.21 | 0 | 1.00 |
|  |  | IVF (≤6 cycles) |  |  | 16/176 | 9.1% |  |  |  |  |
| **Number of embryos transferred** | | | | | | | | | | |
| Pandian et al. (2009)  *Meta-analysis* | • Fresh, autologous or donor IVF/ICSI with cleavage stage (day 2-3) embryos  • 1-2 cycles per woman/couple | 1 SET (ref.) |  | 5† | 3/431 | 0.7% | 0.04 (0.01, 0.11) | <0.00001 | 0 | 0.67 |
|  |  | 1 DET |  |  | 83/445 | 18.7% |  |  |  |  |
|  |  | 1 DET |  | 1 | 0/23 | 0 | 0.17 (0.01, 3.85) | 0.27 | - | - |
|  |  | 1 TET (ref.) |  |  | 2/22 | 9.1% |  |  |  |  |
|  |  | 1 DET |  | 1 | 3/28 | 10.7% | 0.44 (0.10, 1.97) | 0.28 | - | - |
|  |  | 1 QET (ref.) |  |  | 6/28 | 21.4% |  |  |  |  |
|  |  | 1 DET (ref.) |  | 1 | 7/53 | 13.2% | 0.06 (0.00, 1.02) | 0.052 | - | - |
|  |  | 2 x SET |  |  | 0/54 | 0 |  |  |  |  |
| **Stage of embryo during transfer** | | | | | | | | | | |
| Glujovsky et al. (2012)  *Meta-analysis* | • Fresh, autologous or donor IVF/ICSI  • 1-5 embryos per cycle  • 1 or more cycles per woman/couple | Cleavage stage ET (ref.) |  | 16 | 136/1248 | 10.9% | 0.92 (0.71, 1.19) | 0.50 | 27% | 0.15 |
|  |  | Blastocyst stage ET |  |  | 128/1233 | 10.4% |  |  |  |  |
|  |  | Cleavage stage ET (ref.) | Studies with equal number of cleavage and blastocyst stage embryos transferred | 8 | 78/846 | 9.2% | 1.05 (0.75, 1.46) | 0.78 | 8% | 0.37 |
|  |  | Blastocyst stage ET |  |  | 81/826 | 9.8% |  |  |  |  |
|  |  | Cleavage stage ET (ref.) | Studies with SET in both groups | 1 | 2/176 | 1.1% | 0.20 (0.01, 4.17) | 0.30 | - | - |
|  |  | Blastocyst stage ET |  |  | 0/175 | 0 |  |  |  |  |
|  |  | Cleavage stage ET (ref.) | Studies with more cleavage-stage embryos transferred than blastocyst stage | 8 | 58/402 | 14.4% | 0.75 (0.49, 1.13) | 0.16 | 20% | 0.27 |
|  |  | Blastocyst stage ET |  |  | 47/407 | 11.5% |  |  |  |  |
|  |  | Cleavage stage ET (ref.) | Studies limited to patients with a good prognosis | 11 | 86/746 | 11.5% | 0.88 (0.63, 1.22) | 0.45 | 35% | 0.12 |
|  |  | Blastocyst stage ET |  |  | 81/752 | 10.8% |  |  |  |  |
|  |  | Cleavage stage ET (ref.) | Studies limited to patients with a poor prognosis | 1 | 3/31 | 9.7% | 0.89 (0.14, 5.81) | 0.90 | - | - |
|  |  | Blastocyst stage ET |  |  | 2/23 | 8.7% |  |  |  |  |
|  |  | Cleavage stage ET (ref.) | Studies with unselected patients | 4 | 48/471 | 10.2% | 0.96 (0.62, 1.47) | 0.84 | 0 | 0.70 |
|  |  | Blastocyst stage ET |  |  | 45/458 | 9.8% |  |  |  |  |
|  |  | Cleavage stage ET (ref.) | Higher-order (>2) multiple pregnancy rate | 12 | 9/1031 | 0.9% | 0.44 (0.15, 1.33) | 0.15 | 0 | 0.68 |
|  |  | Blastocyst stage ET |  |  | 3/1004 | 0.3% |  |  |  |  |
|  |  | Cleavage stage ET (ref.) | Studies with equal number of cleavage and blastocyst stage embryos transferred | 8 | 1/846 | 0.1% | 0.33 (0.01, 8.28) | 0.50 | 0 | 1.00 |
|  |  | Blastocyst stage ET |  |  | 0/826 | 0 |  |  |  |  |
|  |  | Cleavage stage ET (ref.) | Studies with more cleavage-stage embryos transferred than blastocyst stage | 4 | 8/185 | 4.3% | 0.46 (0.14, 1.49) | 0.19 | 0 | 0.52 |
|  |  | Blastocyst stage ET |  |  | 3/178 | 1.7% |  |  |  |  |
|  |  | Cleavage stage ET (ref.) | Studies limited to patients with a good prognosis | 9 | 9/700 | 1.3% | 0.29 (0.08, 1.06) | 0.061 | 0 | 1.00 |
|  |  | Blastocyst stage ET |  |  | 2/685 | 0.3% |  |  |  |  |
|  |  | Cleavage stage ET (ref.) | Studies limited to patients with a poor prognosis | 1 | 0/31 | 0 | 4.20 (0.16, 107.89) | 0.39 | - | - |
|  |  | Blastocyst stage ET |  |  | 1/23 | 4.3% |  |  |  |  |
|  |  | Cleavage stage ET (ref.) | Studies with unselected patients | 2 | 0/296 | 0 | - | - | - | - |
|  |  | Blastocyst stage ET |  |  | 0/300 | 0 |  |  |  |  |
| Papanikolaou et al. (2008)  *Meta-analysis* | • Fresh, autologous or donor IVF/ICSI  • 1-5 embryos per cycle  • 1 cycle per woman/couple | Cleavage stage ET (ref.) |  | 7 | 73/257 | 28.4% | 0.86 (0.58, 1.29) | 0.46 | 0 | 0.57 |
|  |  | Blastocyst stage ET |  |  | 75/293 | 25.6% |  |  |  |  |
| * Number of multiple pregnancies per clinical pregnancy in Papanikolaou et al. (2008); number of multiple pregnancies per woman in Pandian et al. (2011), Pandian et al. (2009) (†except 1 study out of 5 comparing SET to DET that reported the number of multiple births per live birth) | | | | | | | | | | |
