# Supplementary material for: Assisted reproductive technologies (ARTs): Evaluation of evidence to support public policy development
Source: Reprod Health. 2014 Nov 7;11:76. doi: 10.1186/1742-4755-11-76 (PMC4233043; doi:10.1186/1742-4755-11-76)
Supplement: Supplementary file 16 — Additional file 16: Table S16: Effectiveness: multiple birth rate. (DOC 58 KB) [file 12978_2014_327_MOESM16_ESM.doc]

## Additional file 16: Table S16. Effectiveness: multiple birth rate

| **Review** | **Treatment Characteristics** | **Study Groups** | **Subgroups** | **Number of primary studies** | **Multiple birth rate per live birth** | | | | **Heterogeneity** | |
| --- | --- | --- | --- | --- | --- | --- | --- | --- | --- | --- |
|  |  |  |  |  | **n/N** | **%** | **Odds Ratio**  **(95% CI)** | **P-value** | **I^2^ (%)** | **P-value** |
| **Number of embryos transferred** | | | | | | | | | | |
| McLernon et al. (2010)*  *Meta-analysis* | • Fresh, autologous IVF/ICSI with cleavage stage (day 2-3) embryos  • 1 cycle per woman/couple | eSET |  | 8 | 3/181 | 1.7% | 0.04 (0.01, 0.12)† | <0.001 | nr | nr |
|  |  | DET (ref.) |  |  | 84/285 | 29.5% |  |  |  |  |
|  |  | eSET | Age <33 years | 8 | 3/131 | 2.3% | nr | nr | - | - |
|  |  | DET (ref.) |  |  | 61/205 | 29.8% |  |  |  |  |
|  |  | eSET | Age ≥33 years |  | 0/50 | 0% | nr | nr | - | - |
|  |  | DET (ref.) |  |  | 23/80 | 28.8% |  |  |  |  |
|  |  | eSET | Grade A embryos | 8 | 3/164 | 1.8% | nr | nr | - | - |
|  |  | DET (ref.) |  |  | 78/259 | 30.1% |  |  |  |  |
|  |  | eSET | Grade B embryos |  | 0/10 | 0% | nr | nr | - | - |
|  |  | DET (ref.) |  |  | 4/17 | 23.5% |  |  |  |  |
|  |  | eSET | Duration of infertility <3 years | 8 | 1/60 | 1.7% | 0.04 (0.01, 0.29) | nr | - | - |
|  |  | DET (ref.) |  |  | 27/93 | 29.0% |  |  |  |  |
|  |  | eSET | Duration of infertility ≥3 years |  | 2/115 | 1.7% | 0.04 (0.01, 0.17) | nr | - | - |
|  |  | DET (ref.) |  |  | 54/183 | 29.5% |  |  |  |  |
|  |  | eSET | Cumulative multiple births‡ | 2 | 1/132 | 0.8% | 0.02 (0.002, 0.12) | nr | nr | nr |
|  |  | DET (ref.) |  |  | 47/149 | 31.5% |  |  |  |  |
| Gelbaya et al. (2010)  *Meta-analysis* | • Fresh, autologous IVF/ICSI with cleavage stage (day 2-3) embryos  • 1 cycle per woman/couple | eSET |  | 5 | 2/171 | 1.2% | 0.06 (0.02, 0.18)§ | <0.00001 | 0 | 0.91 |
|  |  | DET (ref.) |  |  | 82/275 | 29.8% |  |  |  |  |
| Pandian et al. (2009)  *Meta-analysis* | • Fresh, autologous or donor IVF/ICSI with cleavage stage (day 2-3) embryos  • 1-2 cycles per woman/couple | 1 DET (ref.) |  | 1 | 46/141 | 32.6% | 0.02 (0.00, 0.12) | 0.000064 | - | - |
|  |  | 1 fresh SET + 1 frozen SET |  |  | 1/123 | 0.8% |  |  |  |  |
| **Embryo quality** | | | | | | | | | | |
| McLernon et al. (2010)*  *Meta-analysis* | • Fresh, autologous IVF/ICSI with cleavage stage (day 2-3) embryos  • 1-2 embryos per cycle  • 1 cycle per woman/couple | Grade A embryos |  | 8 | 81/423 | 19.1% | 1.59 (0.49, 5.17) | 0.44 | nr | nr |
|  |  | Grade B embryos (ref.) |  |  | 4/27 | 14.8% |  |  |  |  |
| * Meta-analysis of individual patient data  † Adjusted OR; no significant covariates found  ‡ Multiple births per cumulative live birth; cumulative rates include all cycles (transfers) per woman resulting from a single oocyte retrieval (1 additional frozen SET cycle after a single fresh SET cycle was compared to a single DET cycle in both studies)  § Risk ratio | | | | | | | | | | |
