# Supplementary figures and images for: Assisted reproductive technologies (ARTs): Evaluation of evidence to support public policy development
Source: Reprod Health. 2014 Nov 7;11:76. doi: 10.1186/1742-4755-11-76 (PMC4233043; doi:10.1186/1742-4755-11-76)

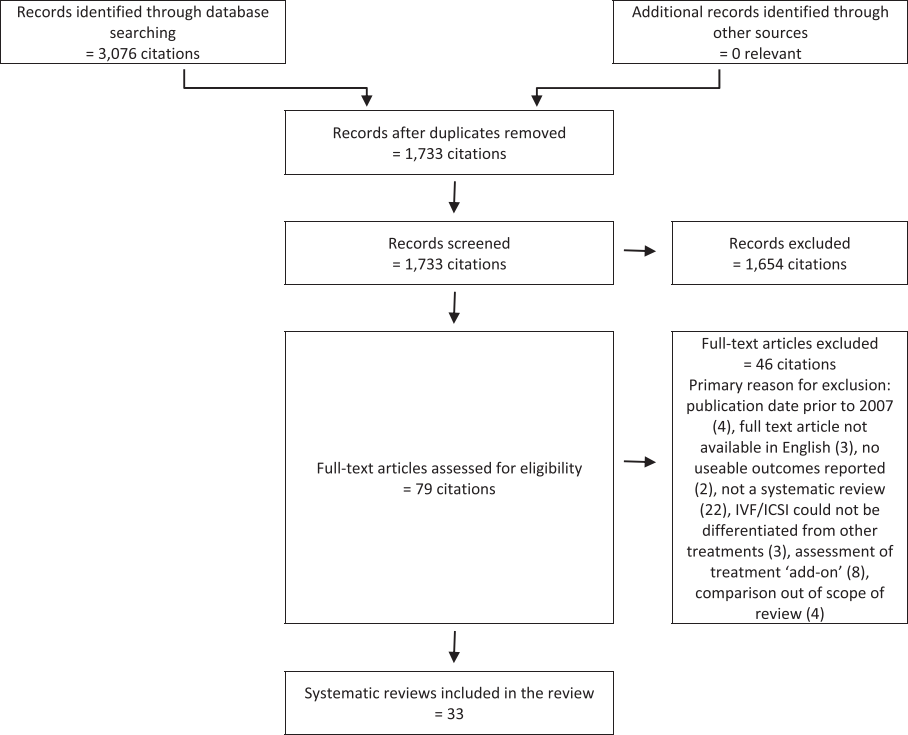

Supplement: Supplementary file 17 — Authors’ original file for figure 1 [file 12978_2014_327_MOESM17_ESM.pdf]

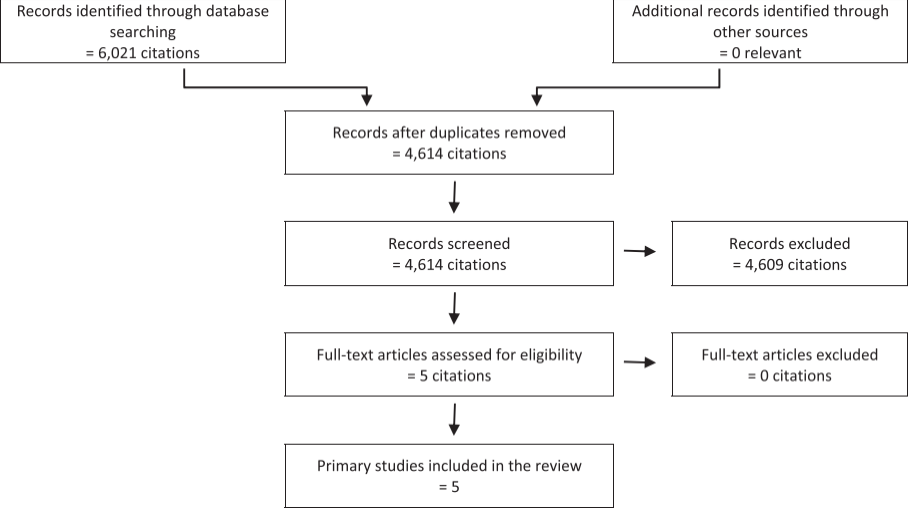

Supplement: Supplementary file 18 — Authors’ original file for figure 2 [file 12978_2014_327_MOESM18_ESM.pdf]

0% 20% 40% 60% 80% 100%

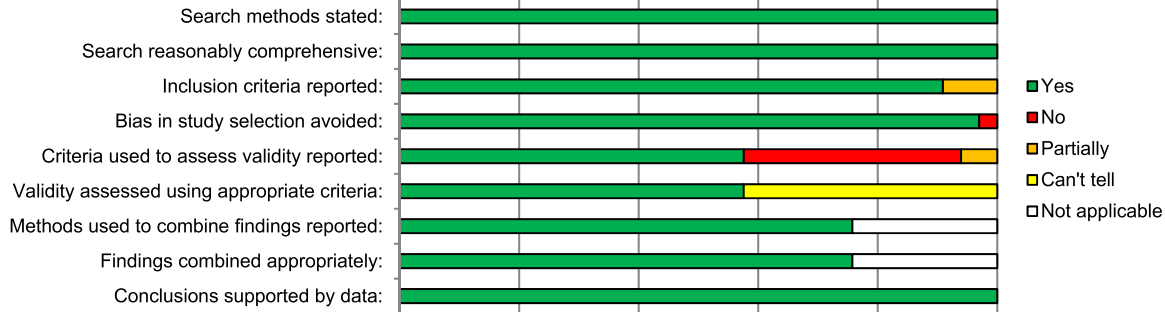

Supplement: Supplementary file 19 — Authors’ original file for figure 3 [file 12978_2014_327_MOESM19_ESM.pdf]
